# Marine Pyrrole Alkaloids

**DOI:** 10.3390/md19090514

**Published:** 2021-09-10

**Authors:** Kevin Seipp, Leander Geske, Till Opatz

**Affiliations:** Department of Chemistry, Organic Chemistry Section, Johannes Gutenberg University, Duesbergweg 10–14, 55128 Mainz, Germany; kseipp@uni-mainz.de (K.S.); legeske@uni-mainz.de (L.G.)

**Keywords:** pyrroles, alkaloids, marine natural products, nitrogen heterocycles, bromopyrroles, pyrrole-imidazole alkaloids, pyrrole-aminoimidazole alkaloids

## Abstract

Nitrogen heterocycles are essential parts of the chemical machinery of life and often reveal intriguing structures. They are not only widespread in terrestrial habitats but can also frequently be found as natural products in the marine environment. This review highlights the important class of marine pyrrole alkaloids, well-known for their diverse biological activities. A broad overview of the marine pyrrole alkaloids with a focus on their isolation, biological activities, chemical synthesis, and derivatization covering the decade from 2010 to 2020 is provided. With relevant structural subclasses categorized, this review shall provide a clear and timely synopsis of this area.

## 1. Introduction

The oceans cover more than 70% of the earth’s surface and comprise around 95% of the volume of the biosphere. This impressive size of the marine habitat and its biological diversity known to date lead to the assumption of an enormous, yet still largely unexplored world, carrying an unused potential for research areas such as pharmacology, medicine, crop protection, or food technology. Furthermore, the uniqueness of marine life is reflected by the fact that only a small fraction of the 30,000 marine natural products (MNPs) known at present can also be found in terrestrial sources [1]. Additionally, the isolation and investigation of MNPs is a rapidly expanding field of research at the interface of biology and chemistry [2,3,4,5,6,7,8,9,10]. Looking back to 2009, when only 20,000 MNPs were known, an impressive increase of 50% has been achieved in the past 11 years, which highlights the importance of the marine habitat in this context [11].

Among the marine alkaloids, which are largely composed of nitrogen-containing heterocycles, the pyrroles form a large group of intriguing natural products which occur in marine organisms ranging from microbes over algae and sponges to animals. Their structural diversity including terpenoid-, polyketide-, carbohydrate-, lipid-, and peptide-frameworks [7,12] accompanied by attractive biological properties, has spurred a considerable interest of chemists [6,13,14,15,16,17,18,19].

This review focuses on marine pyrrole alkaloids containing at least one pyrrole moiety, which were discovered during the decade of 2010 to 2020. The number of newly discovered pyrrole MNPs surged in this decade and many structural revisions resulted in a deeper knowledge of their biogenetic origin and structural relations.

In addition to the reported structures and their biological sources, known biological activities and, where applicable, the first total syntheses of these compounds will be shown. Furthermore, this review is subdivided by structural subclasses based on the substitution pattern of the pyrrole core. As a delineation, only MNPs with intact pyrrole functionality are described, whereas indole alkaloids [20], the saturated heterocycles pyrroline and pyrrolidine [21], as well as other fused systems (e.g., carbazoles) and pyrrole derivatives lacking a genuine pyrrole core [22,23,24,25], will not be covered. Several other specific overviews focusing on subclasses such as bromopyrroles [26,27] and pyrrole-imidazole alkaloids (PIA) [13,14,28] or with the focus on the isolation source [14,25,27], have been published. In contrast, we intend to provide the reader with an impression of the multiple facets of pyrrole alkaloids in the marine environment.

The five-membered planar 6π heteroaromatic pyrrole core with its high electron density is a reactive and privileged structural motif found in many biomolecules. It can provide stacking interactions, coordinate metal ions, or form hydrogen bonds when devoid of a substituent in the 1-position. Probably, the most well-known pyrrole derivatives in nature possess a tetrapyrrole skeleton, which can, e.g., be found in heme, chlorophyll, and several other porphyrinoid cofactors [29,30]. However, pyrroles possessing much simpler architectures have also attracted considerable interest, e.g., as promising lead structures in medicinal chemistry [15]. The biggest-selling drug of all time, the blood cholesterol lowering HMG-CoA reductase inhibitor atorvastatin (Lipitor^®^), is a pyrrole derivative. Not surprisingly, many pyrrole MNPs have also been associated with various pharmacological activities, such as cytotoxic [31,32], anti-bacterial [33,34], anti-fungal [35], and anti-cancer properties [6,36,37].

## 2. Non-Halogenated Marine Pyrrole Alkaloids

The alkaloids presented in this chapter are identified by a non-halogenated pyrrole core. Despite their structural diversity, the biosynthetic origin of these alkaloids can be traced back to a small number of possible biosynthetic pathways. According to the stunning logic of nature, only a few building blocks such as the amino acids glycine, serine, tryptophan, and proline are necessary to construct their pyrrole units.

A well-known pathway involves δ-aminolevulinic acid (ALA) as a key intermediate, which is produced from glycine and succinyl-CoA. An enzyme-catalyzed Knorr-type condensation–cyclization reaction of two molecules of δ-aminolevulinate yields porphobilinogen as a central intermediate, from which the trialkyl-substituted pyrroles are derived. Porphobilinogen is prone to self-condensation under acidic conditions and can further react to polypyrrolic systems, most notably the tetrapyrroles. Another major biosynthetic pathway is the dehydrogenation of proline to the common pyrrole-2-carboxylate unit. The activation of proline is suggested to involve a peptidyl carrier protein (PCP) forming a thioester linkage. In the next step, a controlled four-electron oxidation process with a flavoprotein desaturase occurs. These two C−N desaturation steps of the prolyl-*S*-PCP and subsequent tautomerization lead to the desired pyrrolyl-2-carboxyl-*S*-PCP product. Starting from this activated intermediate, a broad spectrum of reactions such as enzymatic transfer to nucleophiles or enzymatic halogenations can occur to create the world of marine pyrrole alkaloids [25,30,38,39].

### 2.1. Simple Pyrroles

**Figure 1:**
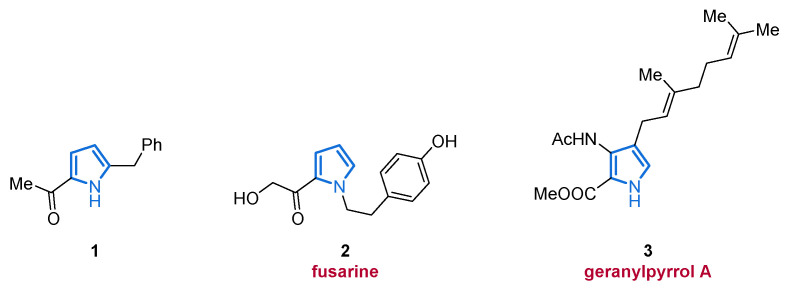
Simple pyrrole alkaloids **1**–**3** isolated from different marine organisms.

The pyrrole derivative 1-(4-benzyl-1*H*-pyrrol-3-yl)ethanone (**1**) was found in a co-culture of the marine-derived fungi *Aspergillus sclerotiorum* and *Penicillium citrinum* in 2017 (Figure 1). The acylated pyrrole **1** shows only medium toxicity against brine shrimp (LC_50_ values of 46.2 µM) and oppositely increases the growth of *Staphylococcus aureus* at 100 µg/mL [40].

Investigation of an endophytic strain of *Fusarium incarnatum* yielded another acylated pyrrole, fusarine (**2**), isolated from the marine mangrove fruit *Aegiceras corniculatum* in 2012 (Figure 1). Alkaloid **2** is expected to be formed biosynthetically via a Paal–Knorr cyclization of a primary amine and a 1,3-dicarbonyl, but showed neither antiproliferative nor cytotoxic potential against HUVEC, K-562, and HeLa human cell lines [41].

Another simple pyrrole is represented by geranylpyrrol A (**3**), which is counted among the small class of pyrrolomonoterpenoids and derives from pyrrolostatin (Figure 1). It was isolated from a mutant strain of *Streptomyces* sp. CHQ-64 in 2017 but did not display any toxicity against eight tested human cancer cell lines [42].

**Scheme 1:**
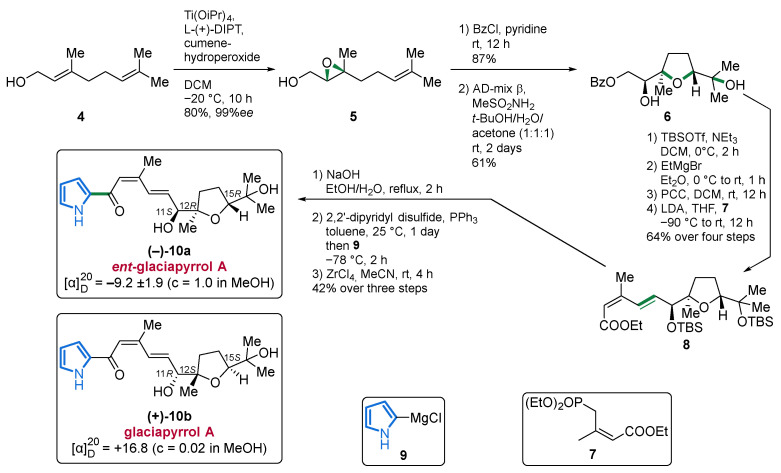
Enantioselective approach towards the total synthesis of pyrrolosesquiterpenoid **10b** by a Sharpless epoxidation/dihydroxylation sequence, leading to the unnatural *ent*-(−)-glaciapyrrol A (**10a**).

The pyrroloterpenoid glaciapyrrol A (**10b**) was already isolated along with its congeners glaciapyrrols B and C in 2005. Despite extensive investigations, the relative configuration of C-11 and the overall absolute configuration could not be determined at this time [43]. Through the first total synthesis of its four diastereomers by Dickschat in 2011, the relative configuration of the three stereocenters could be unequivocally established [44]. The authors devised an enantioselective synthesis starting from geraniol (**4**) using a Sharpless epoxidation to furnish alcohol **5**. Protection of the alcohol functionality and subsequent Sharpless dihydroxylation followed by intramolecular cyclization served as the key step and stereoselectively generated compound **6**. After several steps including a protection/deprotection sequence followed by oxidation and Horner–Wadsworth–Emmons (HWE) reaction using phosphonate **7**, ester **8** was obtained in 64% over four steps. Saponification, the addition of pyrrolyl Grignard **9,** and final TBS-deprotection finally produced *ent*-(−)-glaciapyrrol A (**10a**) showing the opposite optical rotation as the original publication from 2005. The authors, therefore, identified the natural product as (+)-glaciapyrrol A (**10b**) (Scheme 1) [44].

**Figure 2:**
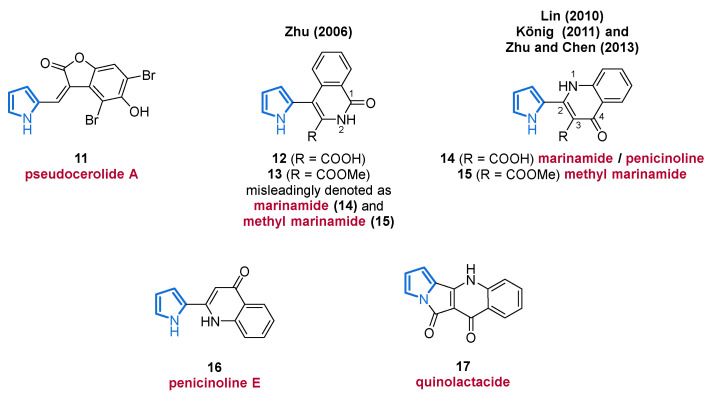
Pseudocerolide A (**11**) and quinolinone alkaloids **12**–**17** isolated from marine origin.

The bromotyrosine-derived pyrrole alkaloid pseudocerolide A (**11**), was isolated from a marine sponge (*Pseudoceratina* sp.) from the South China Sea in 2020 and its proposed structure could be confirmed by X-ray crystallography (Figure 2). Unfortunately, compound **11** exhibited no activities against methicillin-resistant *Staphylococcus aureus*, *Escheriachia coli*, or *Candida albicans* [45].

The unusual pyrrolyl 1-isoquinolone alkaloids **12** and **13** were discovered from a habitat in the South China Sea within a co-culture of two mangrove endophytic fungi (strain No. 1924 and 3893) in 2006 [46]. It took until 2011, when König and co-workers isolated methyl marinamide (**15**) from the marine sponge (*Ircinia variabilis*) and reported a revised structure of **15**, in which the previously assumed 1-isoquinolone of **13** was reassigned as a 4-quinolinone unit on the basis of X-ray crystallography. Unfortunately, **15** showed only weak or no effects in the biological evaluation on cannabinoid receptors [47]. In accordance with the findings of König, Zhu and Chen, chemically modified the previously isolated compound **14** in 2013, which also led to the revision of the structure **12** to **14** for marinamide in the same fashion, further confirming the revision of marinamide by König and co-workers [48]. However, one year before the report of König, the Lin laboratory isolated the same compound **14**, but referred to it as penicinoline (Figure 2) [49]. Both compounds **14** and **15** display promising in vitro cytotoxicity towards 95-D and HepG2 cell lines (IC_50_ values of 0.57 μg/mL and 6.5 μg/mL, respectively) as well as insecticidal activity against *Aphis gossypii* (100% mortality at 1000 ppm) [48,49].

The related congener penicinoline E (**16**) was isolated from an endophytic fungus *Penicillium* sp. ghq208 in 2012 alongside quinolactacide (**17**), which was isolated from a marine source for the first time [50,51]. In biological assays, moderate cytotoxicity against HepG2 was exclusively attributed to 4-quinolinones **14** and **15** (IC_50_ values of 11.3 μg/mL and 13.2 μg/mL, respectively), indicating the importance of the free carboxy function at C3 (Figure 2) [51].

**Scheme 2:**
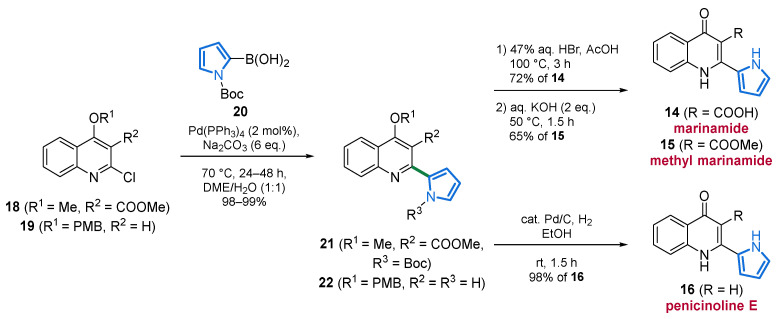
A high-yield sequence towards pyrrolyl 4-quinolinones **14**, **15**, and **16** starting from 2-chloroquinoline precursors **18** and **19** by Nagarajan et al.

Based on the auspicious pharmacological activities of penicinoline E (**16**), marinamide (**14**), and methyl marinamide (**15**), the Nagarajan group established their total synthesis in 2017 for further biological testing [52]. They achieved a two- to three-step approach, characterized by a Suzuki–Miyaura coupling and subsequent dearomatization as key steps from their starting materials **18**, **19**, and **20**. They were also able to unambiguously confirm the structure of penicinoline E (**16**) by X-ray crystallography (Scheme 2) [52].

Furthermore, the antimalarial properties against the 3D7 strain of *Plasmodium falciparum* were evaluated and the decarboxylated derivative **16**, as well as the methyl ester **15**, showed significant activity (IC_50_ value of 1.56 µM for both). These results have been confirmed by binding mode studies of the synthesized ligands **14**, **15**, and **16** to the CYTB protein of *Plasmodium falciparum* [52].

**Figure 3:**
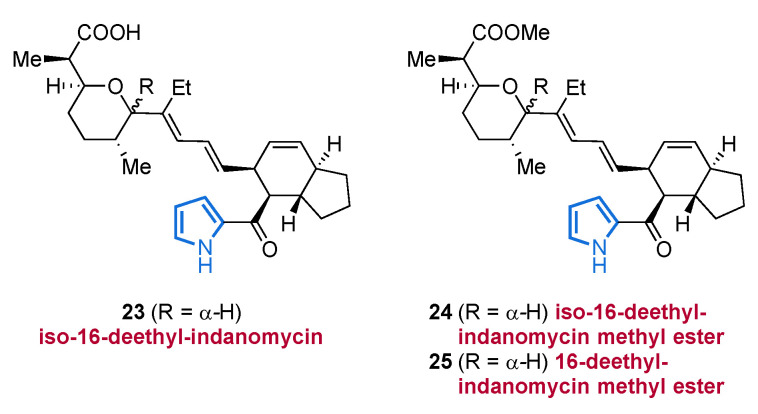
Three new members **23**–**25** of the indanomycin-group, discovered in 2013.

Another pharmacologically interesting compound class is the indanomycins, which possess a variety of biological activities such as antibacterial [53], insecticidal [54], and antiprotozoal [55] properties. In 2011, the group of Kelly and co-workers published a study on the biosynthesis of indanomyincs, including an intramolecular Diels–Alder cyclization of a tetraene as the key step [56]. Two years later, researchers isolated three new representatives of these pyrrole ethers from the culture broth of a marine *Streptomyces anibioticus* strain PTZ0016 which possess in vitro activity against *Staphylococccus aureus* (MIC values between 4.0 and 8.0 µg/mL). Based on their previous derivatives and on the α- or β-orientation of the pyran ring, they were named 16-deethylindanomycins. The relative and absolute configurations of iso-16-deethylindanomycin (**23**), iso-16-deethylindanomycin methyl ester (**24**), and 16-deethylindanomycin methyl ester (**25**) were established by extensive NMR and CD spectroscopy (Figure 3) [57].

Another important source of bioactive MNPs is represented by the genus *Agelas* (family Agelasidae), which provides a wide diversity of glycolipids [58,59], diterpene alkaloids [60,61,62], and pyrrole alkaloids [63,64,65,66]. To date, more than 130 pyrrole alkaloids have been isolated from over 20 *Agelas* species, all of which share a unique bromo- or debromopyrrole-2-carboxamide moiety alongside several linear side chains, anellated ring systems, or dimeric structural units [67].

**Figure 4:**
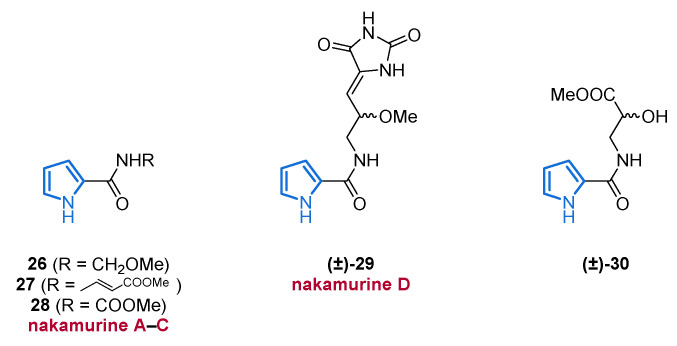
Isolation of five pyrrole-2-carboxamides (**26**–**30**) from the sea sponge *Agelas nakamurai*.

In 2017, Li et al. reported the isolation of the nakamurines A–C (**26**–**28**) from the South China Sea sponge *Agelas nakamurai*. They only differ in the side chain of the carboxamide unit, however, no activity could be observed for any of the compounds in cytotoxicity tests and antiviral assays. In antimicrobial assays, only nakamurine B (**27**) showed weak inhibitory effects against *Candida albicans* (MIC = 60 µg/mL, Figure 4) [67].

A few weeks later, the same group published the extraction of two non-brominated pyrroles, **29** and **30**, from the same sponge *Agelas nakamurai* [68]. For structure elucidation, the racemic pairs were resolved by chiral HPLC with the absolute stereochemistries determined by quantum chemical calculations and measurements of molar rotations. The carboxamide **30** was listed in *SciFinder Scholar* with no associated reference at that time, but the analytical data were reported for the first time. In cytotoxicity and antimicrobial tests, no activity could be observed for any of the enantiomers of nakamurine D (**29**) or for compound **30** (Figure 4) [68].

**Figure 5:**
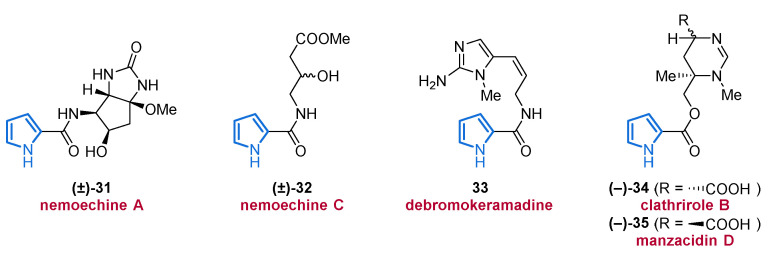
Isolation of nemoechine A (**31**) and C (**32**), debromokeramadine (**33**), and clathrirole B (**34**).

In 2017, Li and co-workers were able to isolate a new class of racemic pyrroles, the nemoechines A–C (**31**, **32**, and **124**), from the species *Agelas* aff. *nemoechinata* (Figure 5) [69]. Nemoechine A (**31**) differs from the two related congeners **32** and **124** by its unusual bicyclic cyclopentane-fused imidazole skeleton, whereas nemoechine B (**124**) features a fused pyrrole core and is therefore specified in Section 2.4. Nemoechine C (**32**), with its butyric acid ester side chain, shows structural similarity to pyrrole **30** and differs only by an additional methylene group. Unfortunately, nemoechine A (**31**) and C (**32**) did not show any promising activities which complies with the inactivity of the structurally related pyrroles **29** and **30** [69].

The isolation of pyrrole-2-aminoimidazole (P-2-AI) debromokeramadine (**33**) from the marine sponge *Agelas* cf. *mauritiana* was reported alongside the first total syntheses of **33** and keramadine (**41**) in 2015. Interestingly, **33** and the previously isolated derivative keramadine (**41**), feature a (*Z*)-configuration at the C=C double bond, which is in contrast to the well-known natural key-precursor oroidin featuring an (*E*)-configured double bond (Figure 5) [70,71].

Clathrirole B (**34**), extracted from the marine sponge *Clathria prolifera*, represents another P-2-AI alkaloid. The carboxylic acid ester **34** is a C-11 epimer of manzacidin D (**35**), which was isolated from the marine sponge *Astrosclera willeyana* back in 1997 (Figure 5) [72]. Interestingly, compound **34** completely lacks antifungal activity against *Saccharomyces cerevisiae*, whereas diastereomer **35** and derivatives thereof proved to be potent antifungals against this yeast [35]. Thus, the authors concluded that the absolute configurations at both C-9 and C-11 may have a massive influence on the antifungal activity of this compound class [73].

**Scheme 3:**
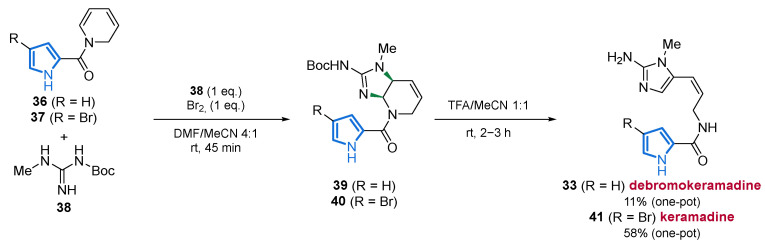
Synthesis of keramadines **33** and **41**, including a regioselective oxidative addition followed by acid mediated bond cleavage of the aminal.

The authors applied a one-pot approach with a regioselective oxidative addition in which partially brominated *N*-acylpyrrole-1,2-dihydropyridines **36** and **37** were reacted with guanidine **38** in a double nucleophilic substitution to generate the aminoimidazoline moiety. Finally, the cyclic aminal structure is ring-opened by TFA, resulting in the MNPs **33** and **41** (Scheme 3) [71].

**Figure 6:**
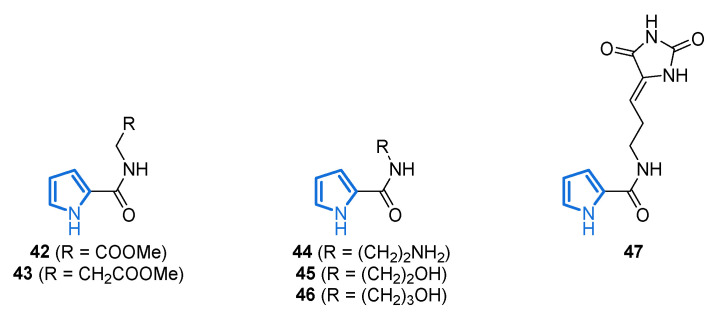
Synthetically known pyrrole-2-carboxamides **42**–**47**, isolated for the first time from marine origin.

In the previously reported isolation of MNPs from *Agelas* aff. *nemoechinata* and *nakamurai*, the class of nakamurines and nemoechines were presented [68,69]. It should be mentioned that the group of Li isolated several structurally related pyrrole alkaloids from marine sources and identified them as known compounds that had been synthesized but not isolated from natural sources before. Therefore, carboxamides **42**–**47**, isolated from marine sources for the first time, are grouped together in Figure 6. The *N*-acylglycine methyl ester **42** identified in both sponges is related to nakamurine C (**28**) but carries an additional methylene group [68,69]. The synthetically known pyrrole **43** bearing two more methylene groups in the side chain, was isolated from *Agelas* *nakamurai* [68,74,75].

Some reduction products of the methyl esters and an amine derivative are represented by compounds **44**–**46**, of which **45** occurs in both sponges, whereas **44** and **46** were exclusively isolated from the *Nemoechinata* sp. [68,69,76,77]. The carboxamide **47** is a debromo analog of mukanadin B and is present in *Agelas nakamurai* [68,78,79]. Compounds **42**–**47** described show neither cytotoxicity nor antimicrobial activity.

**Figure 7:**
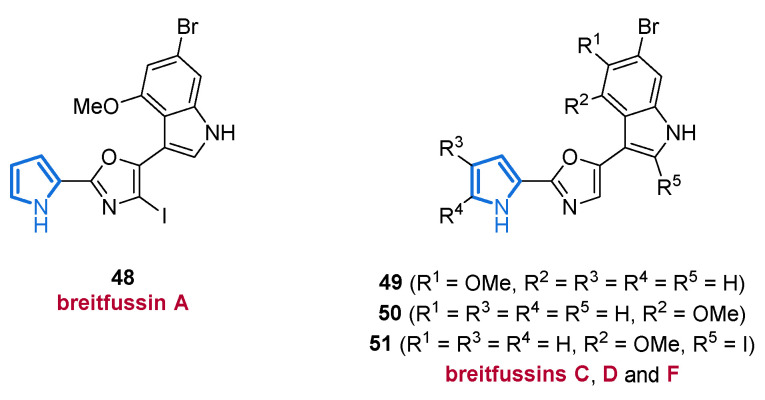
Molecular structures of breitfussins **48**–**51** isolated from the marine hydrozoan *Thuiaria breitfussi*.

The Arctic hydrozoan *Thuiaria breitfussi* (family Sertulariidae) produces a class of indole-oxazole-pyrrole MNPs named breitfussins. Biosynthetically, the breitfussins may share a similar biogenesis as the phorbazoles (cf. Figure 33), arising from the dipeptides Pro-Trp or Pro-Tyr. In the first isolation and analysis of breitfussin A (**48**) in 2012, high-resolution mass spectrometry indicated a ratio of non-hydrogen atoms to hydrogen of 2:1 which makes the structural elucidation by spectroscopic methods challenging [80]. The authors, however, could identify a brominated 4-methoxyindole moiety, a 2-substituted pyrrole core as well as an unresolved C_3_NO fragment suggestive of an oxazole core, which finally prevented the unambiguous determination of the entire structure. By applying a combined approach of atomic force microscopy (AFM), computer-aided structure elucidation (CASE) and calculation of ^13^C-NMR shifts through density functional theory (DFT), the structure of breitfussin A (**48**) could be unequivocally determined (Figure 7) [80]. A recently published article describes the isolation of further non-halogenated congeners, namely breitfussins C (**49**), D (**50**), and F (**51**), of which structures **49** and **50** could also be confirmed by total syntheses (Figure 7) [81].

Given the promising cytotoxic activities of the breitfussins C (**49**) and D (**50**) against several cancer cell lines with IC_50_ values below 10 µM, extensive research on the breitfussin scaffold in search for selective kinase inhibitors has been performed [81]. Due to their promising bioactivity but extremely challenging heteroaromatic core in terms of structure elucidation, the breitfussins are attractive starting points for ongoing synthetic work [82].

**Scheme 4:**
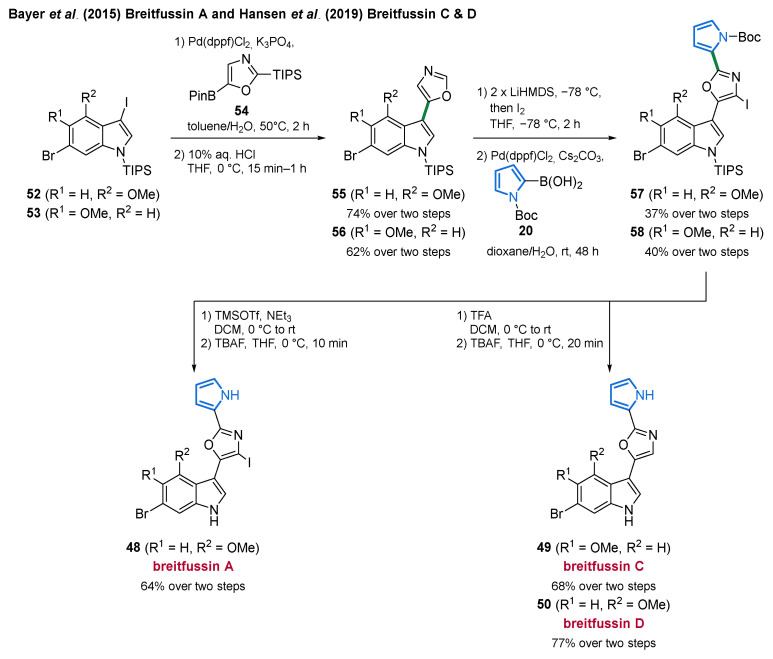
Total synthesis of the three breitfussins A (**48**), C (**49**), and D (**50**) by introducing the oxazole and pyrrole functionalities via two consecutive Suzuki coupling reactions.

The first total synthesis and hence the structure validation of breitfussin A (**48**) was published by the Bayer group in 2015 [83]. They used an approach involving two Suzuki couplings in which the oxazole and pyrrole moieties were installed sequentially. First, indole **52** was converted with oxazole **54** into coupling product **55**, followed by double lithiation of the oxazole core. Coupling with *N*-Boc-2-pyrrole boronic acid (**20**) furnished pyrrole **57**, which, after removal of all protection groups, resulted in the formation of breitfussin A (**48**) [83]. Alongside the isolation of additional breitfussins in 2019, the Bayer laboratory employed the same approach as in their previous publication for the synthesis of breitfussin C (**49**) and D (**50**). Here, only the penultimate step varied by acid-mediated Boc-deprotection, since deiodination of the oxazole core was required (Scheme 4) [81].

**Figure 8:**
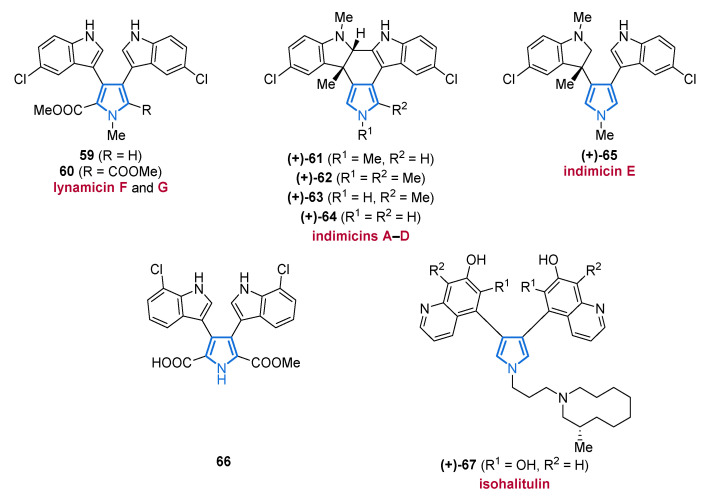
Structures of lynamicins F (**59**) and G (**60**), indimicins A–E (**61**–**65**), dichlorochromopyrrolic acid derivative **66**, and isohalitulin (**67**).

**Scheme 5:**
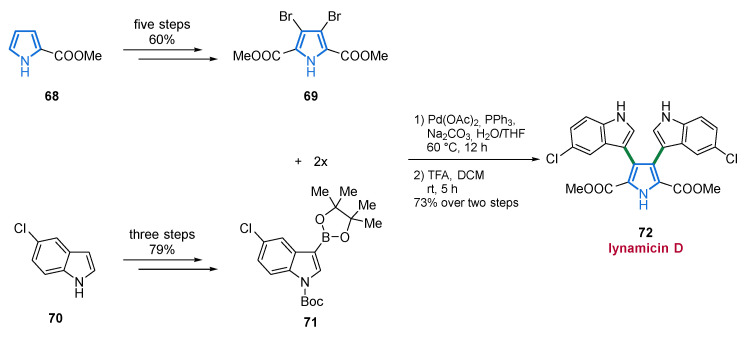
Key step of the synthesis of lynamicin D (**72**) by a Suzuki coupling.

Bisindole pyrroles represent a class of MNPs having similar biological activities. The lynamicins F (**59**) and G (**60**) were isolated from a marine-derived *Streptomyces* sp. SCSIO 03032 [84], extending the lynamicin family, of which lynamicins A–E have been isolated back in 2008 (Figure 8) [85]. Unfortunately, no antimicrobial or cytotoxic activities were observed for **59** and **60** against several indicator strains or cancer cell lines. In 2017, the first total synthesis of the antimicrobial lynamicin D (**72**) was achieved, thereby enabling the implementation of further biological assays (Scheme 5). It turned out that lynamicin D (**72**) influenced the splicing of pre-mRNAs by upregulating the level of the key kinase SRPK1, which is involved in both constitutive and alternative splicing [86].

In addition to the alkaloids **59** and **60**, a new family of MNPs consisting of a unique 1,3-dimethyl-2-hydroindole motif, the indimicins (IDMs) A–E (**61**–**65**), were discovered in 2015 (Figure 8) [84]. Besides the usual spectroscopic data, an X-ray structure of indimicin A (**61**) could be obtained, which allowed determining the absolute configuration of the hydroindole moiety. Of compounds **61**–**65**, only indimicin B (**62**) was active against the breast cancer cell line MCF-7 (IC_50_ value of 10.0 µM ± 0.3 µM), whereas all seven alkaloids **61**–**65** did not show any antimicrobial or cytotoxic activities against several indicator strains or cancer cell lines [84].

Very recently, the *Streptomyces* sp. SCSIO 11791 revealed another bisindolylpyrrole (**66**)**,** displaying moderate cytotoxicity against a human breast cancer cell line (MDA-MB-435, IC_50_ value of 19.4 µM), while no antibacterial properties could be observed (Figure 8) [87].

In isohalitulin (**67**), isolated from the marine sponge *haliclona tulearensis* in 2010, the structure is dominated by a bis-dihydroxyquinoline functionality (Figure 8) [88]. Compound **67** exhibits a detectable toxicity to brine shrimp (*Artemia salina*, LD_50_ value of 0.9 mM). It is also worth mentioning that minute amounts and instability of isohalitulin (**67**) prevented the unequivocal determination of its structure. However, **67** shows very similar analytical data to its congener halitulin and should differ only in the position of the two phenolic OH groups (Figure 8). Although no experiments were performed to deduce the stereochemistry of **67**, the authors mentioned that, on the grounds of common biogenetic precursors, it most probably has the same absolute configuration as halitulin [88].

The total synthesis of lynamicin D (**72**) commenced with the synthesis of the coupling partners **69** and **71**, prepared from commercially available precursors **68** and **70**. Dibrominated pyrrole **69** was obtained by a Vilsmeier–Haack reaction, followed by oxidation, esterification, and final bromination. On the other side, 5-chloro-1*H*-indole (**70**) was first iodinated and Boc-protected and the introduction of the pinacol moiety on the basis of Pd-catalysis resulted in the formation of indole precursor **71**. Building blocks **69** and **71** were then subjected to the key Suzuki coupling. Final removal of the Boc-group gave lynamicin D (**72**) in 73% yield over two steps (Scheme 5) [86].

**Figure 9:**
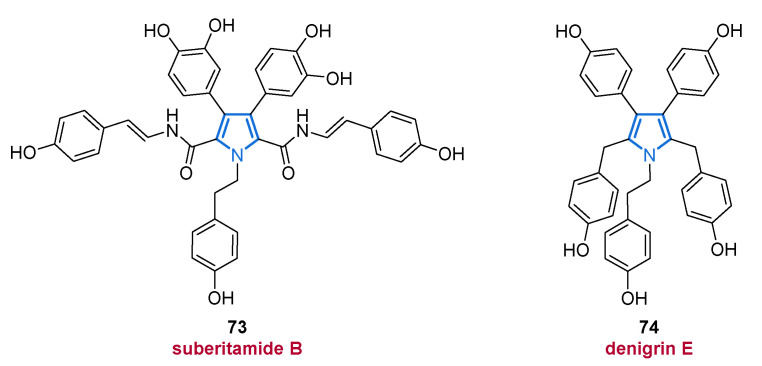
Highly substituted 3,4-diarrylpyrroles suberitamide B (**73**) and denigrin E (**74**).

The suberitamides and denigrins constitute another family of highly substituted pyrrole alkaloids. The symmetrical, nearly planar suberitamide B (**73**) was isolated from the marine sponge *Pseudosuberties* sp. in 2020 and bears a fully substituted pyrrole core. This storniamide-related compound inhibits the enzymatic activity of Cb1-b (E3 ubiquitin ligase) with an IC_50_ value of 11 µM, which, according to the authors, is caused by the rigid, highly substituted pyrrole scaffold (Figure 9) [89].

In 2020, denigrin E (**74**) was isolated from a new *Dactylia* sp. along with several members of the pyrrolone family. Unfortunately, no inhibitory activity against PAX3-FOXO1 luciferase expression was observed in biological assays (Figure 9) [90]. By considering the substitution pattern of these 3,4-diarylpyrroles **73** and **74**, a close relationship as potential precursors of lamellarins (see Section 2.4.1) in a biosynthetic context can be suggested.

**Figure 10:**
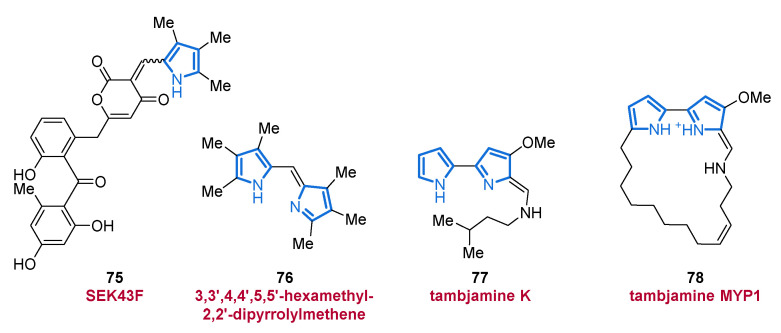
Representation of an APK (**75**) and three pyrroles **76**–**78** including the important class of tambjamines.

Among the huge variety of marine alkaloids, aromatic polyketides (APK) represent another large class of MNPs and pyrrole-containing representatives have been described. The group of Zhang and co-workers isolated the decaketide pyrrole SEK43F (**75**) generated from pathway crosstalk of the host *Streptomyces albus* J1074 and the heterologous fls-gene cluster from *Micromonospora rosaria* SCSIO N160 (Figure 10) [91]. It should be mentioned that the configuration of the double bond in **75** could not be unequivocally determined. The same group also isolated another tri-methylated bis-pyrrole **76** (Figure 10) [91], which has only been known as a synthetic product before [92,93]. Both compounds **75** and **76** displayed negligible antibacterial activity, whereas the APK **75** showed weak to moderate cytotoxicity against four human cancer cell lines (SF-268, MCF-7, NCI-H460, and HePG-2, with IC_50_ values of 56.46 µM ± 0.87 µM, 35.73 µM ± 1.45 µM, 44.62 µM ± 2.49 µM, and 39.22 µM ± 3.00 µM, respectively, Figure 10).

The family of tambjamines consisting of a central bi-pyrrole unit is counted among the 4-methoxypyrrolic natural products. In 2010, tambjamine K (**77**) was isolated as the main secondary metabolite from the Azorean nudibranch mollusk *Tambja ceutae* and in minute amounts from the bryozoan *Bugula dentata* (Figure 10) [94]. Just as its family members, tambjamine K (**77**) exhibited remarkable to moderate antiproliferative activity against tumor and non-tumor mammalian cells with IC_50_ values between 3.5 nM and 19 µM. It is suspected that the strong activity is caused by the bipyrrolic structure with its DNA-targeting properties and by the ability to form ion complexes [94].

The macrocyclic tambjamine MYP1 (**78**) is produced by the marine bacterium *Pseudoalteromonas citrea* and was isolated in 2019 (Figure 10) [95]. The authors highlighted the important differences of the α- and β-rotamers in the tambjamine conformations, which are thought to play an essential role in their bioactivity. Moreover, the group provides an X-ray structure by co-crystallization of **78** with formic acid, unequivocally confirming the proposed structure of compound **78** [95].

**Scheme 6:**
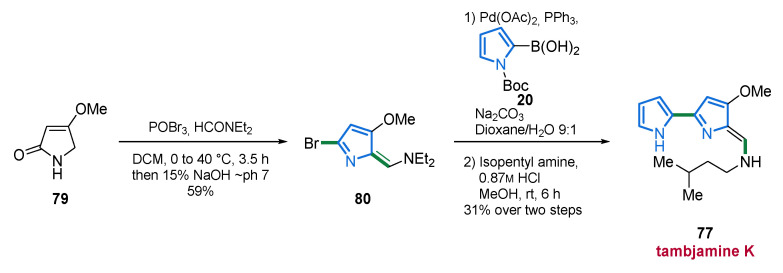
A linear 3-step sequence to tambjamine K (**77**).

Based on the promising bioactivity of compound **77**, Lindsley et al. were prompted to publish their first three-step total synthesis of tambjamine K (**77**) four months after its initial isolation [96]. The first step involved a Vilsmeier–Haack haloformylation which generated enamine **80** in 59% yield. A Suzuki coupling with Boc-1*H*-pyrrol-2-ylboronic acid (**20**) followed by acid-mediated condensation of isopentylamine resulted in the formation of tambjamine K (**77**) in 31% over two steps (Scheme 6) [96]. In addition to the natural product synthesis, a series of unnatural derivatives were synthesized followed by biological assays to evaluate basic structure–activity relationships (SAR). However, the natural product **77** showed moderate activity (IC_50_ values of 13.7 µM and 15.3 µM against HCT116 and MBA231, respectively), whereas the unnatural analogs were more potent in inhibiting the viability, proliferation, and invasion of HCT116, MBA231, SW 620, and H520 NSCLC cancer cell lines (IC_50_ values between 146 nM and 10 µM) [96].

**Figure 11:**
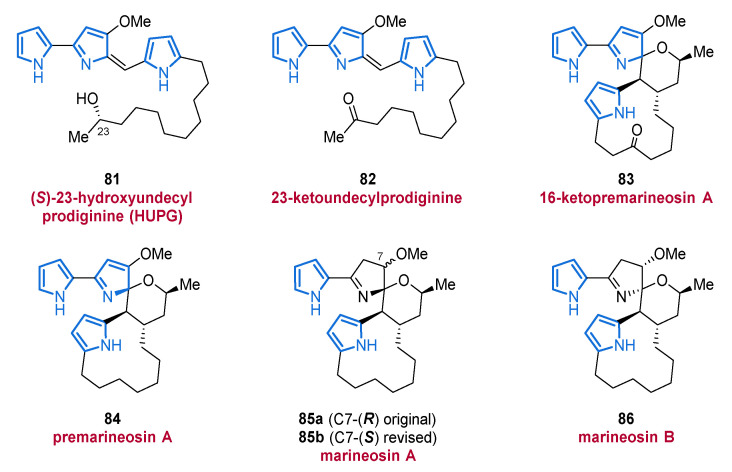
Different prodiginine-based pyrrole alkaloids **81** and **82** together with marineosin-type spiroaminals **83**–**86**.

In addition to the tambjamines which consist of a bipyrrole core functionalized with various imines, the functionalization with an additional pyrrole moiety in the prodiginine structures represents another well-studied family. With the isolation of the marineosins A (**85a**) and B (**86**) in 2008, this prodiginine-related family opened up a new field of research with several new contributions being made in the last decade [97]. In 2014, the Reynolds laboratory focused on the final steps of the marineosin biosynthesis, by exploring the biosynthetic gene cluster *mar* which can produce marineosins by a heterologous expression in a *Streptomyces venezuelae* derived JND2 strain. They replaced the *marA* and *marG* gene with the spectinomycin resistance *aadA* gene which led to the isolation and elucidation of 16-ketopremarineosin A (**83**) and premarineosin A (**84**) as well as 23-hydroxyundecylprodiginine (HUPG) (**81**) and its oxidized derivative **82**, respectively (Figure 11). As marineosin production was not observed, the authors concluded that both genes, *marA* and *marG*, are essential for the biosynthesis of marineosins [98]. Three years later, the Reynolds group reported another gene (*marH*) from the same cluster which has the ability to catalyze the condensation of a methoxybipyrrole carbaldehyde (MBC) and 2-undecylpyrrole (UP) to generate undecylprodiginine (UPG). The gene also hydroxylates the C-23 position of UPG to construct HUPG (**81**) and hence is essential for the biosynthetic pathway of marineosins [99].

**Scheme 7:**
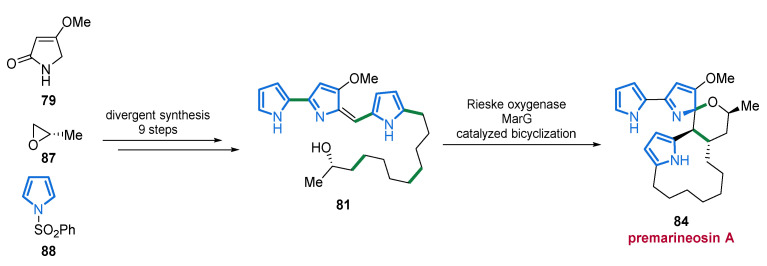
Divergent synthesis of premarineosin A (**84**) including a bioinspired MarG catalyzed spirocyclization as the final step.

Not only the biosynthetic pathway but also the stereoselective synthesis of marineosins, their substructures, and derivatives have attracted much attention. In 2014, the Reynolds laboratory followed up on their previous publications regarding marineosins and reported the first total synthesis of HUPG (**81**) and premarineosin A (**84**). To this end, a divergent synthetic approach of nine steps in total stereospecifically provided 23-hydroxyundecylprodiginine (**81**). The final cyclization forming the spiro-tetrahydropyran-aminal unit of the premarineosin A (**84**) was then achieved by a biosynthetic approach via the Rieske oxygenase MarG (Scheme 7) [100]. This strategy yields several other prodiginine derivatives and premarineosin analogs that show promising cytotoxic and antimalarial activities [100].

**Scheme 8:**
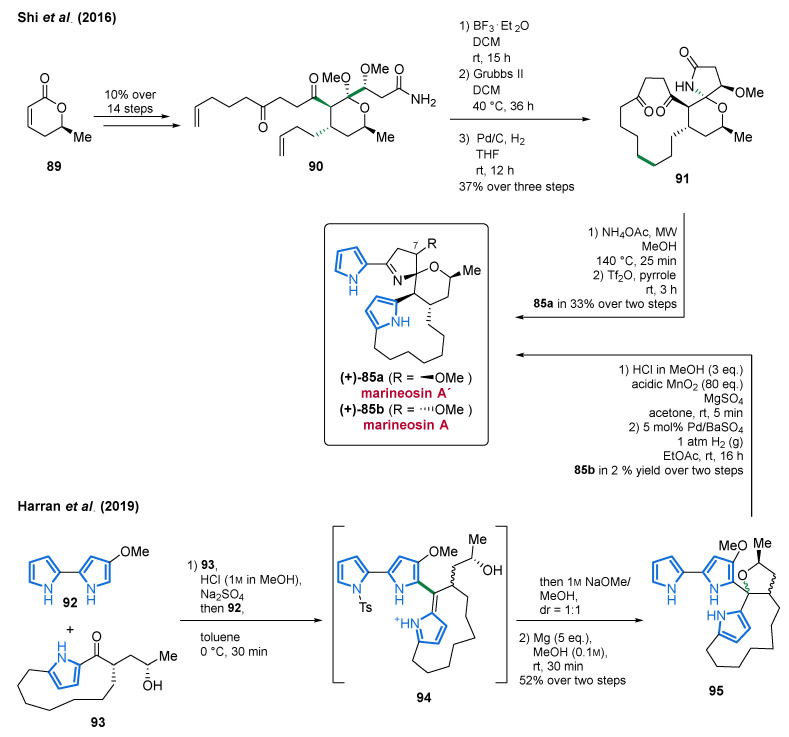
The first total synthesis of 7-*epi*-marineosin A (**85a**) by Shi and co-workers in a linear 19 step sequence and the structural reassignment of C7-OMe from (*R*) to (*S*) by the Harran laboratory using a chromophore disruption approach.

Based on unsuccessful synthetic attempts (with the exception of individual key motifs) of several research groups [101,102,103,104,105,106], Shi and co-workers presented the first total synthesis of marineosin A (**85a**) in 2016 [107]. The synthesis commenced with the commercially available (S)-pyrone **89**, which was converted into key fragment **90** in 10% yield over 14 steps. Lewis acid-mediated spirocyclization and ring-closing metathesis followed by hydrogenation furnished spiro lactam **91** in 37% yield over three steps. The last two steps consisted of a Paal–Knorr reaction and a Vilsmeier–Haack reaction, not only allowing for the preparation of the sensitive pyrrole moieties in a late-stage procedure but also directly giving access to marineosin A′ (Scheme 8). It is also worth mentioning that five X-ray structures of important intermediates could be obtained, underpinning the validity of the synthesis. However, the NMR spectra, appearance, and optical rotation of the resulting marineosin A′ (**85a**) exhibited some deviations when compared to the isolated natural product, suggesting that the natural and synthetic compounds likely differ in their stereochemistry [107].

It was however not until 2019, that the Harran group solved the puzzle by a total synthesis and concomitant reassignment of C7-(*R*) in **85a** to C7-(*S*) resulting in the structure **85b** for (+)-marineosin A [108]. To this end, a bioinspired approach with reversed fragment polarity was applied, starting from the previously prepared bipyrrole **92** and cyclic ketone **93**. Condensation product **94** was stabilized by quenching with NaOMe, generating a novel but still unstable premarineosin **95**. After exposure to acidic conditions, a prodiginine chromophore was formed, which, after 6-exo trig cyclization mediated by acidic MnO_2_, was converted to a premarineosin derivative. The formed vinylogous imidate was hydrogenated from the less hindered face, resulting in the formation of (+)-marineosin A (**85b**), whose spectroscopic data are in full agreement with those reported for the isolated natural product **85b** (Scheme 8) [108].

### 2.2. Formylpyrroles

In addition to the acyl-, carboxy-, and carboxamido-pyrroles (**1**–**3**, **23**–**25** and **26**–**34**) shown in the previous Section (cf. Section 2.1), the formylpyrroles constitute another distinct family of the marine pyrrole alkaloids [109].

**Figure 12:**
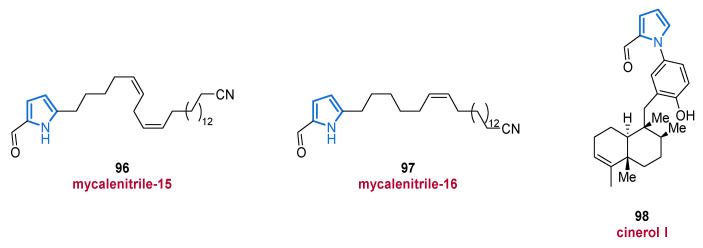
Mycalenitrile **96** and **97** as well as the pyrrole-terpenoid **98**.

In the course of an investigation of the South China Sea sponge *Mycale lissochela* in 2017, two new formylpyrroles **96** and **97** bearing an aliphatic side chain with a terminal nitrile group were isolated (Figure 12) [110]. Both mycalenitrile-15 (**96**) and mycalenitrile-16 (**97**) showed excellent and good inhibition effects against PTP1B (protein-tyrosine phosphatase 1B, a recognized target for diabetes and obesity) with IC_50_ values of 8.6 µmol/L and 3.1 µmol/L, respectively, resulting from the unsaturated side chain [110].

An additional formylpyrrole, cinerol I (**98**), was isolated from the sponge *Dysidea cinerea* and belongs to the meroterpenoid family (Figure 12) [111]. Cinerol I (**98**), which lacks the unsaturated side chain present in compounds **96** and **97**, showed no inhibitory activity against PTP1B, ATP-citrate lyase (ACL), or SH2 domain-containing phosphatase-1 (SHP-1) [111].

**Figure 13:**
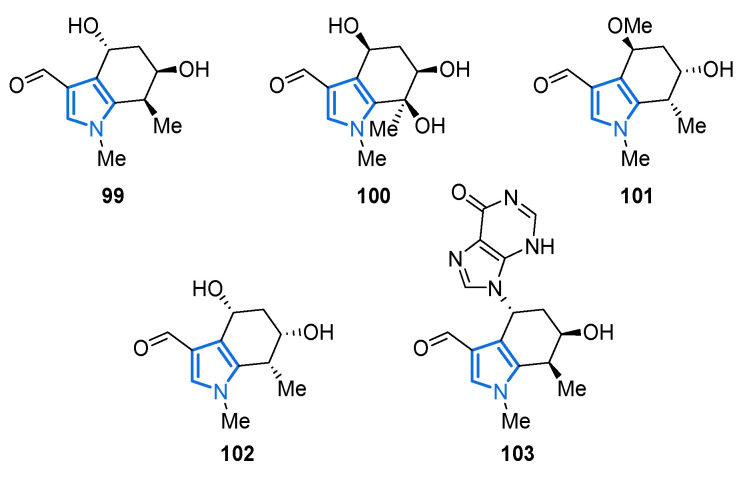
Representation of five tetrahydroindoles **99**–**103** isolated from *Moorea producens*.

Five new formylpyrroles **99**–**103** were isolated from the marine cyanobacterium *Moorea producens* in 2017 (Figure 13) [112]. Biosynthetically, they are suggested to originate from the amino acid tryptophan, the indole moiety of which is partly reduced to forge the annellated tetramethylenepyrrole framework. Further annellated pyrroles are depicted in Section 2.4. All pyrroles described herein feature a 3-formyl group, and compound **103** additionally carries a purine unit. The five isolated pyrroles **99**–**103** showed no noteworthy cytotoxicity or antibacterial properties [112].

### 2.3. Nitropyrroles

A new subclass of pyrroleterpene MNPs is represented by 2-nitro-substituted pyrroles carrying a diversely functionalized farnesyl chain attached to the 4-position of the pyrrole core. The nitropyrrolin and heronapyrrole families known to date are formed biosynthetically by means of an electrophilic aromatic substitution of the pyrrole core by a farnesyl pyrophosphate. Subsequent nitration, oxidation to epoxides and alcohols, as well as cascade cyclization reactions then produce a variety of different substituted metabolites.

**Figure 14:**
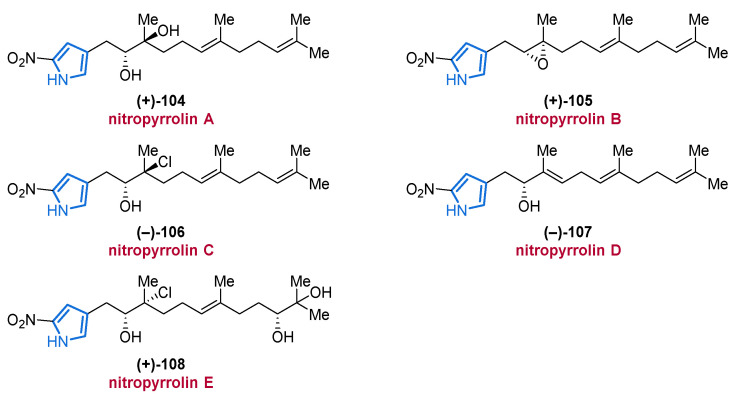
Nitropyrrolins A–E (**104**–**108**) represent the family of 4-farnesylated 2-nitropyrroles.

The first MNP from this subclass was isolated back in 2006, however, the structural characterization appears to be incomplete and no information about the stereochemistry was given [113]. In 2010, the group of Fenical reported the isolation of five farnesyl-2-nitropyrroles **104**–**108** from the marine actinomycete strain CNQ-509 and referred to them as nitropyrrolins A–E (**104**–**108**) (Figure 14) [114]. The authors performed several chemical modifications, including an acetonide formation from epoxide **105**, and the Mosher method was applied to unequivocally identify the full stereochemistry of nitropyrrolins A–E (**104**–**108**). Among compounds **104**–**108**, nitropyrrolin D (**107**) displayed the most promising IC_50_ value of 5.7 µM in biological assays against HCT-116 colon carcinoma cells, whereas a lower antibacterial activity against MRSA was observed for all nitropyrrolins **104**–**108** (MIC values >20 µg/mL). Some of the synthetic derivatives synthesized in the course of the structure elucidation process showed strong to moderate cytotoxic (IC_50_ values between 9.2 µM and 24.4 µM) and promising antibacterial properties (MIC value of 2.8 µg/mL) [114].

**Scheme 9:**
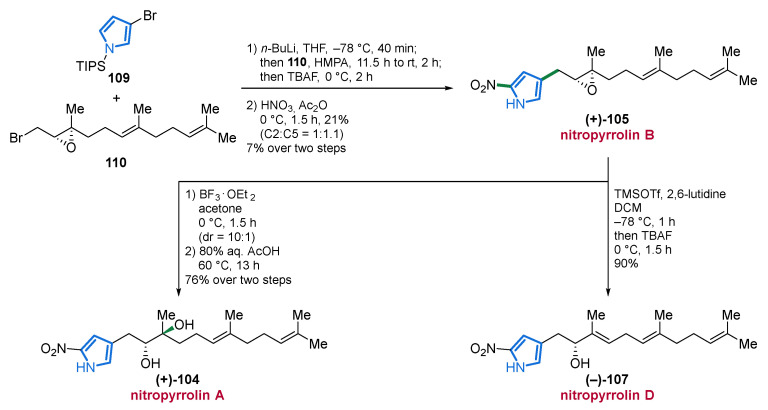
Total synthesis of nitropyrrolins **104** and **105** via the key intermediate nitropyrrolin B (**105**) that is also suggested to be a biosynthetic precursor of nitropyrrolins A (**104**) and D (**107**).

In 2016, the Morimoto group reported the first total synthesis of nitropyrrolins A (**104**), B (**105**), and D (**107**) in a sequential fashion (Scheme 9) [115]. As a key step, the authors performed a lithium–halogen exchange on bromopyrrole **109** and reacted the intermediary lithium species with epoxybromide **110**, which was prepared from a known epoxy alcohol. Subsequent deprotection and α-nitration of the pyrrole core then furnished nitropyrrolin B (**105**) in 7% over two steps. Treatment of the epoxide **105** with BF_3_∙OEt_2_ and acetone produced the *cis*-acetonide, the stereochemistry of which could be investigated by NOE spectroscopy. Cleavage of the acetonide under acidic conditions then generated nitropyrrolin A (**104**) in 76% over two steps. When nitropyrrolin B (**105**) was reacted with TMSOTf, a regio- and stereoselective epoxide ring-opening occurred. In a one-pot approach, the intermediary allylic TMS-ether was cleaved under the addition of TBAF producing nitropyrrolin D (**107**) in 90% yield (Scheme 9) [115].

**Figure 15:**
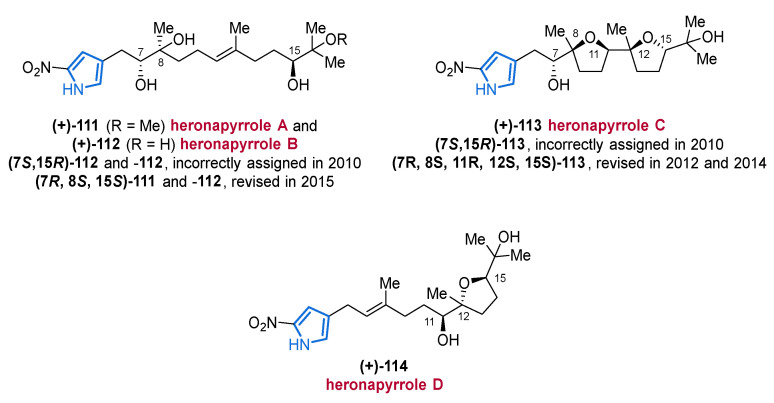
The heronapyrroles A–D (**111**–**114**) only differ in their oxidation state in the farnesyl side chain.

Only a few days after disclosure of nitropyrrolins A–E (**104**–**108**) as natural products, the group of Capon reported the extraction of three further 2-nitropyrroles, the heronapyrroles A–C (**111**–**113**) (Figure 15) [116]. These compounds share the same 4-farnesyl-2-nitropyrrole scaffold and are closely related to the nitropyrrolins **104**–**108** (Figure 14). The heronapyrroles **111**–**113** were isolated from a microbial culture of *Streptomyces* sp. strain CMB-M0423 in only minor quantities, which prevented a meaningful analysis of the full stereochemistries. However, on the basis of biosynthetic considerations, the absolute configurations were tentatively assigned as 7*S* and 15*R*. Although heronapyrroles A–C (**111**–**113**) neither displayed cytotoxicity against several cell lines (HeLa, HT-29, AGS) nor showed any activity towards Gram-negative bacteria such as *Pseudomonas aeruginosa* (ATCC 10145) and *Escherichia coli* (ATCC 11775), promising activity against Gram-positive bacteria such as *Staphylococcus aureus* (ATCC 9144, IC_50_ values between 0.6 µM and 0.8 µM) and *Bacillus subtilis* (ATCC 6633, IC_50_ values between 0.8 µM and 4.2 µM) could be observed [116].

**Scheme 10:**
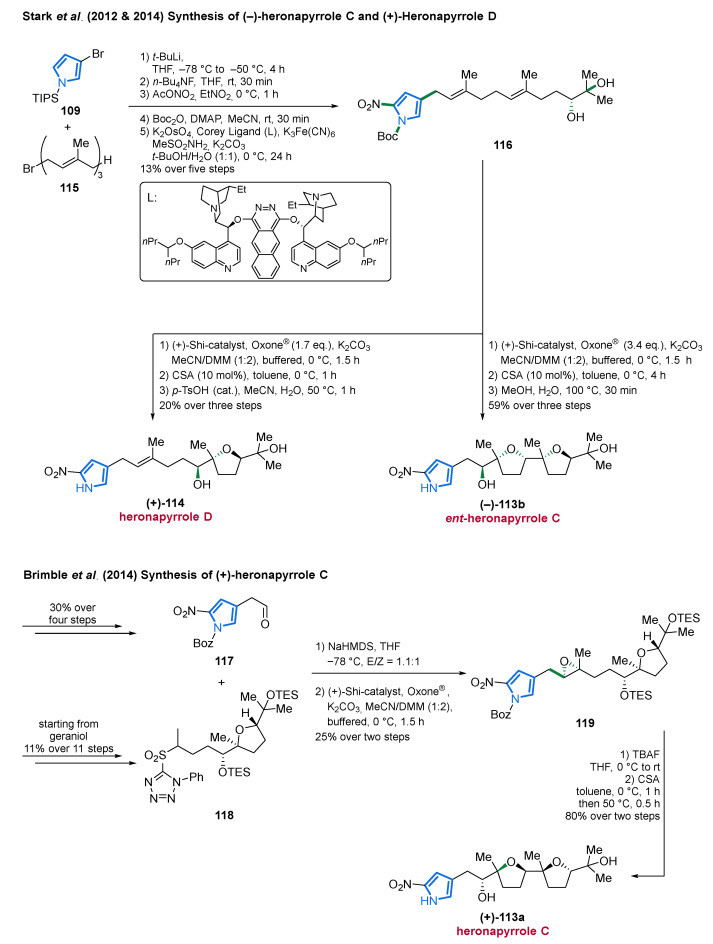
First total synthesis of (+)-heronapyrrole C (**113a**) by Brimble in 2014 and its enantiomer (−)-heronapyrrole C (*ent*-**113b**) by Stark.

Since the stereochemistries of heronapyrroles A–C (**111**–**113**) were only based on a biosynthetic assumption, several total syntheses of members belonging to the heronapyrrole family have been undertaken in the last decade. In 2012, Stark and co-workers focused on biosynthetic considerations and published a bioinspired synthesis attempting to synthesize heronapyrrole C (**113**) [117]. Starting with a lithium–halogen exchange-mediated coupling of 3-bromopyrrole **109** and farnesyl bromide **115** followed by nitration of the pyrrole core and Boc-protection, farnesylpyrrole **116** was generated in 13% over five steps. Asymmetric dihydroxylation of compound **116**, followed by a key double organocatalytic epoxidation using the (+)-Shi catalyst enabled a biomimetic polyepoxide cyclization cascade under acidic conditions, yielding pyrrole *ent*-**113b**. However, the product *ent*-**113b** showed an opposite optical rotation compared to the isolated natural product, prompting the authors to propose the corresponding enantiomer (+)-**113a** to be the true natural structure (Scheme 10) [117].

Just as heronapyrroles A–C (**111**–**113**), heronapyrrole D (**114**) could be isolated by Stark and co-workers from a microbial culture of *Streptomyces* sp. (strain CMB-M0423) in 2014 and showed significant inhibition of Gram-positive bacteria *Staphylococcus aureus* subsp. (ATCC 25923, IC_50_ value 1.8 µM), *Staphylococcus epidermis* (ATCC 12228, IC_50_ value 0.9 µM) and *Bacillus subtilis* (ATCC 6633, IC_50_ value 1.8 µM), but was inactive against Gram-negative bacteria *Pseudomonas aeruginosa* (ATCC 10145), *Escherichia coli* (ATCC 25922) and *Candida albicans* (ATCC 90028) [118]. Along with its isolation, the authors also published the total synthesis of (+)-heronapyrrole D (**114**), using the same strategy as in their previous synthesis of 2012. The only exception is represented by the Shi-epoxidation, in which substoichiometric amounts of the oxidant (Oxone^®^) were applied to generate *mono*-epoxides. Cyclization furnished the desired (+)-heronapyrrole D (**114**) (Scheme 10) [118].

Although the Stark laboratory further elaborated their studies on the nitration step and improved the entire synthesis in 2014 [119], the group of Brimble published the first total synthesis of the naturally occurring (+)-heronapyrrole C (**113a**) almost at the same time [120]. Based on their key intermediates **117** and **118**, synthesized in 4 and 11 steps, respectively, a Julia–Kocienski olefination merged the pyrrole subunit and the terpenoid side chain. A subsequent Shi-epoxidation then furnished compound **119** in 25% over two steps. The authors mentioned that the use of *N*-benzoyloxymethyl (Boz) as a protecting group was crucial to perform the final cyclization and deprotection under mild conditions. In this way, (+)-heronapyrrole C (**113a**) could be obtained in 80% yield over two steps (Scheme 10) [120]. The spectroscopic data of the (+)-isomer **113a** match those of the natural product and confirm the proposed reassignment by Stark et al. in 2012.

**Scheme 11:**
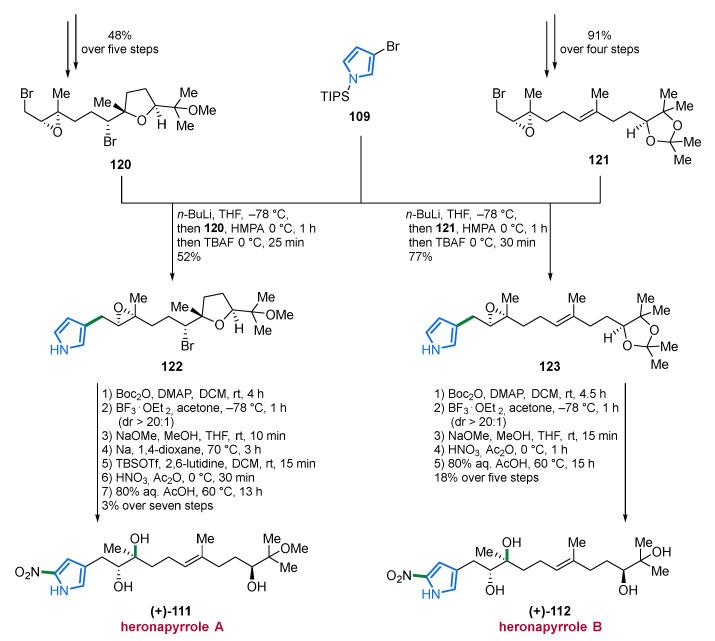
Total synthesis of (+)-heronapyrrole A (**111**) and (+)-heronapyrrole B (**112**) by a convergent approach leading to stereochemical reassignments.

In 2015, the Morimoto group published the total synthesis of the remaining (+)-heronapyrroles A (**111**) and B (**112**) [121]. Taking into account the reported syntheses of (−)-heronapyrrole C (*ent*-**113b**) by Stark (2012) and (+)-heronapyrrole C (**113a**) by Brimble (2014) together with the biogenetic relationship of heronapyrroles A–C (**111**–**113**), a stereochemical reassignment of pyrroles **111** and **112** was proposed. Morimoto’s group established a strategy similar to the approaches published by Stark and Brimble by installing the farnesylated chain through alkylation of pyrrole **109** with epoxy bromides **120** or **121**. In the case of (+)-heronapyrrole A **111**, the generated epoxide **122** was opened regioselectively by BF_3_∙OEt_2_, yielding a masked C7–C8 *anti*-diol, which, after sodium-mediated ring-opening of the THF moiety and several further transformations, led to the formation (+)-heronapyrrole A (**111**) in 3% yield over seven steps (Scheme 11). Just as (+)-**111**, (+)-heronapyrrole B (**112**) was synthesized in a corresponding manner by opening the epoxide **123** via the same sequence to give a *cis*-acetonide, which, after nitration and acid-mediated cleavage of the acetonide functional groups, gave (+)-heronapyrrole B (**112**) in 18% yield over five steps (Scheme 11). In both cases, the absolute configuration was determined by the Mosher method which confirmed the proposed structure. As a consequence, the initially proposed stereochemistries for heronapyrroles A (**111**) and B (**112**) from the Stark laboratory in 2012 were reassigned [121].

This rare class of nitropyrroles has attracted some attention from synthetic chemists in recent years. Not least because of previous synthetic work and the promising effects against Gram-positive bacteria, nitropyrroles may represent interesting targets for further drug design [115,117,118,120,121,122,123].

### 2.4. Annellated Pyrroles

In contrast to simple substituted pyrrole alkaloids, another structural class comprises compounds with an annellated pyrrole core. The position of fusion thereby can differ between 1,2-, 2,3- or 3,4-, with the fused ring being 6- or 7-membered. Additionally, these alkaloids often share a carbonyl moiety in α-position to the bridgehead atom.

**Figure 16:**
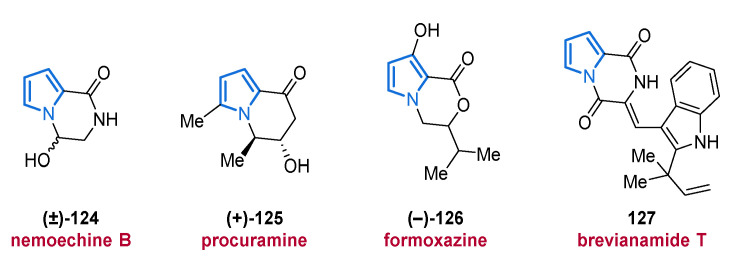
Structures of 1,2-annellated marine pyrrole alkaloids **124**–**127**.

From a series of nemoechines isolated in 2017 (see Figure 5, **31** and **32**), nemoechine B (**124**) stands out with its 1,2-condensed pyrrole unit [69]. The synthetically known compound **124** [124] was originally isolated in racemic form from *Agelas* aff. *nemoechinata* and the enantiomers were separated by chiral HPLC. Like its family members **31** and **32**, a lack of cytotoxicity against HL-60, HeLa, P388, and K562 cell lines was reported for both enantiomers (Figure 16) [69].

In 2016, procuramine (**125**) was identified as a co-metabolite during the initial isolation and investigation of the biosynthetic pathway of curindolizine (**414**) from *Curvularia* sp. IFB-Z10 (see Figure 58). Structure elucidation was performed by spectroscopic methods and X-ray crystallography (Figure 16) [125].

A new pyrrolooxazine (**126**) was isolated from the marine mudflat fungus *Paecilomyces formosus*, yet the absolute configuration could not be determined because of decomposition during the isolation process. Formoxazine (**126**) showed potential as a radical scavenger in the DPPH assay with an IC_50_ value of 0.1 µM and antibacterial activity against MDRSA and MRSA (MIC values of 6.25 µg/mL for both) (Figure 16) [126].

In the course of an investigation of marine-derived *Aspergillus versicolor* and in search for new Bacille Calmette-Guérin-inhibiting antibiotics against tuberculosis, the unknown brevianamide T (**127**) could be isolated in 2012 (Figure 16) [127]. Unfortunately, diketopiperazine **127**, isolated along with other members of the brevianamide family, showed no antibacterial properties against *Staphylococcus aureus* (ATCC 6538), *Bacillus subtilis* (ATCC 6633) (Gram-positive bacteria) or *Pseudomonas aeruginosa* (PAO1), *Escherichia coli* (ATCC 25922) (Gram-negative bacteria) or *Candida albicans* (SC 5314, yeast) [127].

**Figure 17:**
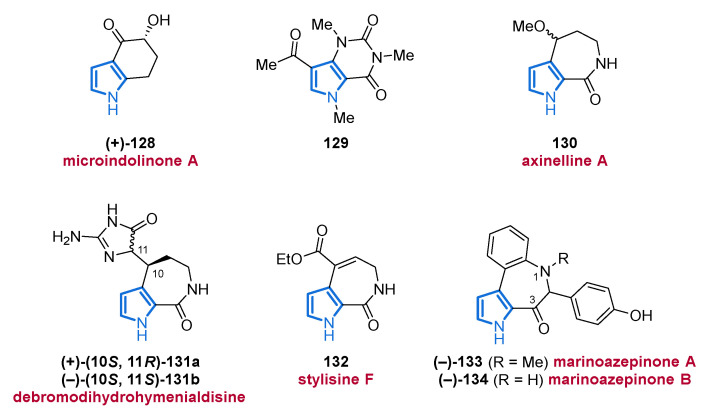
Various 2,3-fused pyrrole alkaloids **128**–**134** isolated between 2010 and 2020.

A 2,3-fused pyrrole alkaloid, microindolinone A (**128**), was isolated from the actinomycete *Microbacterium* sp. MCCC 1A12207 from the deep sea in 2017 [128]. This tetrahydroindole represents one of two known saturated indoles of natural origin [129]. The absolute configuration at C5-OH was deduced with CD spectroscopy as 5*R*. No potent inhibition was found in anti-allergic bioactivity tests against RBL-2H3 cells (Figure 17) [128].

The natural product **129** was isolated from the gorgonian coral *Verrucella umbraculum* in 2012 and features a pyrrolopyrimidin scaffold. According to the authors, the biosynthesis of this purine alkaloid is similar to that of caffeine, which was also isolated from the same source (Figure 17) [130].

Another important class of MNPs is comprised of the pyrrolactams, which most probably derive from pyrrole-2-carboxamides. Axinelline A (**130**) was isolated alongside its brominated analog **353** (see Figure 51) from the marine sponge *Axinella* sp. in 2017, however, the absolute stereochemistry was not determined (Figure 17) [131].

The two diastereomers (11*R*)- and (11*S*)-debromodihydrohymenialdisine **131a** and **131b** were isolated from the sponge *Cymbastela cantharella* by the Debitus laboratory in 2011 (Figure 17) [132]. The authors assumed that compounds **131a** and **131b** biogenetically arise from dispacamide derivates. Because of their close relationship to the strong kinase inhibitor hymenialdisine, (11*R*)- and (11*S*)-debromodihydrohymenialdisine **131a** and **131b** were tested for Polo-Like-Kinase-1 (PLK-1) inhibition. Unfortunately, but in analogy to the bromo derivatives **386a** and **386b** (see Figure 55), a complete lack of activity was observed, demonstrating the importance of the conjugation at C-10 and C-11 of the unique cyclic system of hymenialdisine [132].

In 2018, the structurally related seven-membered pyrroloazepine stylisine F (**132**) was isolated alongside several other MNPs from the marine sponge *Stylissa massa*. However, the authors mentioned that stylisine F (**132**) most probably occurred as an artifact generated from the corresponding acid upon EtOH extraction. In basic biological investigations, weak or no inhibition against a variety of bacteria was detected (MIC ≥ 128 µg/mL, Figure 17) [133].

In 2015, Fenical and co-workers reported a culture-dependent technique in a nutrient-poor medium combined with long incubation times, which facilitated the cultivation of several marine bacteria able to produce secondary metabolites. The organic extract from strain CNX-216^T^ of a cultivated bacterium belonging to the Mooreiaceae family showed activity against *Pontibacillus* sp. and the authors were able to isolate the alkaloids marinoazepinones A (**133**) and B (**134**) from this extract [134]. Besides the incorporation of the unusual amino acid 4-hydroxyphenylglycine, the marinoazepinones **133** and **134** represent the first natural products featuring a rare azepin-3-one framework. CD spectroscopy, X-ray crystallography, and optical rotation were used to elucidate the absolute stereochemistry at C2, but no definite conclusions could be drawn. In bioactivity assays, marinoazepinone B (**134**) exhibited antibacterial activity against the Gram-positive *Pontibacillus* strain CNJ-912 (16 mm inhibition zone), whereas no activity was observed against the Gram-negative *Vibrio shiloi* strain CUA-364 (Figure 17) [134].

**Scheme 12:**
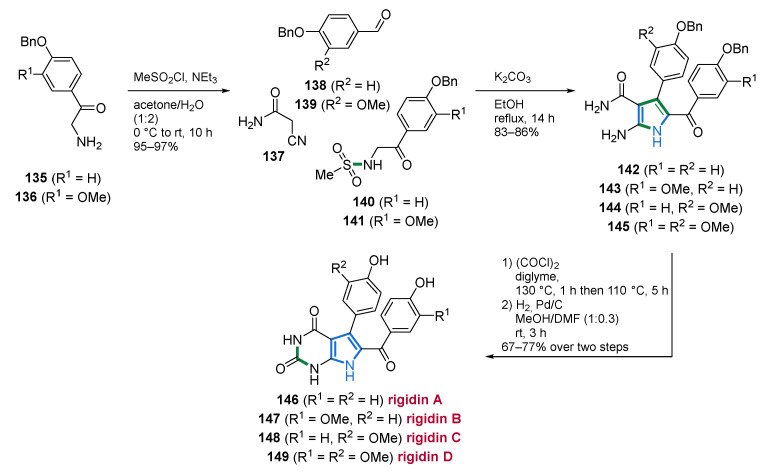
The so-far shortest synthetic approach towards rigidin A (**146**), including the first syntheses of rigidins B–D (**147**–**149**) in a one-pot multicomponent reaction.

The rigidins represent another prominent class of 2,3-fused pyrrole alkaloids, sharing a pyrrolo [2,3-*d*]pyrimidine scaffold [135]. With the first rigidin isolated back in 1990 by Kobayashi and co-workers [136], many MNPs belonging to this family have been isolated until today [137,138]. Although several total syntheses of rigidins are known [139,140,141,142,143], we want to mention the one-pot multicomponent reaction reported by the Magedov laboratory in 2011, which provides synthetical access to tetrasubstituted 2-aminopyrroles in only four steps and includes the first total syntheses of rigidins B–D (**147**–**149**) [144]. In a first step, *N*-(methanesulfonamido)acetophenones **140** and **141** were prepared from starting materials **135** and **136**, respectively. The multicomponent reaction was then realized by combining either **140** or **141** with aldehydes **138** or **139** under the addition of cyanoacetamide (**137**). The resulting 2-aminopyrroles **142**–**145**, isolated in 83–86% yield, were then converted into pyrimidinediones and after final deprotection, the rigidins A–D (**146**–**149**) could be obtained in four steps at an overall yield of 53–61% (Scheme 12) [144].

**Figure 18:**
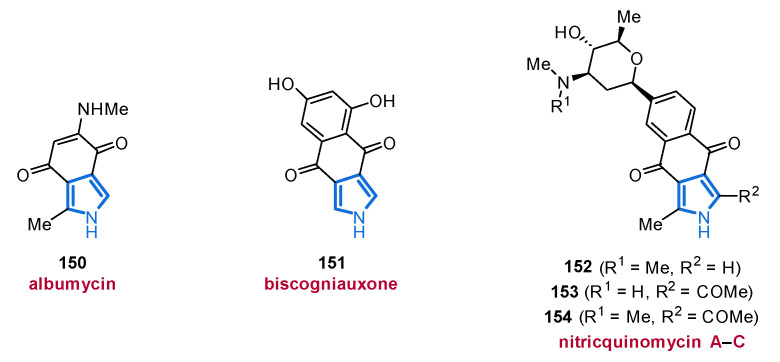
Series of isolated isopyrrolo-*p*-benzoquinone **150** and isopyrrolo-1,4-naphthoquinones **151**–**154**.

The annellated pyrrole alkaloids shown so far largely consist of a fused lactone or lactam structure, whereas 3,4-fused pyrroles often share a quinone system. This motif can be found in albumycin (**150**), a novel MNP isolated by heterologous expression from *Micromonospora rosaria* SCSIO N160 genes in *Streptomyces albus* J1074 (Figure 18). In antibacterial tests, only weak activities against several indicator strains were encountered (MIC values >64 µg/mL) [145].

In 2016, another fused *p*-quinone, biscogniauxone (**151**), was isolated from the marine fungus *Biscogniauxia mediterranea* and belongs to the rare family of isopyrrolonaphthoquinones (Figure 18) [146]. It should be mentioned that the authors assumed the existence of further derivatives of compound **151**, as metabolites with similar UV spectra were detected in the extracts, albeit without isolation. Significant inhibition of glycogen synthase kinase (GSK-3β, IC_50_ value 8.04 µM ± 0.28 µM) was observed for biscogniauxone (**151**), while weak inhibition of *Staphylococcus epidermidis* and *Staphylococcus aureus* was found (IC_50_ values in the range of 100 µM) [146].The nitricquinomycins A–C (**152**–**154**), isolated from *Streptomyces* sp. ZS-A45, complete the selection of isopyrrolonaphthoquinones (Figure 18) [147]. By comparing the spectroscopic data with those of previously reported naphthoquinones bearing a pyrrole core and using NOE experiments for the determination of the relative configuration, as well as ECD spectroscopy for the determination of the absolute configuration, the structure could be determined as indicated. Of compounds **152**–**154**, nitricquinomycin C (**154**) exhibited significant cytotoxicity against the human ovarian cancer cell line A2780 (IC_50_ value 4.77 µM ± 0.03 µM) but weak antibacterial potential against *Escherichia coli*, *Staphylococcus aureus*, and *Candida albicans* (MIC values > 40 µM) [147].

**Figure 19:**
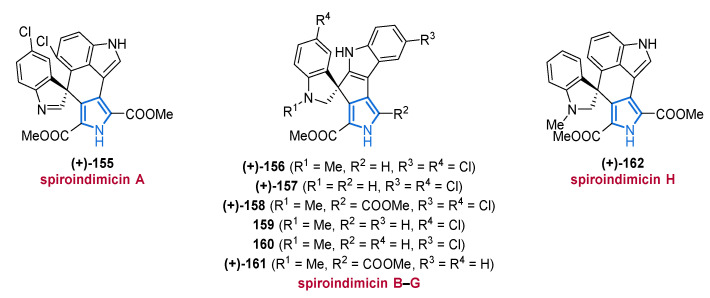
Structures of spiroindimicins A–H (**155**–**162**) isolated from marine actinobacteria.

Another 3,4-fused pyrrole family are the spiroindimicins (SPMs), which contain a remarkable spirocyclic bisindole framework and are highly related congeners of the bisindole pyrroles **59**–**66** (cf. Figure 8). Spiroindimicins A–D (**155**–**158**) were isolated from *Streptomyces* sp. SCSIO 03032 in 2012 [148]. The molecular structures were resolved by spectroscopic methods, with the 3D structures of spiroindimicin A (**155**) and B (**156**) being unambiguously confirmed by X-ray crystallography (Figure 19). Spiroindimicin A (**155**) consists of a [5.6] spirocyclic core, whereas congeners B–D **156**–**158** contain a [5.5] spirocyclic core. This structural difference also influences the bioactivity, which in the case of [5.5] spirocyclic pyrroles **156**–**158** results in good to moderate antitumor activities against various cancer cell lines with IC_50_ values ranging between 5 µg/mL and 22 µg/mL. Biosynthetic studies suggest the formation of spiroindimicins are proposed to derive from lynamicin by an aryl-aryl coupling of C-3′ and C-5″ or by an aryl-aryl coupling of C-3′ and C-2″, furnishing the [5.6] or [5.5] spiro-cyclic alkaloids, respectively [148].

The family of spiroindimicins was extended in 2017 by the monochlorinated compounds **159** and **160**, which were isolated from *Streptomyces* sp. MP131-18 (Figure 19) [149]. Spiroindimicins E (**159**) and F (**160**) did not show any activity against Gram-negative test cultures, being in line with the biological properties of their biosynthetic lynamicin-type precursors. In both cases, the antibacterial activity appears to increase with an increasing degree of chlorination on the bisindole backbone [149]. In addition to studies on the biosynthetic gene cluster of *Streptomyces* SCSIO 03032 [150], the group of Zhang, responsible for the isolation of spiroindimicins A–D (**155**–**158**), discovered the halogenase SpmH involved in the biosynthesis of SPMs and IDMs.

In 2019, inactivation of the encoding gene *spmH* then led to the isolation of spiroindimicins G (**161**) and H (**162**), which displayed moderate cytotoxicity against four cancer cell lines (IC_50_ values between 10.28 µM and 33.02 µM), comparable to their chlorinated congeners **155**–**160** (Figure 19) [151].

**Scheme 13:**
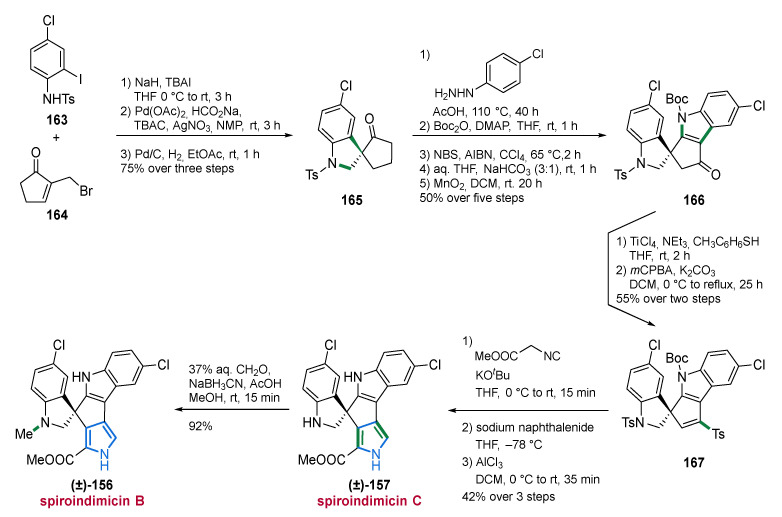
Total synthesis of spiroindimicins **156**, **157** using the Fischer indolization and Montforts pyrrole synthesis.

The first syntheses of these compounds were achieved by Sperry and co-workers in 2016 [152]. Starting with the alkylation of aniline **163** with bromide **164**, a subsequent Heck reaction and hydrogenation furnished the spirocyclic pentanone **165**. One key step is represented by the Fischer indolization, followed by Boc-protection and radical bromination. After hydrolysis and oxidation, ketone **166** was formed in 50% over five steps. Sequentially, a thioketal and then a vinylsulfone **167** were prepared which allowed for a Montforts pyrrole synthesis. After the final deprotection, (±)-spiroindimicin C (**157**) could be obtained. Additionally, reductive amination furnished (±)-spiroindimicin B (**156**) (Scheme 13) [152].

Further studies and recent publications highlight the importance of these bisindole alkaloids as promising bioactive compounds and potential new lead structures [153,154].

**Figure 20:**
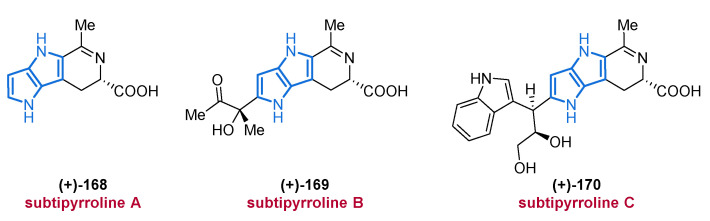
Subtipyrrolines A–C (**168**–**170**) as novel alkaloids from *Bacillus subtilis* SY2101.

The structurally remarkable subtipyrrolines A–C (**168**–**170**) incorporating a pyrrole-pyrrole-dihydropyridine framework, were isolated from the *Bacillus subtilis* SY2101 strain, derived from sediment samples of the Mariana Trench collected at a depth of 11,000 m (Figure 20) [155]. The structural elucidation was investigated by spectroscopic analysis and supported by X-ray crystallography. Bioactivity assays revealed moderate antiproliferative activities (human glioma U251 and U87MG cells, IC_50_ values of 36.3 µM and 26.1 µM) as well as moderate antimicrobial potential (*Escherichia coli* and *Candida albicans*, IC_50_ values between 34 µM and 46 µM, respectively) [155].

#### 2.4.1. Lamellarins and Related Natural Congeners

To date, more than 65 lamellarins have been discovered since the first isolation of a member of this class by Faulkner et al. in 1985 [156,157]. Divided into type I (with subsections a and b, comprising compounds with a saturated or unsaturated C-5–C-6 unit, respectively) containing a doubly annellated 2,3,4-triarylpyrrole core in form of a 1-aryl-6*H*-chromeno-[4′,3′:4,5]pyrrolo-[2,1-a]isoquinolin-6-one or type II with a simple 3,4-diarylpyrrol-2-carboxylate ring system, the lamellarins comprise a large and prominent class of marine alkaloids. These compounds, derived from sponges, tunicates, and mollusks, exhibit a broad range of often highly potent biological activities, making them interesting targets for synthetic chemists [157,158].

**Figure 21:**
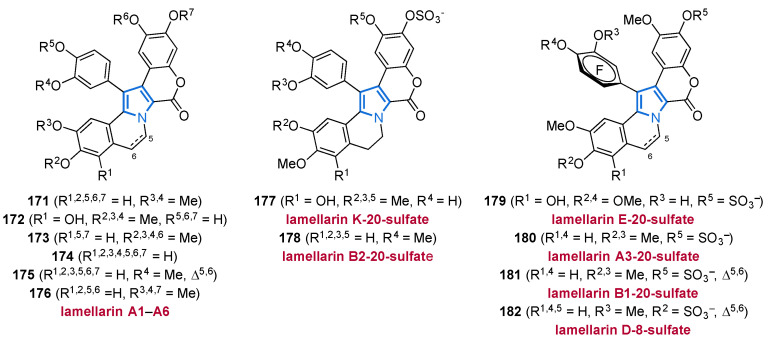
Members of the lamellarins **171**–**182** (type I) isolated from *Didemnum* sp. in 2012 and 2019.

In 2012, Capon and co-workers investigated *Didemnum* sp. and isolated five new lamellarins A1–A5 (**171**–**175**) from the strain CMB-01656 and one further member (A6, **176**) from the strain CMB-02127 (Figure 21) [159]. Together with eight known derivatives, a structure–activity relationship (SAR) study was performed regarding the reversal of multidrug resistance. In the SAR study, the P-glycoprotein (P-gp) inhibition activity was proposed to increase with a higher degree of O-methylation. The synthesis of a permethylated derivative, featuring potential non-cytotoxic P-gp inhibitory activities then confirmed this assumption [159].

The lamellarin sulfates represent a small subclass within the lamellarin family. In 2019, the group of Keyzers isolated six new lamellarin sulfates (**177**–**182**) from *Didemnum ternerratum*, a pacific tunicate (Figure 21) [160]. All of them showed similar analytical data to previously reported lamellarins except for the sulfate functional group. The substantial majority of naturally occurring lamellarins show no optical rotation with the exception of lamellarin S (half-life of racemization ≈ 90 days). Surprisingly, the newly isolated sulfates **179**–**182** showed optical activity in ECD analysis, which is due to the hindered rotation of ring F resulting in an axial chirality (atropisomerism). The bioactivity of lamellarins **177**–**182** against human colon carcinoma HCT-116 was investigated, with D-8-sulfate (**182**) showing appreciable cytotoxicity (IC_50_ = 9.7 µM) [160].

In addition to the representative group of lamellarins [32,156,161,162,163,164,165,166], further related pyrroles like the polycitons, polycitrins [167], storniamides [168], and denigrins [90,169] as well as the fused alkaloids lukianols [170], dictyodendrins [171], purpurone [172], ningalins [173] and baculiferins can also be included, which extend the family of 3,4-diarylpyrroles. In the molecular backbone, structural variations from fused maleiimide units to highly conjugated carbazole-2,7-diones can be found.

**Figure 22:**
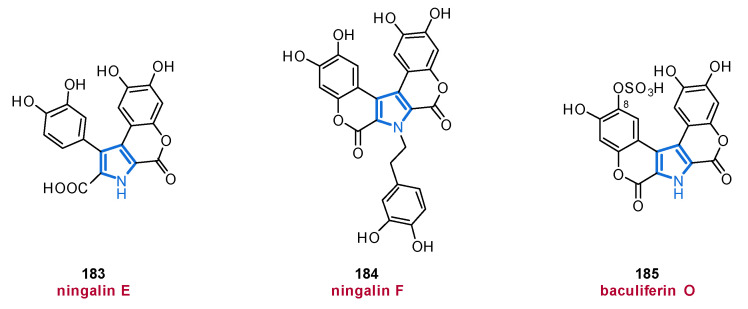
Related congeners **183**–**185** of the lamellarins sharing the central fused pyrrole core.

The Capon laboratory isolated the new ningalins E (**183**) and F (**184**) from the species *Didemnum* (CMB-02127), which, according to the authors, share a biosynthetic pathway similar to that of the lamellarins by merging a tyrosine with a defined number of catechols (Figure 22). Only low cytotoxicities against human, bacterial, and fungal cell lines were observed, whereas the ningalins **183** and **184** showed moderate inhibition of the kinases CK1δ, CDK5, and GSK3β, potential targets for the treatment of neurodegenerative diseases (IC_50_ values between 1.6 µM and 10.9 µM) [174].

The class of the baculiferins was established by Lin and Bringmann in 2010, yielding pyrrole **185** alongside 14 other new members bearing a carbazole-2,7-dione central core (Figure 22). Baculiferin O (**185**) as a C8 sulfate representative inhibits several tumor cell lines with moderate activity around 33 µM [175].

Because of their promising biological activities such as antiproliferative, multidrug resistance reversal activity, cytotoxicity, and anti-HIV-1 activity, the lamellarin core has served as a potential lead structure for synthetic and medicinal chemists in the past decade [157,158]. The published syntheses of the lamellarins and derivates in the past decade, summarized in Table 1, provide an update of the existing summary by Opatz et al. in 2014 [158] and concentrate the recent review by Iwao et al. in 2020 [157].

This astounding number of syntheses highlights the importance of these pyrrole members of marine origin to many areas of life science. In addition to the constantly increasing number of total syntheses of lamellarins and their natural congeners, the number of synthetic derivatives and biological activity assays has increased similarly [206,207,208,209,210,211,212,213,214].

## 3. Halogenated Marine Pyrrole Alkaloids

This chapter presents the occurrence of halogenated pyrroles which constitute a highly diverse and structurally complex subclass of marine alkaloids. It is considered that at least 25% of organohalogen natural products are halogenated alkaloids, mostly featuring pyrrole, indole, carboline, and other N-heteroaromatic core structures [215,216]. This observation is not too surprising as the marine environment provides both chloride and bromide in virtually unlimited quantities as well as a variety of halogenase enzymes from different organisms, resulting in an excellent environment for biohalogenation of these electron-rich substrates [30,217,218]. From a medicinal point of view, the resulting structures are associated with numerous different pharmacological activities such as selective anti-histamine [219,220,221], anti-serotonergic [222], immunosuppressive [223], antibacterial [224], anti-malarial [225], and antiproliferative properties [226]. Therefore, halogenated pyrrole alkaloids can be viewed as potential lead compounds for the development of new, even more potent drugs [15,227].

Given the enormous dimensions and (bio)chemical diversity of marine life and its underexplored nature, it is not surprising that the number of isolated halogenated marine pyrroles is constantly increasing and that countless further halopyrroles are yet to be discovered.

### 3.1. Simple Pyrroles

**Figure 23:**
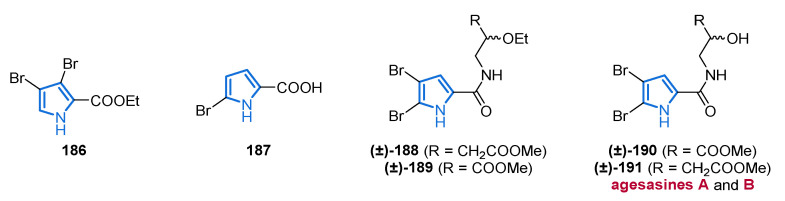
Simple bromopyrrole alkaloids **186**–**191** isolated from different marine sponges.

Ethyl 3,4-dibromo-1*H*-pyrrole-2-carboxylate (**186**) was first isolated from the sponge *Stylissa massa* in 2014 and shows a weak antiproliferative activity against mouse lymphoma cells (L5178Y growth in 27.2% at 10 µg/mL, Figure 23) [228].

A related bromopyrrole **187** was isolated from another sponge (*Agelas cerebrum*) in 2011 and subjected to several antiproliferative tests (Figure 23) [229]. Here, compound **187** and other isolated bromopyrroles did not show any activity against cancer cells (A549 lung cancer cells, HT29 colonic cancer cells, and MDA-MB-231 breast cancer cells). However, when the crude mixture, from which **187** and further bromopyrroles were isolated, was subjected to biological tests, a strong cytotoxic activity (IC_50_ values around 1 µg/mL) against all three human tumor cell lines could be observed. The authors attributed this effect to the yet underexplored synergism of natural product mixtures containing bromopyrroles [229]. Both compounds **186** and **187** were previously only known as synthetic products [230,231].

Two further simple substituted halopyrroles, **188** and **189**, could be isolated from the South China Sea sponge *Agelas* sp. in 2016. The enantiomers (+)-**188**, (−)-**188**, (+)-**189** and (−)-**189** did not appear to have any antifungal activities using the *Caenorhabditis elegans* candidiasis model (Figure 23) [66]. However, the racemic mixtures of (±)-**188** and (±)-**189** showed effective antifungal activity. Unfortunately, the authors did not provide any values or an explanation of this observation. Despite these results, the authors found out that the corresponding intramolecularly cyclized pyrroloketopiperazine natural products (see Figure 49, **342**–**344**) exhibited significant antifungal activities with survival rates around 50% [66].

Very recently, the corresponding agesasines A (**190**) and B (**191**) featuring the free alcohol functional groups, were isolated from Okinawan marine sponges *Agelas* spp. (Figure 23) [232]. Both compounds were isolated as racemates and, according to the authors, might be artifacts from the extraction process under acidic conditions. In basic antiproliferative tests against human cancer cell lines (HeLa, A549, and MCF7), no cytotoxicity could be observed [232].

**Figure 24:**
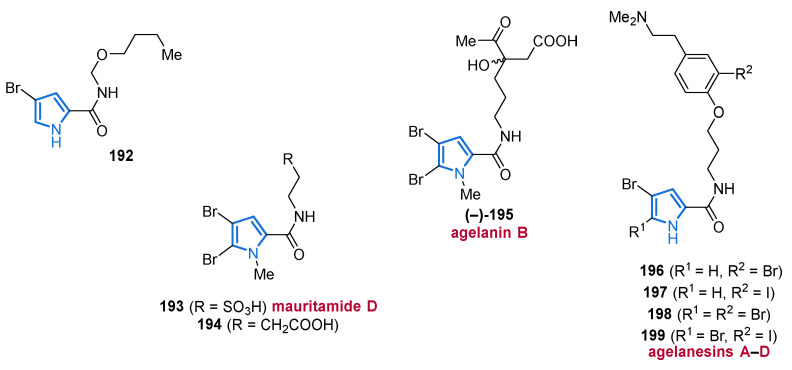
Simple bromopyrrole alkaloids **192**–**195** and structural similar agelanesins A–D (**196**–**199**).

In 2012, a new bromopyrrole, 4-bromo-*N*-(butoxymethyl)-1*H*-pyrrole-2-carboxamide (**192**), featuring an unusual ether group in its side chain, could be isolated from the marine sponge *Agelas mauritiana* (Figure 24) [233].

Further structurally similar halopyrroles **193**–**199** possessing different substituents at their amide side chains were isolated from the Indonesian marine sponges *Agelas linnaei* (Figure 24) [234]. While mauritamide D (**193**), 4-(4,5-dibromo-1-methylpyrrole-2-carboxamido)-butanoic acid (**194**), and agelanin B (**195**) were inactive against L1578Y mouse lymphoma cell lines, the tyramine-unit bearing agelanesins A–D (**196**–**199**) showed prominent to good activity with IC_50_ values between 9.25 µM and 16.76 µM in this assay. The authors mentioned that the cytotoxicity of the agelanesins **196**–**199** is interconnected with the degree of bromination of the pyrrole ring, resulting in an increased reactivity for the monobrominated agelanesins A (**196**) and B (**197**) compared to **198** and **199** [234].

**Figure 25:**
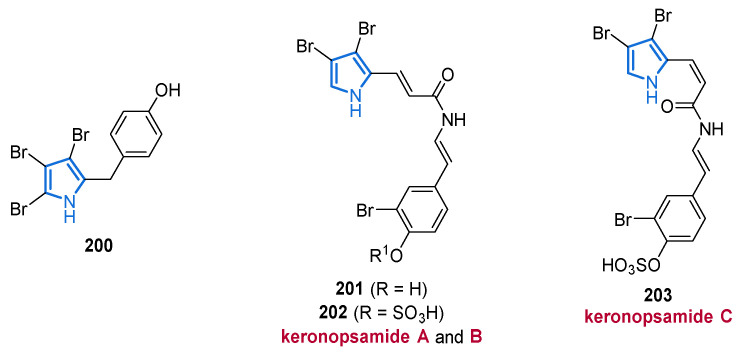
Structure of compound **200** and the bromotyrosine-based keronopsamides A–C (**201**–**203**).

The tribrominated pyrrole 4′-((3,4,5-tribromo-1*H*-pyrrol-2-yl)methyl)phenol (**200**) was isolated from the surface of the coralline alga *Neogoniolithon fosliei* in 2014 and exhibited broad-spectrum antibacterial activity against several *Pseudoalteromonas*, *Vibrio*, and *Staphylococcus* spp. (inhibition zones > 10 mm, Figure 25). However, no antifungal or antiprotozoal activity was observed by investigating compound **200** [235].

A new class of bromopyrrole pigments derived from bromotyrosine were isolated from the marine ciliate *Pseudokeronopsis riccii* in 2010 and were named keronopsamides A–C (**201**–**203**) (Figure 25) [236].

**Figure 26:**
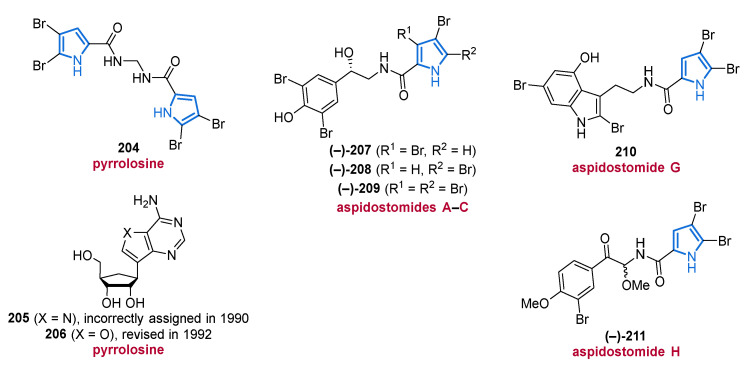
Molecular structures of bromopyrroles **204**–**211** isolated from sponges and bryozoans.

In 2020, pyrrolosine (**204**), a tetrabrominated alkaloid symmetrically dimerized via two amide functionalities, was isolated from *Agelas oroides* [237] and should not be confused with another natural product named pyrrolosine (**206**), the structure of which had been identified as **205** and revised **206** during the 1990s (Figure 26) [238].

Further marine bromopyrrole alkaloids **207**–**211** substituted via amide groups were isolated from the Patagonian bryozoan *Aspidostoma giganteum* (Figure 26) [239]. The aspidostomides A–C (**207**–**209**), G (**210**) and H (**211**) bear the well-known bromotyrosine and bromotryptophan structural motifs frequently found in marine natural products [240]. While for aspidostomide A (**207**) the absolute configuration was determined as *R* by a modified Mosher method [241], the configurations of aspidostomides B (**208**) and C (**209**) were assumed to be the same as in compound **207**. The absolute configuration of aspidostomide H (**211**) could not yet be established [239].

**Scheme 14:**
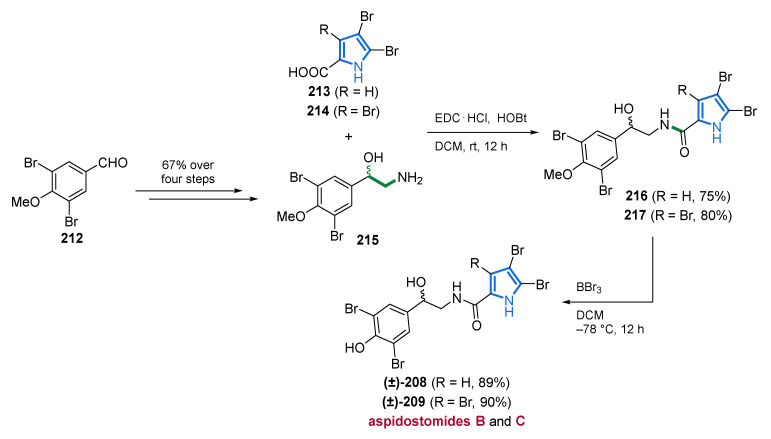
First total syntheses of aspidostomides B (**208**) and C (**209**) starting from compound **212**.

In 2019, the first total syntheses of the enantiomeric aspidostomides B (**208**) and C (**209**) were realized by Khan and co-workers (Scheme 14) [242].

Here, compound **212** was reacted in a Wittig olefination and then subjected to bromohydroxylation. Substitution of the bromine with NaN_3_ followed by reduction furnished amine (±)-**215** in 67% yield over four steps. Amidation of (±)-**215** with either 4,5-dibromopyrrole carboxylic acid (**213**) or 3,4,5-tribromopyrrole carboxylic acid (**214**) delivered products **216** and **217**, respectively. Final demethylation by applying BBr_3_ then gave the natural products aspidostomides B (**208**) in 67% and C (**209**) in 72% over two steps (Scheme 14) [242].

**Figure 27:**
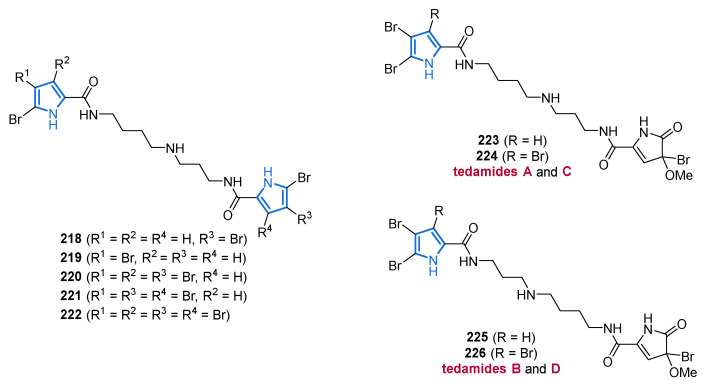
Nine new pseudoceratidines **218**–**226** from the marine sponge *Tedania brasiliensis*.

In 2018, nine new pseudoceratidines (**218**–**226**), of which the tedamides A–D (**223**–**226**) possess an unprecedented 4-bromo-4-methoxy-5-oxo-4,5-dihydro-1*H*-pyrrole-2-carboxamide moiety, were isolated from the marine sponge *Tedania brasiliensis* (Figure 27) [243]. It is important to mention that 3-debromopseudoceratidine (**218**) and 20-debromopseudoceratidine (**219**), 4-bromopseudoceratidine (**220**), and 19-bromopseudoceratidine (**221**), tedamides A and B (**223** and **225**), and tedamides C and D (**224** and **226**) have been isolated as pairs of inseparable structural isomers differing in their sites of bromination and oxidation. The inseparable mixture of compounds **218** and **219** showed antiparasitic activity on *Plasmodium falciparum* (EC_50_ value of 5.8 µM ± 0.5 µM) and displayed weak cytotoxicity in the human liver cancer HepG2 cell line (MDL_50_ ≥ 400 µM), but with excellent selectivity, as reflected by a dramatically reduced toxicity to healthy cells. The authors also synthesized a number of derivatives that were assayed against several protozoan parasite species, evidencing that the bromine substituents in the pyrrole unit of pseudoceratidine derivatives are inevitable for antiplasmodial activity [243].

**Figure 28:**
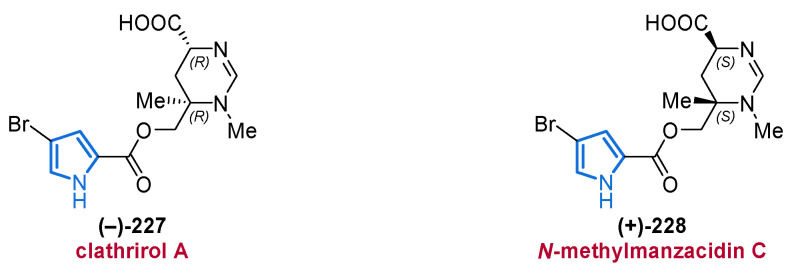
New bromopyrrole alkaloid **227**. *N*-Methylmanzacidin C (**228**) is shown for comparison.

Another bromopyrrole alkaloid, clathrirole A (**227**), was isolated from the Myanmarese marine sponge *Clathria prolifera* in 2018 (Figure 28) [73]. It should be noted that the stereogenic centers of the tetrahydropyrimidinium ring of **227** were only assumed to have *R* configuration by comparison of its optical rotation with the enantiomeric *N*-methylmanzadicin C (**228**) which had been isolated and synthesized several years earlier [35,244,245].

**Scheme 15:**
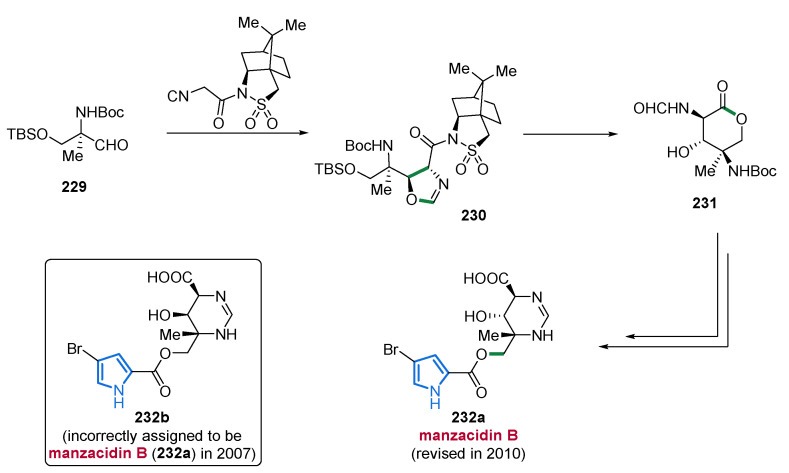
An alternative synthetic route towards manzacidin B (**232a**) in 2010 revealed that it was incorrectly assigned as compound **232b** in 2007.

In this context, the correction of the stereoconfiguration of manzacidin B (**232a**) should also be mentioned. This MNP was synthesized by the Ohfune group in 2007 and its configuration was erroneously determined to match compound **232b [246]**. Three years later, the same group published an alternative synthetic route (Scheme 15) and with the aid of X-ray crystallography, the revised structure of manzacidin B (**232a**) was unambiguously confirmed [247]. Here, aldehyde **229** was transformed into compound **230** using Oppolzer’s sultam as a chiral auxiliary, and subsequently generated the *N*-formyl lactone **231** already featuring the stereochemistry of natural manzacidin B (**232a**). Several further steps, including the installation of the pyrrole unit, then delivered the natural product **232a [247]**. Unfortunately, the correction did not provide any information about the experimental section, including reaction conditions and yields.

**Figure 29:**
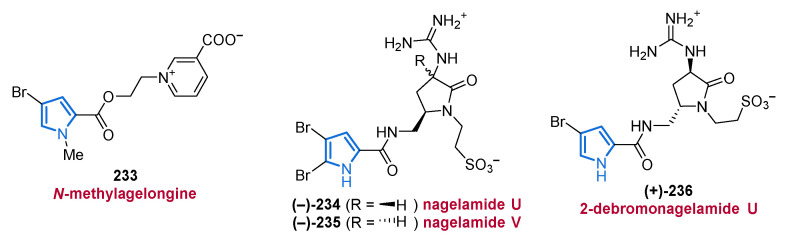
Simple bromopyrrole alkaloids **233**–**236** isolated from the *Agelas* sp.

In 2015, the group of Köck isolated *N*-methylagelongine (**233**) from the Caribbean sponge *Agelas citrina* (Figure 29) [63].

Two new halopyrroles, nagelamide U and V (**234** and **235**) were isolated from a marine sponge *Agelas* sp. in 2013 and possess a γ-lactam ring with a taurine unit (Figure 29). Here, the relative stereochemistry was examined by ROESY correlations [65].

A related compound, 2-debromonagelamide U (**236**) was isolated from the Okinawan marine sponge *Agelas* sp. two years later. Compound **236** could inhibit the growth of *Trichophyton mentagrophytes* (IC_50_ value 16 µg/mL), a common fungus causing ringworm in companion animals (Figure 29) [248].

**Figure 30:**
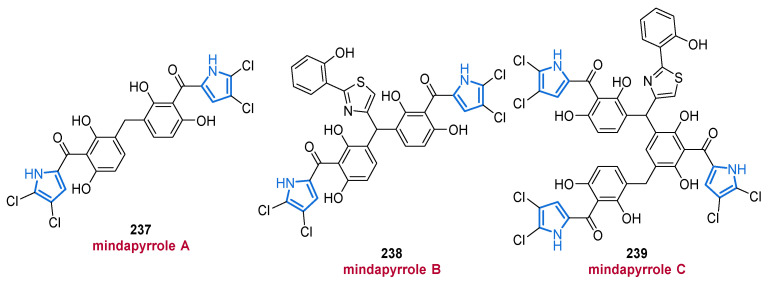
Mindapyrroles A–C (**237**–**239**) featuring several central resorcinol-cores.

In 2019, three new pyoluteorin analogs, mindapyrroles A–C (**237**–**239**) were isolated from *Pseudomonas aeruginosa* strain 1682U.R.oa.27, a bacterium from the tissue homogenate of the giant shipworm *Kuphus polythalamius* (Figure 30) [249]. The chlorinated pyrrole alkaloids **237** and **239** inhibit the growth of multiple clinically relevant microbial pathogens (MIC values between 2 µg/mL and >32 µg/mL), with mindapyrrole B (**238**) showing the most potent antimicrobial activity (MIC values between 2 µg/mL and 8 µg/mL) and widest selectivity index over mammalian cells [249].

**Figure 31:**
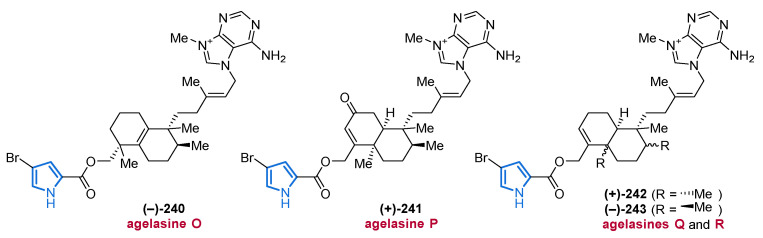
Agelasines O–R (**240**–**243**) with a 9-*N*-methyladenine unit from a marine sponge *Agelas* sp.

New diterpene alkaloids, the agelasines O–R (**240**–**243**) bearing a bromopyrrole core, were isolated from the Okinawan marine sponge *Agelas* sp. in 2012 (Figure 31) [61]. The relative stereochemistries of compounds **240**–**243** were elucidated via ROESY-correlations. The agelasines O–R (**240**–**243**) showed good to moderate antimicrobial activities (IC_50_ values ranging between 8 µg/mL and >32 µg/mL) against a wide range of bacteria, including strains of *Escherichia coli*, *Staphylococcus aureus*, and *Bacillus subtilis*. However, no cytotoxicity against murine leukemia L1210 and human epidermoid carcinoma KB cells was observed [61].

**Figure 32:**
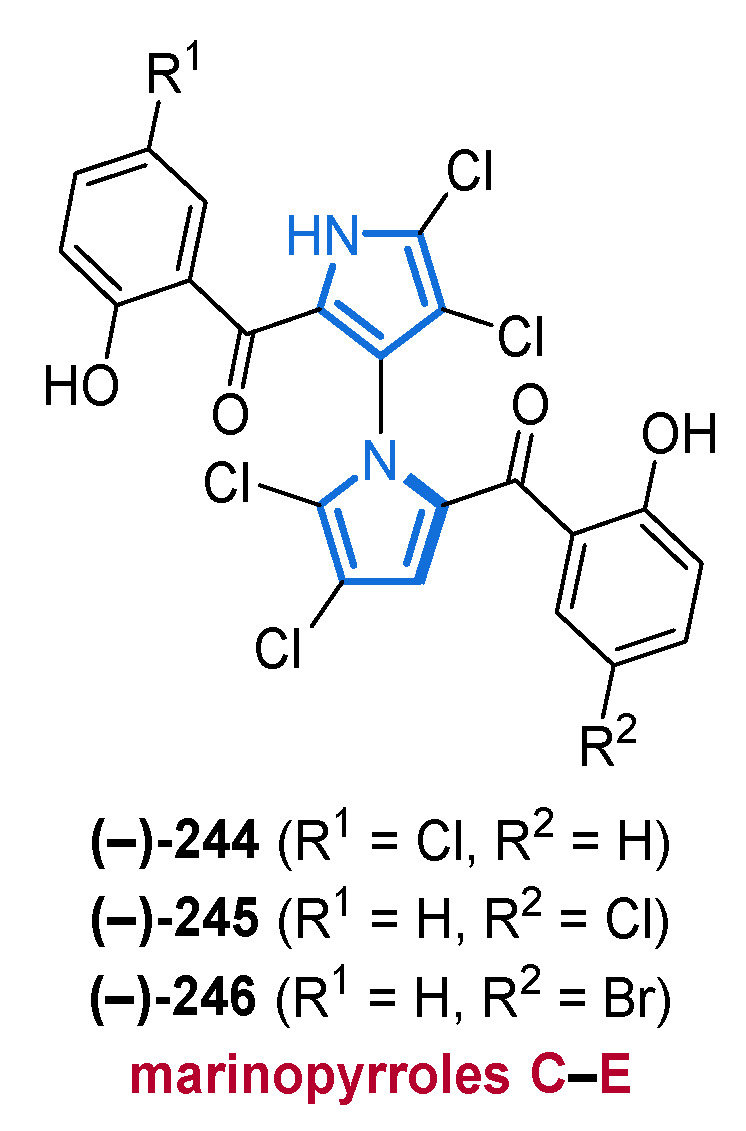
The unusual structure of marinopyrroles C–E (**244**–**246**) contain a rare 1,3′-bispyrrole functionality.

In 2010, Fenical and co-workers isolated marinopyrroles C–E (**244**–**246**) from the deep ocean actinomycete strain CNQ-418 [250], thereby extending the interesting class of biologically active marinopyrroles, of which marinopyrroles A (**250**) and B (**253**) had been isolated before (Figure 32) [251]. These metabolites contain an unprecedented, highly halogenated 1,3′-bipyrrole core which gives them an axis of chirality that, for marinopyrroles A and B as well as C–E (**244**–**246**), results in a stable *M*-configuration at room temperature. Marinopyrrole C (**244**) displayed significant activity against methicillin-resistant *Staphylococcus aureus* with MIC_90_ values of less than 1 µg/mL. With derivatization experiments, the authors could also show that the presence of the hydrogen-bonding capacity of the salicyloyl hydroxyl groups, the free N–H functionality and the C-5′ chlorine substituent were indispensable for the biological activity [250].

**Scheme 16:**
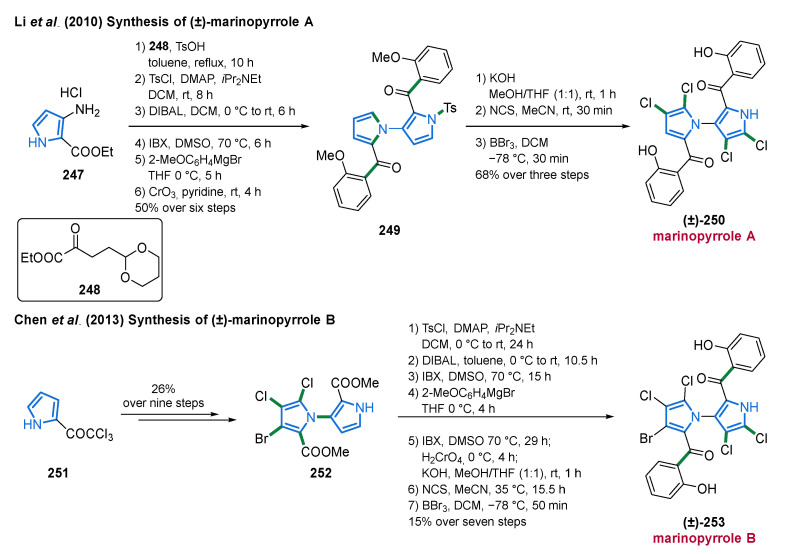
First total synthesis of (±)-marinopyrrole A (**250**) by Li in 2010 and its congener marinopyrrole B (**253**) by Chen in 2013.

The first total synthesis of a member of the marinopyrrole family was realized by the Li laboratory in 2010 (Scheme 16) [252]. Starting with a TsOH-catalyzed condensation and cyclization of aminopyrrole **247** with α-ketoester **248** furnished an intermediary bi-pyrrole skeleton. After N-protection and transforming the diester to the dialdehyde via a reduction/oxidation sequence, the addition of 2-methoxyphenylmagnesium bromide followed by CrO_3_ oxidation furnished the diketone **249** in 50% over six steps. After deprotection and chlorination of the pyrrole units with NCS, a final demethylation involving BBr_3_ gave the natural product, (±)-marinopyrrole A (**250**) in 68% yield over three steps. Unfortunately, selective bromination towards (±)-marinopyrrole B (**253**) under various conditions was unsuccessful [252].

Three years later, the Chen laboratory synthesized (±)-marinopyrrole B (**253**) using a similar approach (Scheme 16) [253]. Here, the brominated chloropyrrole **252** was generated over nine steps starting from commercially available pyrrole **251**. The next seven steps were performed almost in the same manner as in the synthesis of marinopyrrole A reported by Li and co-workers, although some reaction conditions were improved. In this way, (±)-marinopyrrole B (**253**) could be obtained in 15% over seven steps [253].

**Figure 33:**
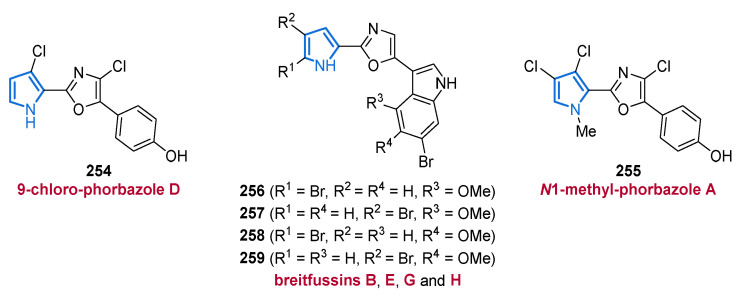
Phorbazol-based marine bromopyrrole alkaloids **254**–**259**.

Between 2012 and 2019, several pyrrolyloxazoles belonging to the phorbazole series were isolated from marine organisms. The first study of the Indo-Pacific dorid nudibranch *Aldisa andersoni* resulted in the isolation of 9-chloro-phorbazole D (**254**) and *N*1-methyl-phorbazole A (Figure 33) (**255**). Both compounds exhibit similar in vitro inhibitory activity against several human cancer lines with IC_50_ values ranging between 18 µM and 34 µM [254].

A related class of natural bromopyrroles containing the pyrrolyloxazole functionality is the breitfussins. In analogy to breitfussin B (**256**), isolated from the hydrozoan *Thuiaria breitfussi* in 2012 [80], six new breitfussins C–H were discovered in the same producing organism as breitfussins E (**257**), G (**258**), and H (**259**) feature a brominated pyrrole core (Figure 33, for non-halogenated congeners see Figure 7) [81]. Compounds **258** and **259** were isolated as a mixture and thus not evaluated in cytotoxic activity assays, whereas breitfussins **256** and **257** did not show any cytotoxic activity against several tested cancer cell lines [81].

**Scheme 17:**
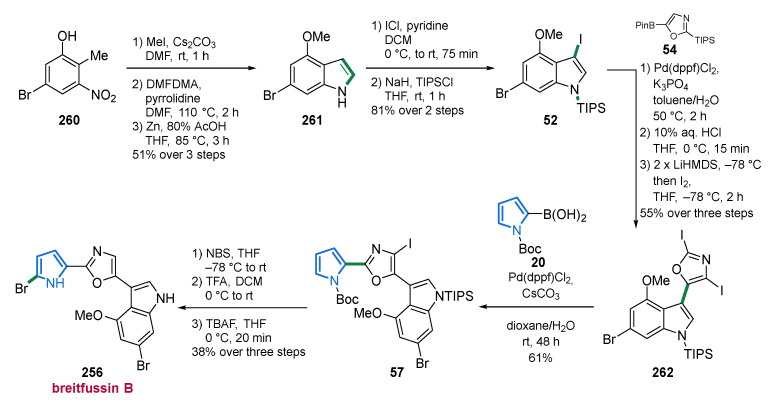
Total synthesis of breitfussin B (**256**) starting from phenol **260**.

In 2015, breitfussin B (**256**) was synthesized by the Bayer group in the same manner as breitfussin A (**48**) (compare Scheme 4) [83]. In analogy to breitfussin A (**48**), the synthesis commenced with the readily available phenol **260**. After forming the indole building block **261**, iodination and TIPS-protection furnished compound **52**. The oxazole core **54** was installed and carefully iodinated with iodine to get access to compound **262**. Coupling with Boc-protected pyrrole boronic acid **20** then delivered intermediate **57** possessing the right indole-pyrrolyloxazole functionality. Bromination, protodeiodination, and removal of all protecting groups then furnished breitfussin B (**256**) in 4.3% overall yield (Scheme 17) [83].

#### Simple Pyrrole (Amino)-Imidazole Alkaloids

The pyrrole-imidazole alkaloid (PIA) family comprises a myriad of simple to structurally complex molecules originating from marine organisms. The simplest PIA, oroidin, is believed to be the biogenetic precursor of any natural products belonging to this family and it is considered to be biosynthesized from the fundamental amino acids proline, ornithine, lysine, and/or histidine [13,38,255,256,257]. However, numerous further considerations on the biogenetical origin of PIAs can be found in the literature so that the biosynthesis of most of these alkaloids still lies in the realm of speculations. Many PIAs are reported to exhibit significant biological activities resulting in a great interest among synthetic chemists to provide solutions to finally get access to potent pharmaceutically relevant substances.

**Figure 34:**
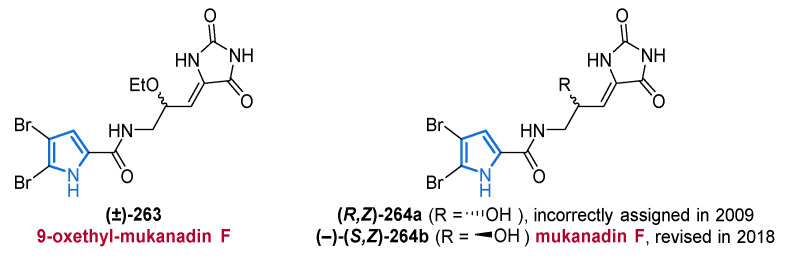
C-9 functionalized ene-hydantion marine pyrrole alkaloids **263** and **264**.

In 9-oxethyl-mukanadin F (**263**), isolated in 2016 by the Lin group from a not fully identified sponge *Agelas* sp., the oroidin 2-aminoimidazole moiety is replaced by a hydantoin ring (Figure 34) [66]. Compound **263** was isolated as a racemic mixture and displayed no antifungal activity against *Candida albicans* [66].

**Scheme 18:**
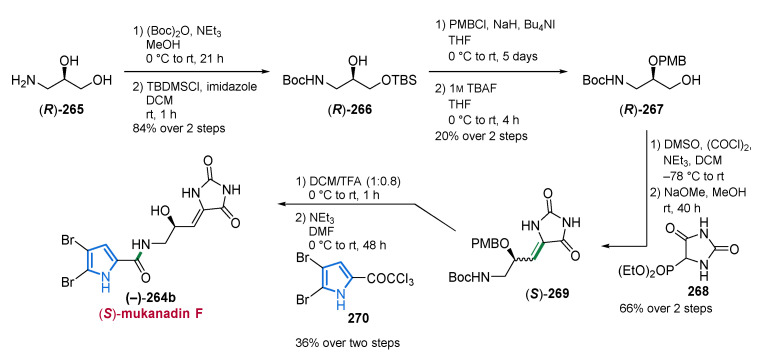
Total synthesis of (*S*)-mukanadin F (**264b**).

In 2018, the Barker group published a comprehensive work addressing stereochemical issues of related mukanadin-based alkaloids substituted at C-9 [79]. The publication also describes the total synthesis of (+)- and (−)-mukanadin F (**264a** and **264b**), which finally resulted in the reassignment of its absolute stereochemical configuration and shed light upon many inconsistencies concerning the stereochemistry of C-9-functionalized ene-hydantoin/imidazole marine natural products published as racemic or scalemic mixtures before (Figure 34 and Scheme 18) [220,258,259,260,261].

The authors began the synthesis with a selective protection/deprotection sequence of aminodiol (*R*)-**265** producing alcohols (*R*)-**266** and (*R*)-**267**, sequentially. After Swern oxidation and HWE reaction with hydantoin phosphonate **268**, compound (*S*)-**269** could be obtained as a mixture of *E*/*Z* isomers (1:2) in 66% yield over two steps. Simultaneous Boc and PMB deprotection followed by a final C−N coupling step involving trichloroacetyl dibromopyrrole **270** gave (*S*)-mukanadin F ((*S*)-**264b**) as a mixture of *E*/*Z* isomers (1:1.3). The same procedure starting from (*S*)-**265** delivered (*R*)-mukanadin F ((*R*)-**264a**) as a mixture of *E*/*Z* isomers (1:2) (Scheme 18) [79].

Successful separation of the *E*/*Z* isomers of ((*S*)-**264b**) and ((*R*)-**246a**) and comparison of NMR spectroscopic data of the synthetic *Z*-configured enantiomers of mukanadin F (**264**) with those reported for the natural product were a match, confirming the alkene geometry [258]. However, new optical rotation measurements revealed that (*S*)-mukanadin F ((*S*)-**264b**) corresponds to the natural product, which is opposite to that proposed for the isolated sample in 2009 [258]. As a last point, the Baker group found out that C-9 functionalized ene-hydantoin/imidazole marine alkaloids are prone to isomerization and racemization with both effects occurring upon light irradiation or under acidic or basic conditions and therefore is likely to occur upon extraction [79]. These findings reveal that compounds of this class most likely exist in nature as pure enantiomers and that other publications concerning their isolation and stereochemical elucidation should be checked carefully.

**Figure 35:**
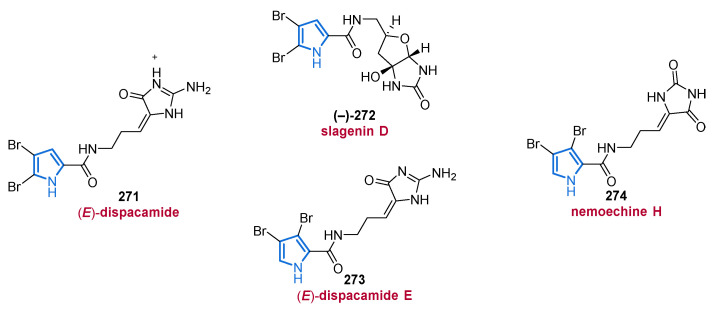
Related bromopyrrole alkaloids **271**–**274** bearing hydantoin.

Recently, *E*-dispacamide (**271**) and slagenin D (**272**) were isolated from the sponge *Agelas oroides* in 2020 (Figure 35). The absolute configuration of compound **272** was established by comparison of its specific rotation with that of synthetic *ent*-slagenin A, indicating its stereogenic centers to be 9*S*, 11*S*, 15*S* configured [237].

A bromopyrrole marine alkaloid **273**, very similar to compound **271**, was isolated from the sponge *Stylissa massa* in 2014 and was given the name dispacamide E (**273**) (Figure 35) [228]. It showed significant inhibitory activities against the kinases GSK-3, DYRK1A, and CK-1 with IC_50_ values below 19 µM [228]. The reader is advised that careful reading is required to distinguish between the (*E*/*Z*) dispacamides, as the original trivial names relate to the *Z*-configured natural compounds [219,220]. However, new dispacamides possessing *E*-configuration are not consistently given either new trivial names or *E*/*Z*-designated former trivial names.

In nemoechine H (**274**), isolated from the sea sponge *Agelas nemoechinata* in 2019, only the hydantoin core is different compared to compound **273** (Figure 35). Compound **274** exhibited good to moderate cytotoxic activity against K562 and L-02 cell lines with IC_50_ values of 6.1 µM and 12.3 µM, respectively [262].

**Figure 36:**
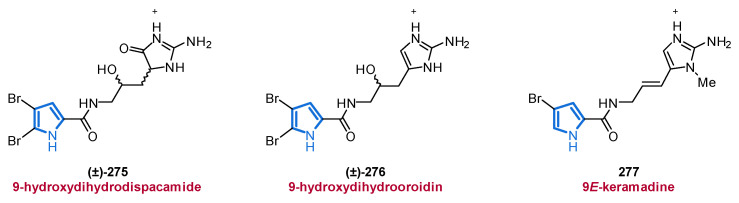
Three new PIAs **275**–**277** isolated from the sponge *Agelas* spp. in 2020.

Very recently, three new related congeners, 9-hydroxydihydrodispacamide (**275**), 9-hydroxydihydrooroidin (**276**), and 9*E*-keramadine (**277**) were isolated from two different marine sponges *Agelas* spp. (Figure 36). Compounds **275** and **276** were isolated as racemates with the relative configuration of compound **275** still to be deduced [232]. Compound **277** was already known as a synthetic product but was isolated the first time from a natural source [263]. All three compounds **275**–**277** did not show any promising cytotoxicity against human cancer cell lines (HeLa, A549, MCF7) in basic antiproliferative tests [232].

**Figure 37:**
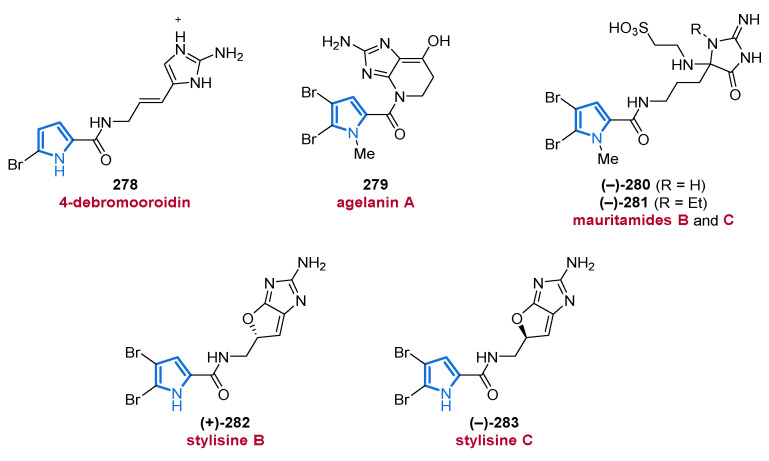
Oroidin-derived bromopyrrole alkaloids **278**–**283** bearing imidazole moieties.

The Berlinck group isolated debromooroidin **278** from a sponge identified as *Dictyonella* sp. in 2018, which displayed proteasome inhibition activity with IC_50_ values of 27 µM ± 6 µM (Figure 37) [264]. The authors also mentioned that the proteasome inhibitory activity is strongly influenced by the position of the bromine substituent in the pyrrole ring thereby confirming the findings of previous investigations [265,266].

In 2009, the acetone/methanol extract of the sponge *Agelas linnaei* permitted the isolation of agelanin A (**279**) and mauritamides B (**280**) and C (**281**) (Figure 37) [234]. The sulfonic acid congeners **280** and **281** contain a taurine unit which is quite a rare structural motif in marine sponge metabolites when combined with a bromopyrrole unit.

Further oroidin-derived pyrrole alkaloids, stylisines B (**282**) and C (**283**), were isolated in 2018 from the sponge *Stylissa massa* (Figure 37). Here, the stereogenic centers could be unambiguously determined via electronic circular dichroism experiments. Unfortunately, compounds **279**–**283** have not shown any promising biological activities so far [133].

**Figure 38:**
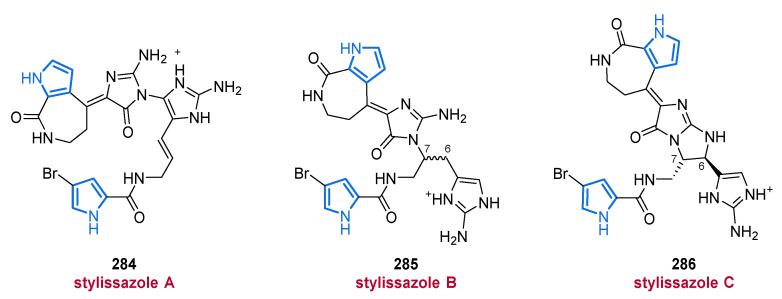
Stylissazoles A–C (**284**–**286**) isolated from the marine sponge *Stylissa carteri*.

In 2010, another new set of halopyrroles, the stylissazoles A–C (**284**–**286**), were isolated from species from the *Stylissa* genus (Figure 38) [267]. No absolute configuration could be determined for the dimeric pyrrole-2-aminoimidazoles **285** and **286** as no optical activity was observed. The authors mentioned that the interconversion of the configurationally unstable chiral carbons C6 and C7 might be the reason for this issue. However, the relative configuration of both stereogenic centers in stylissazole C (**286**) could be determined by NOESY experiments [267].

**Figure 39:**
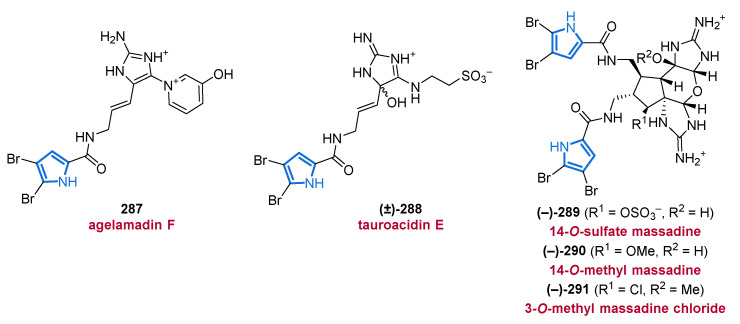
Unusual aminoimidazole pyrrole alkaloids **287**–**291** with compounds **289**–**291** incorporating a complex contiguous imidazole ring system.

The unique bromopyrrole alkaloids agelamadin F (**287**) and tauroacidin E (**288**) were isolated from an Okinawan marine sponge of the genus *Agelas* in 2015 (Figure 39) [64]. Compound **287** is the first example of a bromopyrrole alkaloid bearing an aminoimidazole moiety connected to a pyridinium ring. Tauroacidin E (**288**), possessing an uncommon taurine unit, was isolated as a racemic structure. Both halopyrroles **287** and **288** showed moderate activities against KB and human leukemia K562 cells with IC_50_ values in the range of 10 µg/mL [64].

The complex class of massadines was extended by the isolation of three new compounds **289**–**291** from a deep-water sponge of the genus *Axinella* in 2012 (Figure 39) [268]. The eight stereogenic centers of 14-*O*-sulfate massadine (**289**), 14-*O*-methyl massadine (**290**), and 3-*O*-methyl massadine chloride (**94**) were determined by NMR spectroscopy and optical rotation measurements. The generated data confirmed the absolute stereochemistry earlier defined by Köck [269] and Fusetani [270] for related massadines and was also consistent with the data from its enantioselective total synthesis [271]. While compounds **289**–**291** did not show any inhibitory activity against the neurodegenerative disease kinase targets CDK5/p25, CK1δ, and GSK3β, 3-*O*-methyl massadine chloride (**291**) exhibited antibacterial activity against several Gram-positive and -negative bacteria with IC_50_ values below 5 µM [268].

**Figure 40:**
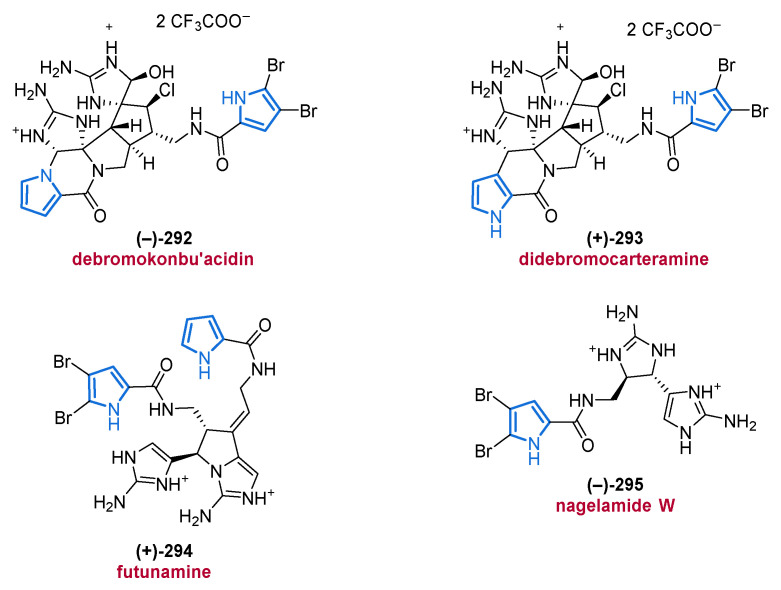
Biologically active bromopyrrole imidazole alkaloids **292**–**295** possessing unique structural motifs.

Three structurally similar alkaloids (**292**–**294**), possessing two or more contiguous ring systems were isolated from the sponge *Stylissa* aff. *carteri* in 2020 (Figure 40) [272]. The absolute stereochemistry of the two new hexacyclic analogs of palau’amine and styloguanidine, debromokonbu’acidin (**292**) and didebromocarteramine (**293**), was determined by comparison of experimental and theoretical ECD spectra. While compound **293** did not show any neuroprotective activity, compound **292** could reduce reactive oxygen species in neuroblastoma SY-SY5Y cells by 35% over a wide range of concentrations [272]. The stereochemistry of futunamine (**294**), featuring a new pyrrolo[1,2-*c*]imidazole core, was also deduced by ECD analyses. Furthermore, futunamine (**294**) showed neuroprotective effects at 10 µM. Unfortunately, none of the three new compounds **292**–**294** showed any cytotoxic activity [272].

Nagelamide W (**295**), the first monomeric bromopyrrole alkaloid bearing two aminoimidazole moieties, was isolated from a marine sponge *Agelas* sp. by the Kobayashi group in 2013 (Figure 40) [65]. The relative stereochemistry of **295** was elucidated by ROESY correlations and the natural product **295** exhibited inhibitory activity against *Candida albicans* with an IC_50_ value of 4 µg/mL [65].

**Figure 41:**
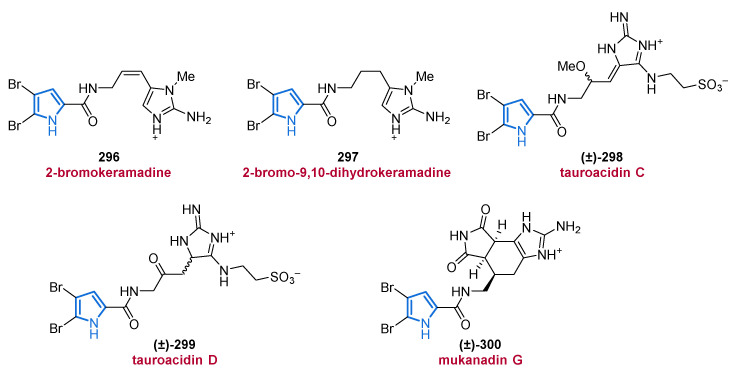
Related bromopyrrole alkaloids **296**–**300** and the antifungal mukanadin G (**300**) isolated from *Agelas* sp.

In 2014, five new bromopyrrole alkaloids (**296**–**300**) were isolated from an Okinawan marine sponge of the genus *Agelas* (Figure 41) [273]. Tauroacidin C (**298**), tauroacidin D (**299**), and mukanadin G (**300**) were isolated as racemic mixtures. However, the relative stereochemistry of mukanadin G (**300**) was established by ROESY and computational experiments. While compounds **296**–**298** did not show any antimicrobial activity, mukanadin G (**300**) exhibited good to moderate antifungal activity against the human-pathogenic yeast *Candida albicans* and the invasive pathogenic fungus *Cryptococcus neoformans* with IC_50_ values between 8 and 16 µM [273].

**Figure 42:**
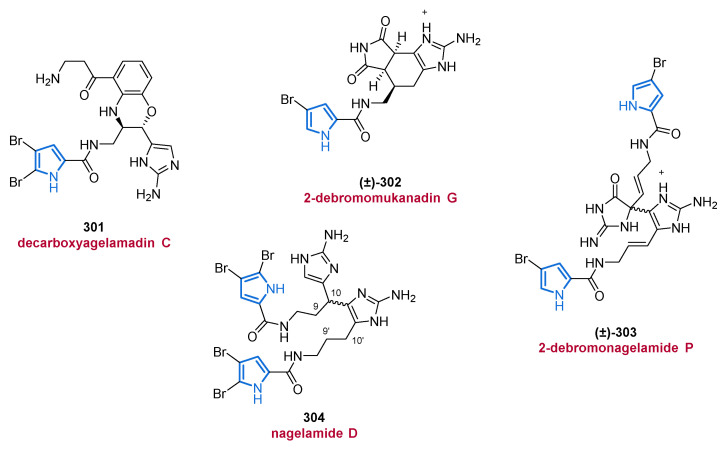
Oroidin-based bromopyrrole alkaloids **301**–**303** with nagelamide D (**304**) underwent a reevaluation in 2020.

In decarboxyagelamadin C (**301**), isolated from the sponge *Agelas sceptrum* in 2016, a rare morpholine core is located between the pyrrole and imidazole moiety with the relative and absolute stereochemistry being established by NMR and ECD spectroscopy (Figure 42) [274]. Unfortunately, compound **301** did not show any activity in cytotoxicity tests and in antimicrobial assays.

A new bromopyrrole alkaloid also incorporating a fused 6-membered ring, 2-debromomukanadin G (**302**), was isolated from another *Agelas* sp. alongside 2-debromonagelamide P (**303**) (Figure 42) [248]. While both substances **302** and **303** were isolated as racemates, the relative configuration of compound **302** could be deduced by comparison of its coupling constants with those from mukanadin G (**300**). Compound **303** showed moderate antimicrobial activity against *Trichophyton mentagrophytes* (IC_50_ value 32 µg/mL), whereas compound **302** exhibited moderate activity against *Cryptococcus neoformans* (IC_50_ value 32 µg/mL). However, no cytotoxicity was observed against human epidermoid carcinoma KB and murine lymphoma L1210 cells [248].

We also want to mention an inconsistency in the assigned structure for the structurally related nagelamide D (**304**), which was originally isolated in 2004 as a racemate by the Kobayashi group (Figure 42) [275]. Five years later, a total synthesis by the Lovely group [276] revealed that either the assigned structure or the reported NMR data of Kobayashi’s work was in error. However, no final evidence was given at this point. A recently published synthetical approach [277] of the same laboratory towards alkaloids belonging to the nagelamide class then corroborated the correctly proposed but incorrectly assigned structure by Kobayashi. In this case, crystallographic measurements [277] unequivocally demonstrated that the assignments for C9, C9′, C10′ as well as H9′a and H9′b were inadvertently switched in the original literature [275].

**Scheme 19:**
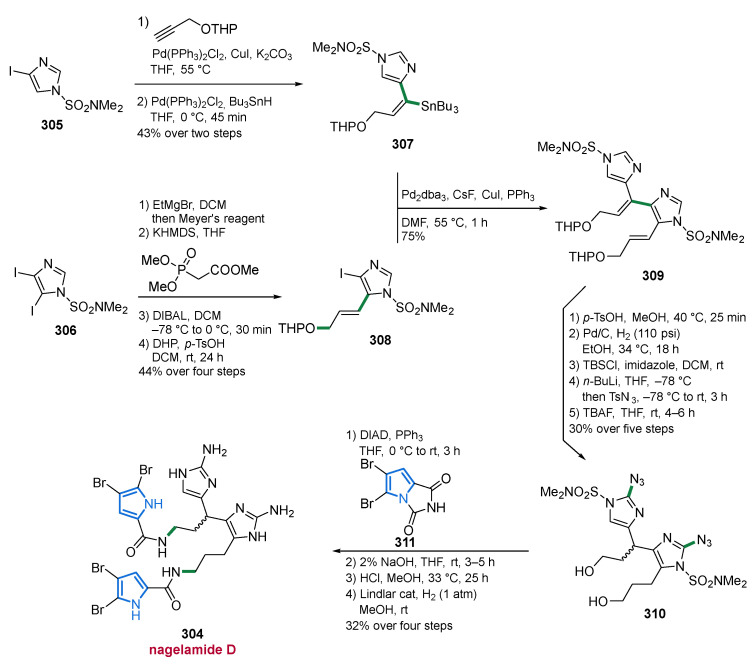
A total synthesis of nagelamide D published by the Lovely group led to the correct assignment of nagelamide D (**304**).

The Lovely group commenced their synthesis with the iodoimidazoles **305** and **306**, which were transformed into the corresponding coupling partners **307** and **308** over several steps, respectively. A Stille cross-coupling then delivered compound **309**. A reaction sequence involving several protection and deprotection reactions as well as the installation of the azide group via TsN_3_ furnished diol **310**. Replacing the alcohol functional groups by a pyrrole hydantoin **311**, hydrolysis, and deprotection of the corresponding urea followed by azide hydrogenation finally furnished nagelamide D (**304**) in 32% over four steps (Scheme 19) [277].

**Figure 43:**
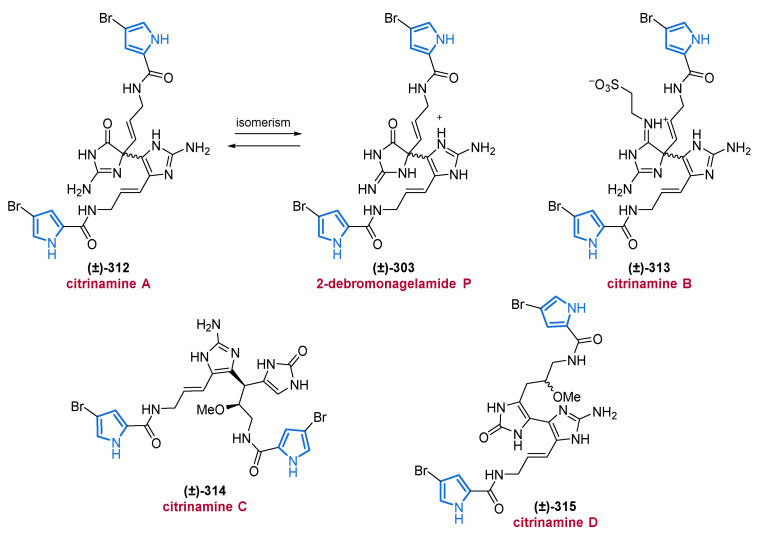
The dimeric bromopyrrole alkaloids citrinamines A–D (**312**–**315**).

A very similar class of compounds, the citrinamines A–D (**312**–**315**), were isolated from the Caribbean sponge *Agelas citrina* in 2015 by the Köck group (Figure 43) [63]. All four compounds **312**–**315** were isolated as racemic mixtures, with the relative configuration of citrinamine C (**314**) being elucidated with the aid of NOESY correlations and comparison of its NMR data with those of nagelamide B, a related congener isolated back in 2004 [275]. It should be mentioned that the same group isolated citrinamines C (**314**) and D (**315**) as a mixture, the separation of which by preparative chromatography failed. Citrinamines B–D (**313**–**315**) showed “considerable” inhibition zones in agar diffusion assays with *Mycobacterium phlei* (no values for the size of the inhibition zones were given). However, all compounds **312**–**315** exhibited no inhibition of cell proliferation of mouse fibroblasts [63]. Here, we would like to mention that the only structural difference between citrinamine A (**312**) and 2-debromonagelamide P (**303**) lies in the additional proton present in compound **303** (Figure 43). As the NMR spectra of both compounds **303** and **312** also appear to be identical, it is highly likely that both compounds **303** and **312** are in fact the same substance, although compound **303** was isolated as a salt and compound **312** as the free base.

**Figure 44:**
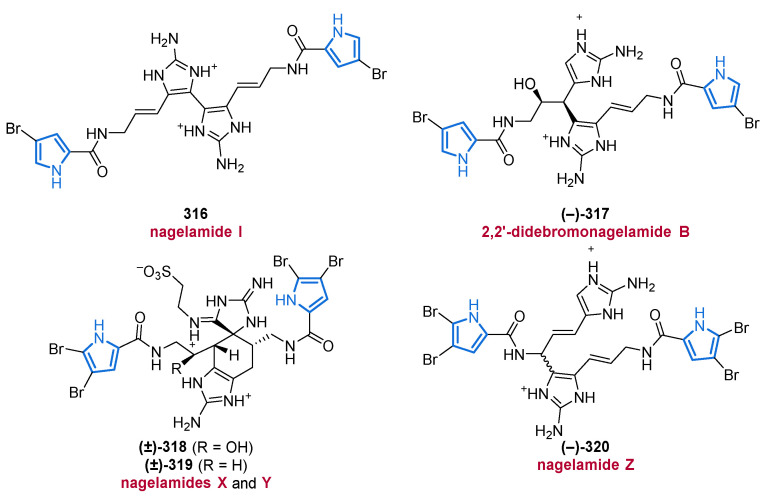
Five new family members (**316**–**320**) of the nagelamides from *Agelas* sp.

The known class of nagelamides was extended by nagelamides I (**316**) and 2,2′-didebromonagelamide B (**317**), isolated from a marine sponge *Agelas* sp. (Figure 44) [278]. The relative configuration of compound **317** could be deduced by extensive NMR-spectroscopic analysis but the absolute configuration remains unknown. Both compounds **316** and **317** did not show cytotoxicity against murine lymphoma L1210 and human epidermoid carcinoma KB cells in vitro [278].

Nagelamides X–Z (**318**–**320**) were isolated from a marine sponge of the genus *Agelas* in 2013 (Figure 44) [279]. Here, the nagelamides X (**318**) and Y (**319**) incorporate a unique tricyclic skeleton consisting of spiro-connected tetrahydrobenzaminoimidazole and aminoimidazolidine moieties. Compounds **318** and **319** were isolated as racemic mixtures with the relative configuration being determined by 2D NMR spectroscopy. Nagelamide Z (**320**) was isolated as an optically active molecule, but its absolute configuration remains unsolved. Nagelamides X–Z (**318**–**320**) displayed antimicrobial activities against several bacteria and fungi, with IC_50_ values partly being below 5 µg/mL [279].

**Figure 45:**
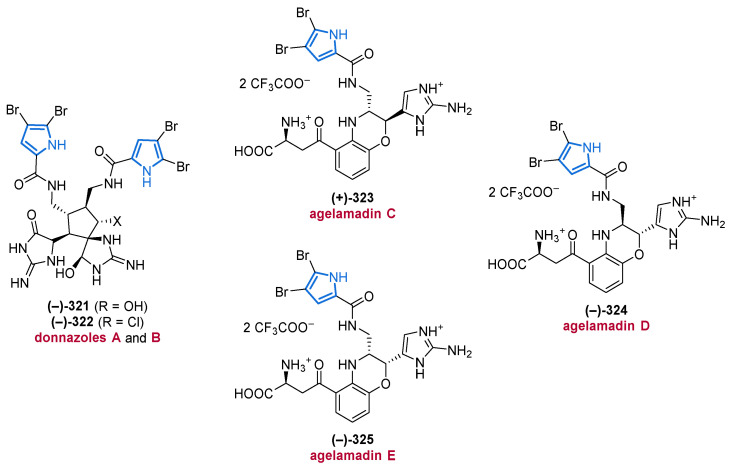
Donnazoles A (**321**) and B (**322**) from a marine sponge *Axinella donnani* and further agelamadins C–E (**323**–**325**).

In 2012, a new pair of dimeric pyrrole-aminoimidazole alkaloids, (−)-donnazoles A (**321**) and B (**322**), was isolated from the marine sponge *Axinella donnani* (Figure 45). The absolute configurations of **321** and **322** were determined via NOE correlations and ECD measurements [280].

The agelamadins C–E (**323**–**325**), isolated from a marine sponge of the genus *Agelas* in 2014, share the same flat structure but differ in their stereochemistries (Figure 45) [281]. The configurations of compounds **323**–**325** were elucidated by 2D NMR spectroscopy, ECD calculations, and by a phenylglycine methyl ester (PGME) method. To this end, (*R*)- and (*S*)-PGME are condensed with a carboxylic acid functionality, to generate amides enabling the determination of the absolute configuration by means of the diamagnetic anisotropic effect [282]. While agelamadin D (**324**) did not show any antimicrobial activity, agelamadins C (**323**) and E (**325**) displayed moderate inhibitory activity against the human pathogen *Cryptococcus neoformans* with IC_50_ values of 32 µg/mL each [281].

### 3.2. Annellated Pyrroles

Annellated pyrroles are prevalent in nature. For example, many well-known biologically active alkaloid families, including the lamellarins and indolizidins, as well as many stemona alkaloids, feature annellated pyrrole moieties [283,284,285].

**Figure 46:**
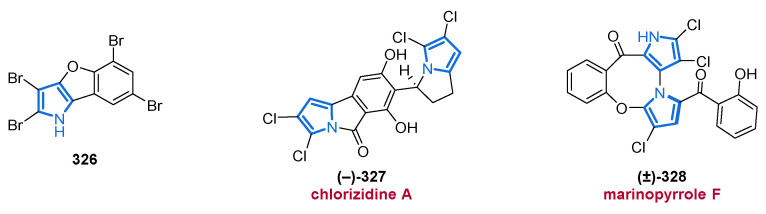
Annellated halopyrroles **326**–**328** derived from marine bacteria.

Between 2010 and 2012, the highly halogenated 5- and 8-ring annellated pyrroles **326**–**328** were isolated from marine bacteria (Figure 46). The *Pseudoalteromonas*-derived 2,3,5,7-tetrabromobenzofuro[3,2-*b*]pyrrole (**326**) displayed significant antimicrobial activity against methicillin-resistant *Staphylococcus aureus* (ATCC 43300, IC_50_ value of 1.93 µM ± 0.05 µM) [286].

The biologically active (−)-chlorizidine A (**327**) was isolated from a marine *Streptomyces* sp. and exhibited noteworthy activity in a human colon cancer cytotoxicity bioassay with IC_50_ values of 3.2–4.9 µM (Figure 46) [287]. Interestingly, the alkaloid **327** completely lost its activity when both phenolic functionalities were methylated. The authors also mentioned that a series of derivatives lacking the key 5*H*-pyrrolo[2,1-*a*]isoindol-5-one moiety led to inactivity, strongly suggesting its presence is indispensable for biological activity [287].

The structure of (±)-marinopyrrole F (**328**), isolated from a *Streptomyces* sp. in 2010, contains an unusual eight-membered ring (Figure 46) [250]. In contrast to its enantiopure metabolites, marinopyrroles C–E (**244**–**246**, see Figure 32), (±)-marinopyrrole F (**328**) was isolated in racemic form. With the help of chiral HPLC, the authors found out that enantioenriched **328** completely racemizes within 18 h, most probably caused by the fused ether ring lowering the barrier for atropisomerism. However, (±)-marinopyrrole F (**328**) was much less active against MRSA and HCT-116 (MIC_90_ value 3.1 µg/mL) compared to (−)-marinopyrrole C (**244**, MIC_90_ value 0.16 µg/mL) [250].

**Figure 47:**
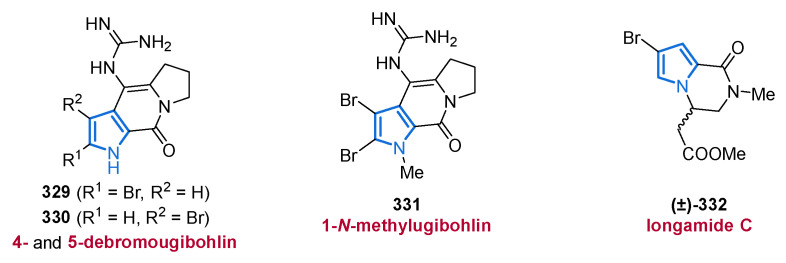
Structures of 2,3-annellated marine pyrrole alkaloids **329**–**332**.

In 2018, 4-debromougibohlin (**329**) and 5-debromougibohlin (**330**) were isolated from a marine sponge *Dictyonella* sp. by the Berlinck group (Figure 47). Unfortunately, both compounds did not show any proteasome inhibitory activity in a respective assay [264].

In 2019, a related halopyrrole alkaloid incorporating the carbamoylpyrrole-like core structure, 1-*N*-methylugibohlin (**331**), was isolated from the sea sponge *Agelas nemoechinata*, but did not show cytotoxic activity against K562, A549, HeLa, or HCT-116 cells in vitro (Figure 47) [262].

Longamide C (**332**), obtained from an organic extract of *Agelas nakamurai* in 2010, was isolated as a racemic mixture (Figure 47). However, ROESY correlations indicated a half chair conformation of the six-membered ring. Compound **332** did not show any promising antimicrobial or cytotoxic activity [234].

**Figure 48:**
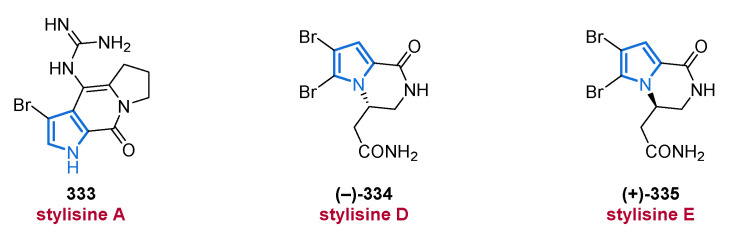
Stylisines A (**333**), D (**334**), and E (**335**) from the marine sponge *Stylissa massa*.

In 2017, the Lin group isolated stylisines A–F (**333**, **282**, **283**, **334**, **335**, **132**) from the marine sponge *Stylissa massa*, of which stylisine A, D, and E (**333**–**335**) feature an annellated bromopyrrole moiety (Figure 48) [133]. The absolute stereochemistry of compounds **334** and **335** was deduced from ECD experiments. However, no antibacterial activity was observed for all three compounds **333**–**335** [133]. One year later, 5-debromougibohlin (**330**, Figure 47) was isolated and erroneously presented as a “new” bromo alkaloid [264], since it has the same structure as stylisine A (**333**).

**Scheme 20:**
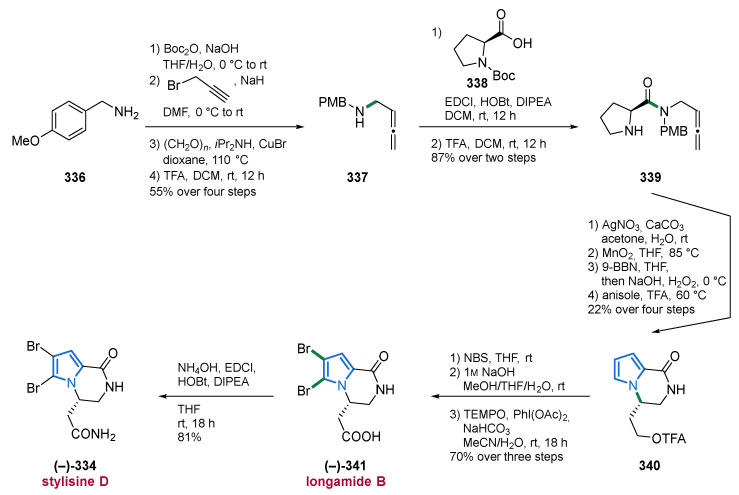
Synthesis of stylisine D (**334**) and intermediate longamide B (**341**) via a metal-catalyzed cyclisation of allene **339** in a stereoselective manner.

At this point, the stereoselective synthesis of (−)-stylisine D (**334**) reported by Petkovic and Savic in 2019 should be mentioned (Scheme 20) [288]. The synthesis commenced with an N-protection and propargylation followed by routine transformations to generate allene **337** in 55% yield over three steps. After installing Boc-l-proline (**338**) which furnished compound **339** possessing the right configuration, compound **340** was obtained over four steps under transfer of chirality. After bromination and hydrolysis, a final oxidation step delivered (−)-longamide B (**341**), another bromopyrrole isolated from the sponge *Stylissa massa*. (−)-Stylisine D (**334**) was obtained by amidation of the carboxylic group of (−)-longamide B (**341**).

**Figure 49:**
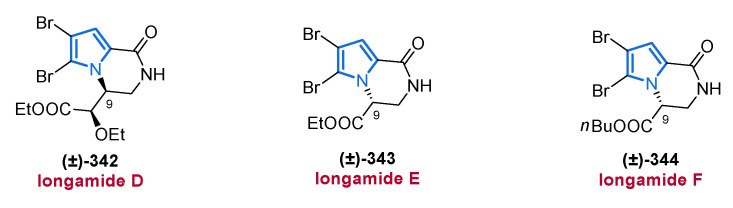
Longamides D–F (**342**–**344**) from the South China Sea sponge *Agelas* sp.

In 2016, the family of longamides was extended by the isolation of longamides D–F (**342**–**344**) from a marine sponge *Agelas* sp. (Figure 49) [66]. Compounds **342**–**344** were isolated as racemic mixtures which were separated into pure enantiomers. The absolute stereochemistry of **342**–**344** was then determined by chiral HPLC and ECD spectroscopy. In the *Caenorhabditis elegans* candidiasis model, metabolites (+)-**342**, (−)-**343** and (+)-**344** exhibited significant antifungal activity with survival rates around 50%, whereas the corresponding enantiomers (−)-**342**, (+)-**343** and (−)-**344** did not show any activity, strongly suggesting the absolute configuration at C-9 to have an appreciable effect [66].

**Figure 50:**
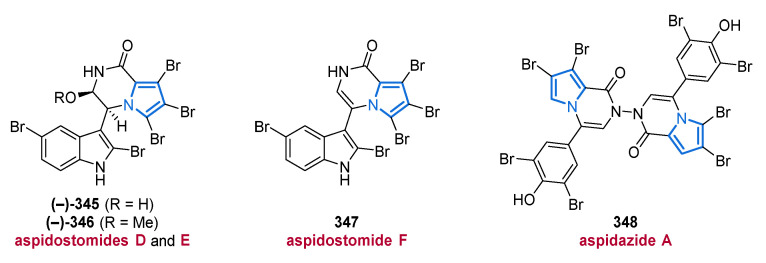
New aspidostomides D–F (**345**–**347**) and aspidazide A (**348**) from the patagonian bryozoan *Aspidostoma giganteum*.

In 2014, several structurally unique annellated halopyrroles **345**–**348** were isolated from the Patagonian bryozoan *Aspidostoma giganteum* by Palermo and co-workers (Figure 50) [239]. The absolute configurations of bromotryptophan-derived aspidostomides D (**345**) and E (**346**) were determined by a modification of Mosher’s method in combination with NOE correlations. While the elimination product of **345** and **346**, aspidostomide F (**347**), the N–N-linked dimeric aspidazide A (**348**) and compound **345** only exhibited moderate to weak cytotoxic activity against the 786-O human renal carcinoma cell line (IC_50_ values between 27.0 µM and >100 µM), aspidostomide E (**346**) proved active with an IC_50_ value of 7.8 µM [239].

**Figure 51:**
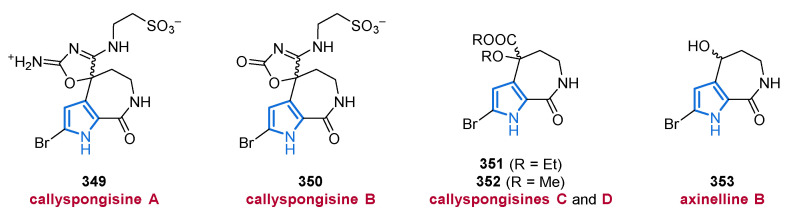
Callyspongisines A–D (**349**–**352**) and pyrrololactam **353** of which only compound **349** is speculated to be of natural origin.

In 2017, a new family of annellated halopyrroles, the callyspongisines, were isolated from the Great Australian Bight marine sponge *Callyspongia* sp. (CMB-01152) (Figure 51) [289]. In callyspongisines A (**349**), a very rare imino-oxazoline core is spirocyclic to a seven-membered ring contiguous to a pyrrole unit. Due to insufficient quantities of **349**–**352**, the stereochemistry could not be determined and the authors also mentioned that callyspongisines B–D (**350**–**352**) could be storage and handling artifacts of **349** instead of being of natural origin [289]. The potent kinase inhibitory activity observed in *Callyspongia* sp. was attributed to hymenialdisine, while compounds **349**–**352** did not show any cytotoxic activity against a range of prokaryotic, eukaryotic, and mammalian cell lines [289].

A related pyrrolactam alkaloid, axinelline B (**353**), was isolated from the *n*-BuOH extract of a marine sponge of the genus *Axinella* in 2017 (Figure 51). Unfortunately, the authors did not give any information about the stereochemistry or biological activity of compound **353 [131]**.

#### Annellated Pyrrole (Amino)-Imidazole Alkaloids

Several contiguous tetracyclic brominated pyrrole-imidazole alkaloids **354**–**356** were isolated or synthesized between 2016 and 2019.

**Figure 52:**
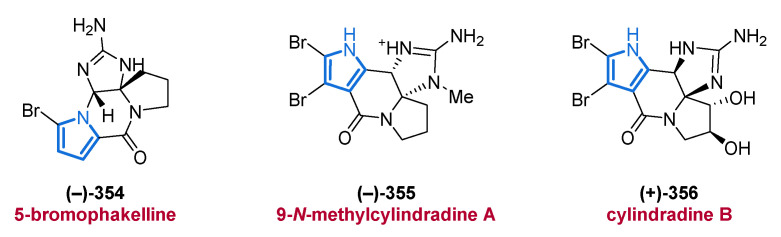
Brominated pyrrole-imidazole alkaloids **354**–**356** bearing guanidine units.

In 5-bromophakelline (**354**), isolated from an Indonesian marine sponge of the genus *Agelas*, the relative and absolute configuration was deduced with the help of NOESY correlations and X-ray crystallography (Figure 52). However, no antimicrobial activity against *Mycobacterium smegmatis* (NBRC 3207), a model organism for tuberculosis was observed [290].

Compound **355** was isolated from the sponge *Agelas nemoechinata* in 2019 (Figure 52). The relative and absolute configuration of 9-*N*-methylcylindradine A (**355**) was determined by NOESY correlations and by the comparison of its optical rotation with the known (+)-cylindradine A. Unfortunately, no cytotoxic activity against K562 and L-02 cell lines could be observed [262].

**Scheme 21:**
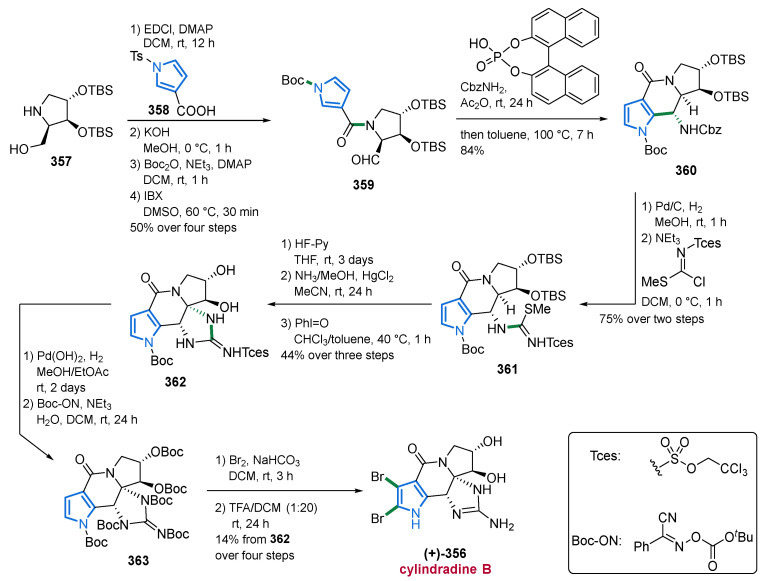
First total synthesis of (+)-cylindradine B (**356**) via key Pictet–Spengler reaction.

At this point, we would also like to mention the first total synthesis of (+)-cylindradine B (**356**) (Scheme 21) [291], which was isolated from the marine sponge *Axinella cylindratus* back in 2008 [292].

The authors commenced their synthesis with prolinol derivative **357** which was transformed with pyrrole **358** into the Pictet–Spengler precursor **359** over several steps. The Pictet–Spengler reaction then selectively gave compound **360** under addition of (±)-1,1′-binaphthyl-2,2′-diyl hydrogen phosphate. In the next steps, the guanidine group was attached via an isothiourea intermediate **361**, which reacted with NH_3_/MeOH furnishing compound **362**. After changing the protective groups, the Boc-protected pyrrole **363** was brominated by using bromine and a final deprotection by applying TFA furnished (+)-cylindradine B (**356**) in 14% yield over four steps (Scheme 21) [291].

**Figure 53:**
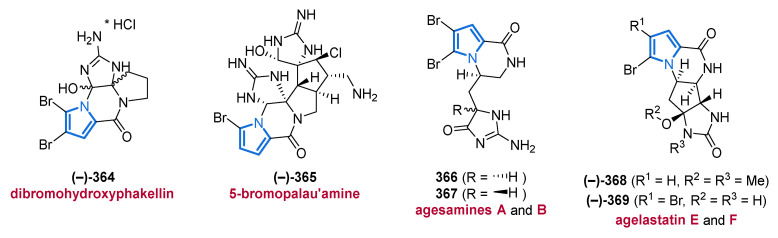
Structurally complex annellated bromopyrroles **364**–**369** isolated from *Dyctionella* sp. or *Agelas* sp.

In 2010, a compound very similar to **354**, dibromohydroxyphakellin (**364**), was isolated from *Agelas linnaei* and represents the first described 12-OH analog of the phakellin family (Figure 53) [234]. By comparison of its optical rotation data with those of related compounds, it was assumed that dibromohydroxyphakellin (**364**) was isolated as a scalemic mixture. No cytotoxicity was observed against the murine L1578Y mouse lymphoma cell line [234].

In 5-bromopalau’amine (**365**), isolated from *Dictyonella* sp. (marine sponge), the relative configuration of the eight stereogenic centers was determined by ROESY correlations [264] and was in accordance with the data reported for the revised structure of palau’amine (Figure 53) [293]. Compound **365** displayed proteasome inhibition activity with an IC_50_ value of 9.2 µM ± 3.2 µM, whereas the debrominated analog, palau’amine, was fourfold more active. Due to these data, the authors mentioned that both, bromination and the position of the bromine substituent in the pyrrole moiety seem to significantly influence the ability to inhibit the 20S yeast proteasome [264].

In 2019, a new class of annellated bromopyrroles, the agesamines A (**366**) and B (**367**), were isolated as an inseparable epimeric mixture from an Indonesian sponge of the genus *Agelas* (Figure 53). The absolute configuration of both compounds **366** and **367** was elucidated by ECD measurements [294].

The related agelastatins E (**368**) and F (**369**) were isolated from the marine sponge *Agelas dendromorpha* in 2010 (Figure 53) [295]. The relative configuration of both compounds **368** and **369** was determined by NOESY correlations and by comparison to the known congener agelastatin A. As agelastatin A is a highly cytotoxic compound, agelastatins E (**368**) and F (**369**) were screened for cytotoxicity against the human KB cell line. Unfortunately, both compounds **368** and **369** lacked significant activity [295].

**Scheme 22:**
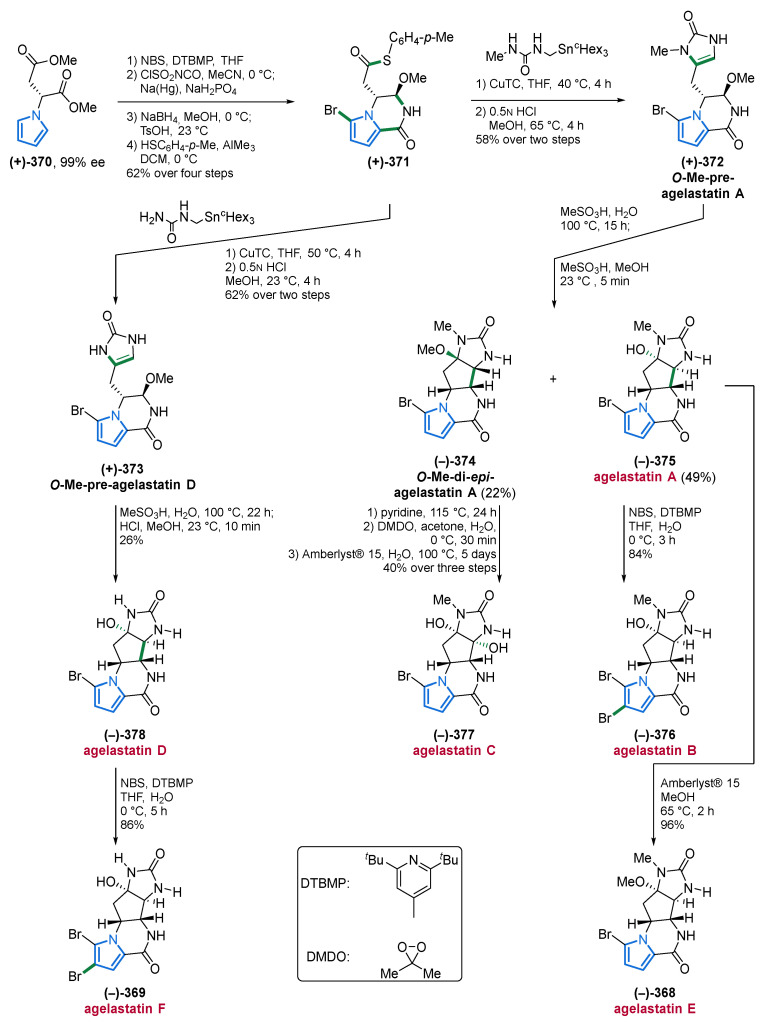
Enantioselective synthesis of all known (−)-agelastatins, including the first total synthesis of agelastatins C–F (**377**, **378**, **368**, **369**).

Concerning the agelastatin family, the total synthesis of agelastatins A–F (**375**–**378**, **368**, **369**), published by the Movassaghi group in 2010, should be mentioned (Scheme 22) [296]. The synthesis commenced with the known pyrrole **370**, which was converted into the annellated pyrrole **371** in 62% yield over four steps. After the addition of a stannylmethylurea in the presence of Liebeskind’s CuTC reagent and treatment with methanolic HCl, (+)-*O*-Me-pre-agelastatin A (**372**) was obtained. Subsequent heating in aqueous methanesulfonic acid then furnished the natural product agelastatin A (**375**) in 49% yield as well as a side product (**374**). Bromination or OH-methylation of agelastatin A (**375**) gave agelastatin B (**376**) or E (**368**), respectively. Moreover, (−)-*O*-Me-di-*epi*-agelastatin A (**374**) could be further converted to agelastatin C (**377**) by an elimination/epoxidation/aqueous epimerization sequence. By reacting the former intermediate **371** with a stannylurea, agelastatins D (**378**) and F (**369**) could be synthesized in a similar way (Scheme 22) [296].

**Figure 54:**
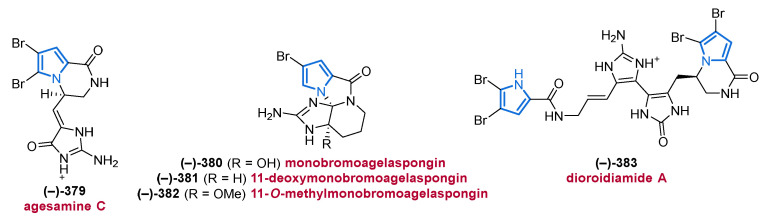
Structurally diverse bromopyrrole alkaloids **379**–**383** isolated from *Agelas oroides*.

In 2020, a new member of the agesamine family, agesamine C (**379**), could be isolated from the sea sponge *Agelas oroides* collected off the Tel Aviv coast (Figure 54). The relative and absolute configuration of the bicyclic moiety in **379** was deduced by comparison of its *J*-values with those of agesamines A (**366**) and B (**367**) [237].

Monobromoagelaspongin (**380**) was first isolated from the sponge *Agelas oroides* as a racemic mixture in 2017 and no information was given on the relative configuration or its biological activities [297]. However, in 2020, the relative and absolute configuration could be determined alongside the isolation of further bromopyrroles (Figure 54) [237].

The same sponge also delivered the agelaspongin analogs **381** and **382**, the relative and absolute configurations of which were either determined by NOESY data combined with ECD spectroscopy or by comparison of its chiroptical properties with those of model compounds (Figure 54) [237]. The sponge also was the source of a new compound, named dioroidamide A (**383**). Compound **383** presents a negative specific rotation value which is also the case for many other structurally related marine alkaloids, and based on their shared biosynthesis, the authors assumed that **383** should possess the same absolute configuration as depicted in Figure 54 [237]. With the isolated natural products **379**–**383** itself, no biological tests were performed. However, as the antimicrobial and antibacterial activity of the sponge extract was attributed to other natural products contained, compounds **379**–**383** have not been found to show any promising activities so far [237].

**Figure 55:**
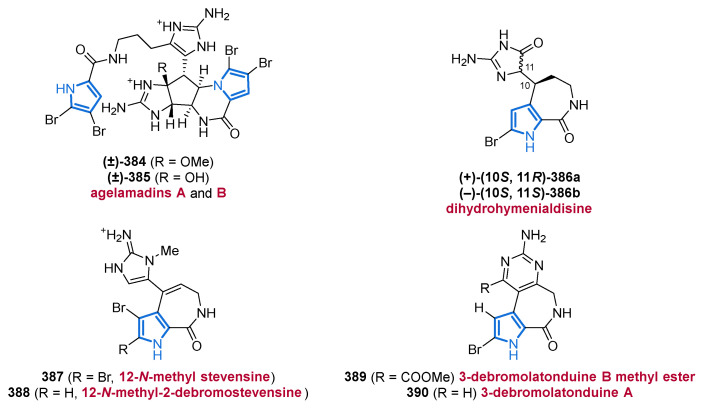
Annellated bromopyrroles **384**–**390** from different marine sponges.

In 2014, two structurally unique dimeric bromopyrroles, named agelamadins A (**384**) and B (**385**), were isolated from a sponge of the genus *Agelas* by the Kobayashi group [298]. Both compounds **384** and **385** were isolated as racemic mixtures, with their relative configurations determined by ROESY correlations (Figure 55). Agelamadins A (**384**) and B (**385**) showed antimicrobial activity against several Gram-positive species with IC_50_ values ranging between 4 µM and 16 µM. However, no cytotoxicity was observed against human murine lymphoma L1210 cells and human epidermoid carcinoma cells in vitro [298].

Two new bromopyrroles **368a** and **368b**, annellated by a seven-membered ring and structurally related to hymenialdisine, were isolated from the marine sponge *Cymbastela cantharella* in 2011 (Figure 55) [132]. The absolute structure of (+)-dihydrohymenialdisine (**368a**) was unequivocally determined by X-ray crystallography, whereas the absolute configuration of (−)-dihydrohymenialdisine (**368b**) could not be deduced. Since the corresponding lead structure, hymenialdisine, is active against the kinase PLK-1, both substances **368a** and **368b** were also tested for PLK-1 inhibition but did not show any activity. Apparently, the conjugation of hymenialdisine through the C-10/C-11 double bond (which is saturated in **368a** and **368b**) is indispensable for its strong activity on a wide range of cyclin-dependent kinases [132].

The structurally similar compounds **387**–**390** were isolated from a marine sponge of the genus *Stylissa* in 2012 (Figure 55) [299]. While 12-*N*-methylstevensine (**387**) displayed strong cytotoxic activity against L5178Y mouse lymphoma cells with an EC_50_ value of 3.5 µg/mL, 12-*N*-methyl-2-debromostevensine (**388**), 3-debromolatonduine B methyl ester (**389**), and 3-debromolatonduine A (**390**) only exhibited weak activity (no values given). These data suggest that the presence or absence of bromine atoms significantly influences the antiproliferative activity [299].

**Scheme 23:**
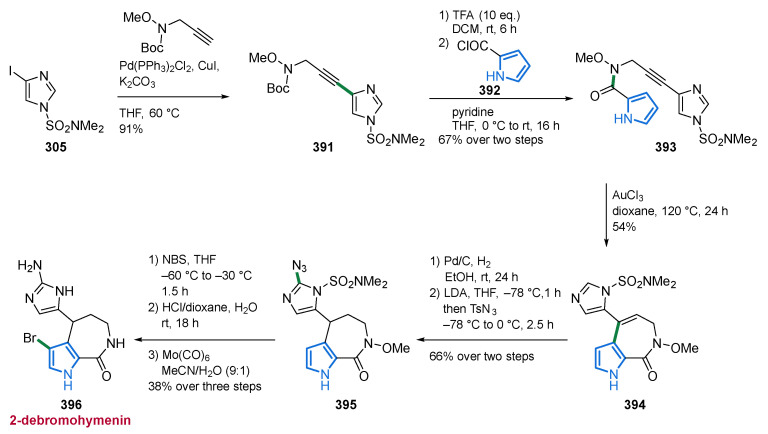
Total synthesis of 2-debromohymenin (**396**) via a key gold-catalyzed alkyne hydroarylation.

At this stage, it should also be mentioned the recently published total synthesis of the related pyrroloazepinone-containing alkaloid 2-debromohymenin (**396**) (Scheme 23) [300]. First, the commercially available 4-iodoimidazole **305** was transformed into alkyne **391** by a Sonogashira reaction. Subsequent deprotection and reaction with pyrrolecarbonyl chloride **392** furnished compound **393**. An intramolecular gold-catalyzed alkyne hydroarylation then resulted in the formation of the core pyrroloazepinone moiety in **394**. Subsequent hydrogenation followed by the installation of an azide group generated azido derivative **395**. Bromination using NBS, removal of the sulfonyl urea, and final conversion of the azide to an amine group as well as removing the N-OMe group at the same time using Mo(CO)_6_, furnished 2-debromohymenin (**396**) [300].

### 3.3. Sceptrins

The members of the exceptional family of the sceptrin alkaloids are characterized by their cyclobutane ring which is constructed by the dimerization of oroidin and its derivatives [301]. They are known to exhibit a broad range of biological activities, such as anticancer, antifungal, antibacterial, and anti-inflammatory [226,302,303,304]. Sceptrin was isolated and fully elucidated in 1981 by Faulkner and co-workers who also established its absolute configuration [224]. Many sceptrin derivatives have been isolated since.

**Figure 56:**
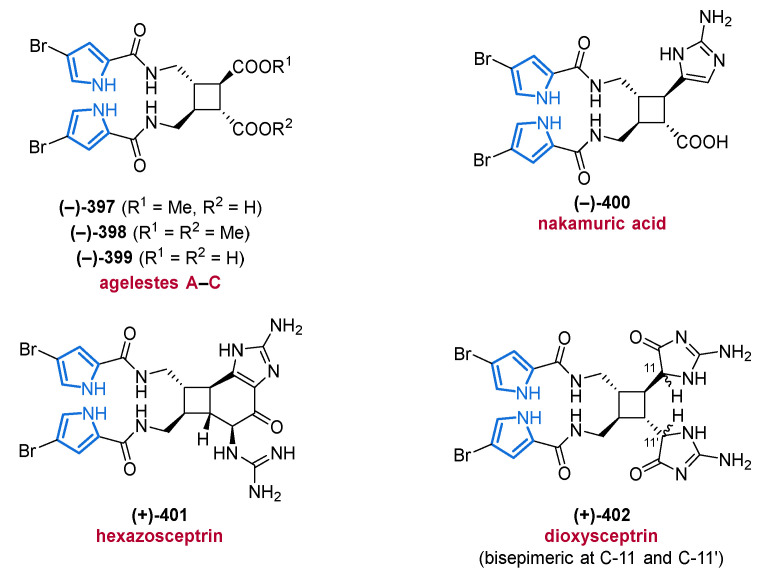
Several different substituted bromopyrroles **397**–**402** belonging to the sceptrin-family.

In 2017, agelestes A (**397**) and B (**398**) were isolated from a South China sponge of the genus *Agelas* (Figure 56) [305]. Although nakamuric acid (**400**) was already isolated in 1999 [306], the authors revealed its absolute configuration for the first time (Figure 56) [306]. The same sponge *Agelas* sp. also led to the isolation of hexazosceptrin (**401**), bearing a rare cyclohexane-fused-cyclobutane skeleton. All relative and absolute configurations were determined by extensive spectroscopic analyses and ECD. All four compounds **397**, **398**, **400**, and **401** displayed moderate antimicrobial activity (MIC values ranging between 16 µg/mL and 32 µg/mL) [305].

One year later, two sceptrin derivatives, ageleste C (**399**) and dioxysceptrin (**402**) were isolated from the marine sponge *Agelas Kosrae* (Figure 56) [307]. The relative and absolute configurations of compounds **399** and **402** were determined by ROESY correlations and by ECD spectroscopy. However, due to the absence of reliable ROESY correlations, the configuration at C-11 and C-11′ could not be determined. Ageleste C (**399**) and the bisepimeric dioxysceptrin (**402**) showed good to moderate anti-proliferative activity against six cancer cell lines (IC_50_ values ranging between 7.92 µM and > 50 µM), however, only compound **399** displayed moderate inhibition of *Candida albicans*-derived isocitrate lyase (IC_50_ value 22.09 µM), a key enzyme in microbial metabolism [307].

**Figure 57:**
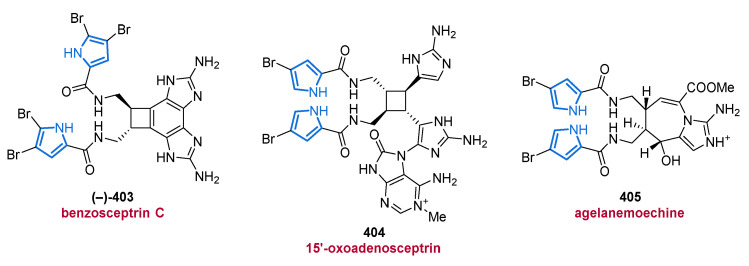
Further sceptrins **403** and **404** together with the congener agelanemoechine (**405**).

In 2010, the New Caledonian sponge *Agelas dendromorpha* led to the isolation of benzosceptrin C (**403**) featuring a rare benzocyclobutane moiety (Figure 57). Unfortunately, no cytotoxicity against the KB cell line was observed [295].

In 2016, the Köck group investigated the tropical sponge *Agelas sceptrum* which led to the isolation of 15′-oxoadenosceptrin (**404**), a hybrid PIA incorporating an adenine moiety. Unfortunately, no cytotoxic or antimicrobial activity was observed for compound **404** (Figure 57) [274].

In 2019, a unique alkaloid **405** bearing an imidazo [1,5-*a*] azepine nucleus was isolated from the marine sponge *Agelas nemoechinata*, with its relative and absolute configuration being determined by NOESY correlations and ECD spectroscopy, respectively. Agelanemoechine (**405**) showed potent pro-angiogenic activity in zebrafish (effect equivalent to the established Danhong injection as a positive control, Figure 57) [308].

**Scheme 24:**
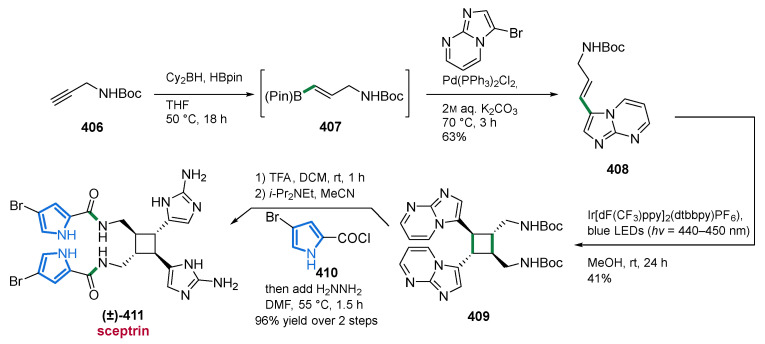
A four-step synthesis of sceptrin (**411**), including a photochemical intermolecular [2 + 2] dimerization as the key step.

At this point, the very recently published total synthesis of the dimeric PIA sceptrin (**411**) should be mentioned, which enables direct entry to this class of biologically active metabolites (Scheme 24) [309]. Astonishingly, sceptrin (**411**) was synthesized in only four steps by applying a photochemical intermolecular [2+2] dimerization of compound **408**. The authors synthesized building block **408** by initial hydroboration of protected propargylamine **406** to give pinacol ester **407** which then underwent a Suzuki–Miyaura cross-coupling with 3-bromoimidazopyrimidine. The key dimerization was carried out with blue LEDs in the presence of an iridium catalyst and provided the *all-trans* dimer **409** in 41% yield. Completion of the synthesis included acid-promoted deprotection, installation of the bromopyrrole unit **410**, and hydrazine-based conversion of the guanidine unit to an imidazole moiety in one pot [309].

Although there have been successful approaches towards sceptrin (**411**) since 2004, this new approach gives synthetic access to the sceptrin family in a minimum number of steps compared to the 11–25 steps required before [310,311,312]. It should also be mentioned that the synthetic work of the Chen laboratory in 2014 led to the revision of the absolute stereochemistry of many sceptrin-based natural products and of sceptrin (**411**) itself [312]. For more than 30 years, many groups have based their stereochemical results on the comparison with the incorrectly determined absolute configuration of sceptrin (**411**) from a publication of 1981 [224]. Hence, careful reading and checking are strongly recommended to avoid confusion.

## 4. Miscellaneous

Among the known marine pyrroles, there are also complex architectural frameworks containing macrocyclic ring systems, not only one or more sugar residues, but also multiple amide bonds forming peptides or even cyclopeptides. Therefore, in the following section, structures and classes are presented that could not be classified in the previous chapters due to their mostly complex and intriguing scaffolds.

In 2019, a scalarane sesterterpenoid featuring a 6/6/6/6/5-pentacyclic core was isolated from the sponge *Scalarispongia* sp. The fused pyrrole **412** represents the first pyrrole derivative in the rare class of N-heterocyclic scalaranes (Figure 35). MNP **412** was found to show moderate inhibition against six human cancer cell lines in bioactivity assays (GI_50_ values ranging between 14.9 µM and 26.2 µM) [313].

**Figure 58:**
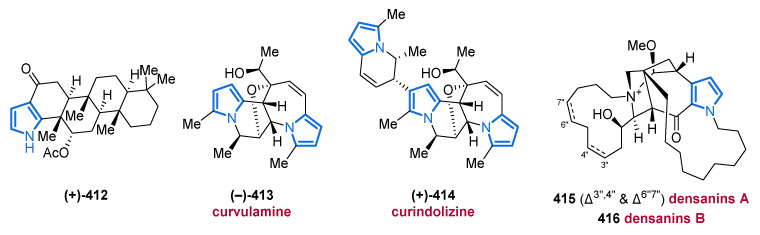
Polycyclic, complex molecular frameworks of condensed pyrrole MNPs **412**–**416**.

The bispyrrole curvulamine (**413**) originates from the fungus *Curvularia* sp. IFB-Z10, produced in a symbiontic way with the host, the White Croaker (*Argyrosomus argentatus*) (Figure 58) [314]. In the course of structure elucidation and determining the crystal structure of the unprecedented framework of curvulamine A, the authors also made efforts to elucidate the biosynthetic pathway using NMR-based ^13^C labeling experiments. Curvulamine (**413**) possesses antibacterial activity in the sub-micromolar range [314], whereas the biogenetic related trispyrrole curindolizine (**414**) lacks these bioactivities. However, anti-inflammatory activities in lipopolyssacharide (LPS)-stimulated RAW 264.7 macrophages (IC_50_ = 5.31 µM ± 0.21 µM) could be observed. Surprisingly, as a by-product of reisolating curvulamine (**413**), curindolizine (**414**) was discovered in 2016, two years after the initial isolation of curvulamine (**413**) from the same fungus (Figure 58). On this basis, it is also assumed that curindolizine (**414**) represents the product of an in vivo Michael addition of the metabolites curvulamine (**413**) and the elimination product derived from procuramine (**125**) (cf. Figure 16) [125].

Another complex polycyclic scaffold is displayed by the densanins A (**415**) and B (**416**) (Figure 58) [315]. After extensive NMR studies, including the application of the Mosher ester method, the 3D structure featuring seven stereogenic centers and a 1-azabicyclo[3.2.1] octane core was determined to be biosynthetically derived from 3-alkylpyridines. The hexacyclic diamines **415** and **416**, isolated from the sponge *Haliclona densaspicula* in 2012, showed no cytotoxicity but promising inhibition of the NO production in LPS-induced BV2 microglial cells (IC_50_ values of 1.05 µM and 2.14 µM, respectively) [315].

**Scheme 25:**
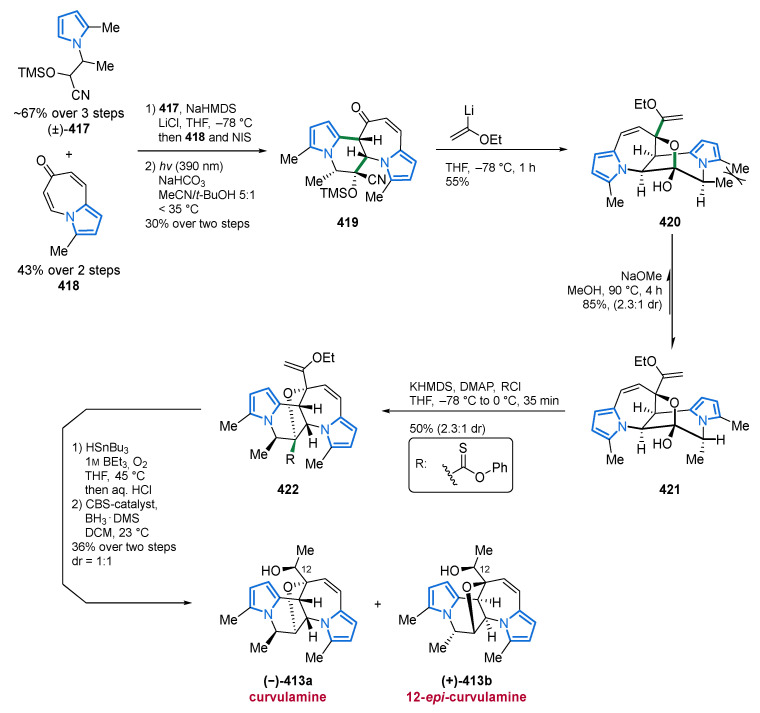
A linear ten-step sequence yielding the natural bispyrrole (−)-curvulamine **413a**.

Their promising bioactivity and challenging structures have inspired organic chemists ever since to develop a successful total synthesis of these MNPs [316,317,318]. The group of Maimone published the first successful synthesis of (−)-curvulamine (**413a**) in 2012, which was only feasible after extensive reconnaissance and several failures (Scheme 25) [319,320]. Starting from commercially available chemicals, they employed a feasible 10 step sequence to (−)-curvulamine (**413a**). The first key step was the coupling of racemic cyanohydrin **417**, as a masked acyl anion, with pyrroloazepinone **418**. This regioselective process was mediated by NaHMDS, followed by quenching the resulting enolate with NIS. After extensive investigation, the iodide was found to undergo cyclization under simple irradiation conditions in MeOH.

In this way, compound **419** was prepared in a 30% yield over two steps. After addition of lithiated ethyl vinyl ether, subsequent epimerization to the favored diastereomer **421**, and activation of the secondary alcohol with ClCSOPh, the thiocarbonate epimers (1*R*/1*S*)-**422** could be separated. The desired isomer **422** was reduced by deoxygenation and hydrolysis of the enol ether. The final step involves a diastereoselective reduction of the racemic ketone under CBS reduction conditions, yielding a 1:1 epimeric mixture of alcohols **413a** and **413b** that was readily separated into the enantiopure MNPs (Scheme 25) [319].

Syntheses such as the one shown by Maimone et al. play a significant in the development of potential active pharmaceutical ingredients as marine organisms often cannot be easily cultivated for mass production [319]. Further synthetic attempts, e.g., to prepare densanins, were undertaken by Yang and co-workers in 2016, whereas only the BCD tricyclic core could be achieved [321].

### 4.1. Pyrroloiminoquinone and Related Analogs

**Figure 59:**
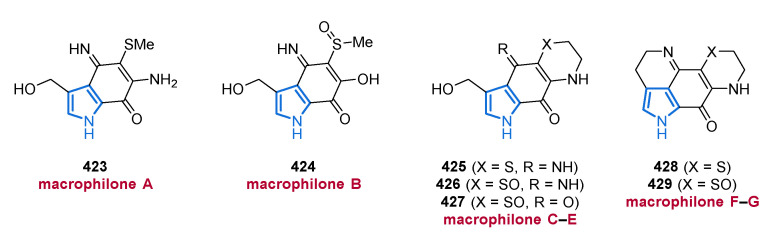
Members of the macrophilones group **423**–**429**.

The pyrroloiminoquinones feature a central core in a broad variety of MNPs, divided into subclasses of iso-/batzellins, damirons, discorhabdins, epinardins, makaluvamines, prianosins, tsitsikammamins, wakayins, and veiutamines [322,323,324]. Among them, a new subclass of the heteroatom-rich macrophilones was established in 2017. Macrophilone A (**423**), isolated from the *Macrorhynchia philippina*, represents a rare example of the underexplored group of hydroids (Figure 59) [325]. Macrophilone A (**423**), together with a synthetic derivative prepared in the same study, was able to block the conjugation cascade of small ubiquitin-like modifier (SUMO). The SUMO conjugation to protein substrates occurs through an enzymatic cascade and is critical for the regulation of various cellular processes. It is often disrupted in diseases, including cancer, resulting in the disturbance of the protein balance [325].

Once more, the group of Gustafson and co-workers published the isolation of six further macrophilones B–G (**424**–**429**) from the same source one year later (Figure 59) [326]. Just as its related congener macrophilone A (**423**), compounds **424**–**429** showed moderate to weak inhibition effects of SUMO conjugation cascade (IC_50_ values ranging between 11.9 µM and >100 µM). Furthermore, they exhibited significant toxicity against several cancer cell lines (no values given) [326].

**Scheme 26:**
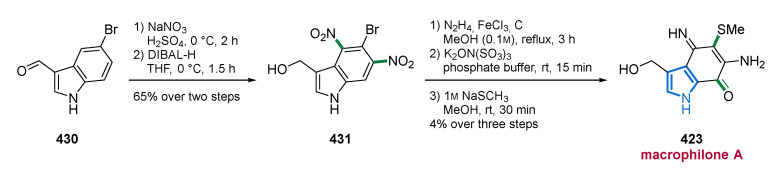
Synthesis of macrophilone A (**423**) in a linear sequence of 5 total steps.

To investigate their bioactivity potential, the first isolation of macrophilone A (**423**) was accompanied by its synthesis [325]. The authors started their ingeniously short approach from commercial formylindole **430**, which was nitrated and the aldehyde functionality reduced subsequently to furnish compound **431**. Oxidation by Fremy’s salt yielded the iminoquinone, which, after the introduction of the thioether group by sodium methanethiolate, furnished the natural product **423** in just 4% yield over three steps (Scheme 26) [325].

**Figure 60:**
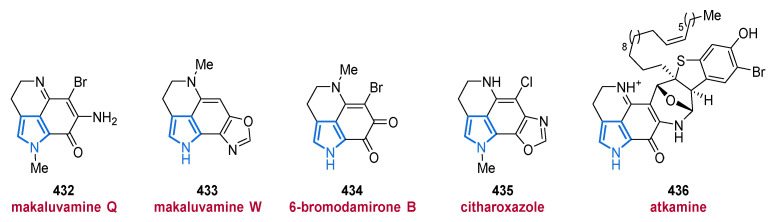
Pyrroloiminoquinones and related derivatives **432**–**436** isolated from natural sources, which share a similar biosynthetic pathway.

In 2019, makaluvamine Q (**432**) was discovered, marking the first time a makaluvamine derivative was isolated from a marine *Tsitsikamma* sponge within the Latrunculiidae family (Figure 60). Besides the shown DNA intercalation and topoisomerase I inhibition (27% inhibition of DNA nicking), makaluvamine Q (**432**) was found to be most active against HeLa cells in cell viability assays (14.7% ± 0.5% metabolic activity at 10 µM). In addition, the authors showed possible biosynthetic relationships between the isolated subclasses [327].

Only a few months later, the Keyzers lab isolated makaluvamine W (**433**) and 6-bromodamirone B (**434**) from the sponge *Strongylodesma tongaensis* (Figure 60). Both isolated pyrrole derivatives **433** and **434** lacked cytotoxic activity against the leukemia cell line HL-60, highlighting the importance of an iminoquinone scaffold in bioactivity considerations [328].

The benzoxazole moiety in makaluvamine W (**433**) is also found in citharoxazole (**435**), isolated from the sponge *Latrunculia (Biannulata) citharistae* in 2011 (Figure 60). The latter compound represented the first oxazole derivative in this family at that time [329].

In 2013, the Hamann laboratory isolated a complex heptacyclic pyrroloiminoquinone **436** containing seven stereogenic centers together with five different heterocycles (Figure 60). The TFA salt of atkamine (**436**) was isolated from the sponge *Latrunculia* sp. The structure elucidation of this complex framework was guided by spectroscopic methods, including ECD spectroscopy to analyze the absolute configuration. Furthermore, preparative olefin metathesis was used to localize the (*E*)-configured double bond [330].

**Scheme 27:**
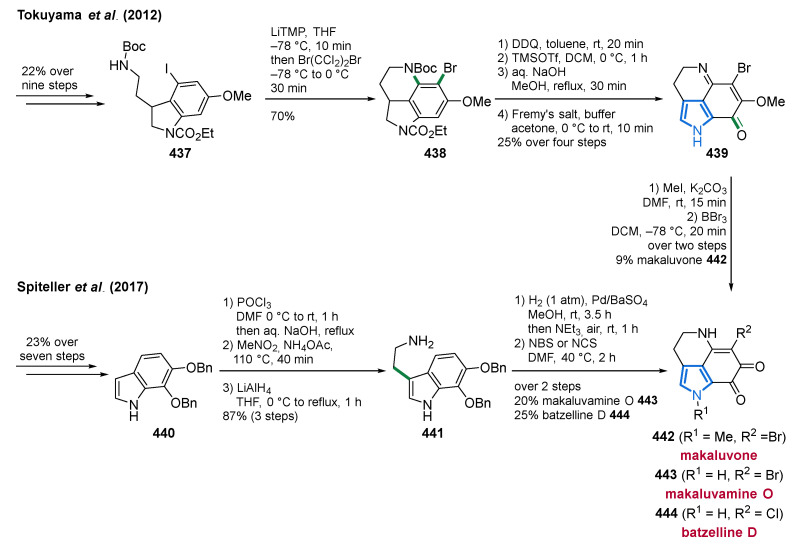
Two different routes which target pyrroloquinolines **442**, **443**, and **444**. The first route favors the formation of the quinoline followed by pyrrole aromatization, while the second one uses a biomimetic approach with a late-stage quinoline ring closure.

Due to their promising bioactivity, a large number of synthetic studies have been conducted on these pyrrole alkaloids (e.g., makaluvamines [331,332], damirones [333,334], batzellines [335,336]). The first synthesis of makaluvone was completed by the Tokuyama group in 2012 [337]. Starting with 4-methoxy-2-nitroaniline and using a procedure reported by the Buchwald group [338], the 4-iodoindoline **437** was prepared in a 22% yield over nine steps. Subsequent construction of the quinoline scaffold using a benzyne intermediate generated by LiTMP and trapping of the carbanion by a bromine donor resulted in the formation of tricyclic system **438**. DDQ-oxidation to form the indole, removal of the N-protecting groups, and oxidation of the aromatic core yielded the iminoquinone **439**. The last two steps included the methylation of the pyrrole nitrogen, methyl ether cleavage, and isomerization to makaluvone **442** (Scheme 27) [337].

A shorter and more efficient synthetic sequence to several aminoquinolines was reported five years later by the Spiteller group (Scheme 27) [339]. Using vanillin as starting material, the indole **440** was prepared in 23% yield over seven steps. Vilsmeier formylation, Henry reaction, and LiAlH_4_ reduction of the nitroolefin then furnished the tryptamine **441**. Removal of the benzylic protecting groups under hydrogenolytic conditions, oxidation of the prepared hydroquinone followed by biomimetic intramolecular Michael addition and aerobic reoxidation then gave the targeted pyrroloquinoline. The last step involved halogenation to obtain makaluvamine O (**443**) and batzelline D (**444**), respectively [339].

**Figure 61:**
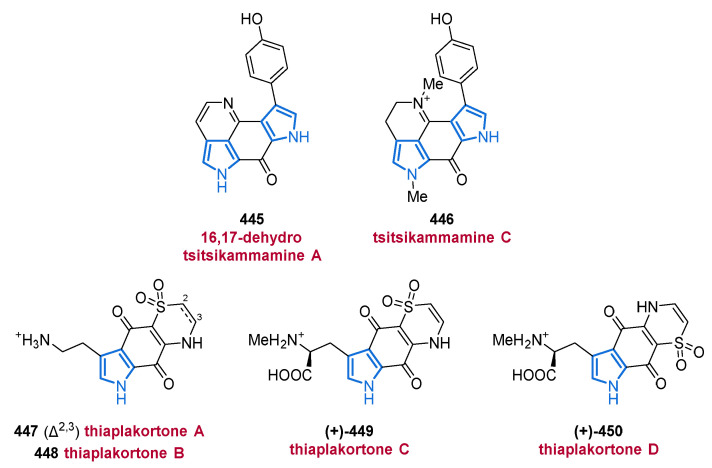
Pyrroloiminoquinones **445** and **446** as well as pyrroloquinones **447**–**450**.

A new member of the tsitsikammamines, namely 16,17-dehydrotsitsikammamine A (**445**), was identified from the Antarctic sponge *Latrunculia biformis* in 2018 (Figure 61). The crude extract of bis-pyrroloiminoquinone **445** showed promising anticancer activity against seven cancer cell lines (inhibition percentage >90% each at 200 µg/mL) [340].

The new tsitsikammamine C (**446**) was isolated as the TFA-salt from *Zyzzya* sp. in 2012 and represents the 18-methyl derivative of tsitsikammamine B (Figure 61). In biological assays, a potent growth inhibition of *Plasmodium falciparum* chloroquine-sensitive (3D7, IC_50_ value of 13 nM) and chloroquine-resistant (Dd2, IC_50_ value of 18 nM) cell lines was observed [341].

Thiazine-derived metabolites were discovered in the Australian marine sponge *Plakortis lita* in 2013 and given the names thiaplakortones A–D (**447**–**450**) (Figure 61) [342]. The structures were determined by using NMR and MS analytics as well as comparing chiroptical data to literature values to confirm the absolute configuration of the 2-methylaminopropanoic acid side chain of thiaplakortone C (**449**) and D (**450**). This substituent also suggests the biosynthesis from l-tryptophan and cysteine to yield the tricyclic framework. As the aforementioned tsitsikammamine C (**446**), all tested thiaplakortones **447**–**450** display significant antimalarial activity against chloroquine-sensitive (3D7, IC_50_ values ranging between 51 nM and 650 nM) and chloroquine-resistant (Dd2, IC_50_ values ranging between 6.6 nM and 171 nM) *Plasmodium falciparum* cell lines [342].

**Scheme 28:**
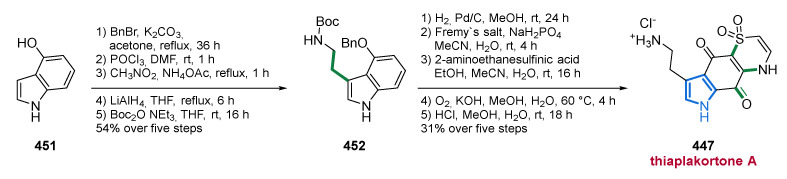
Facile total synthesis of thiaplakortone A (**447**) in a nine-step approach.

In 2014, the first synthesis of thiaplakortone A (**447**) was realized by the Quinn laboratory (Scheme 28) [343]. Starting from commercially available 4-hydroxyindole (**451**), indole **452** was obtained in 54% yield over five steps. Benzyl-deprotection, oxidation, and treatment with 2-aminoethanesulfinic acid, generated an intermediary dihydrothiazine, which, upon saponification and final deprotection, led to the formation of thiaplakortone A (**447**) (Scheme 28) [343].

**Scheme 29:**
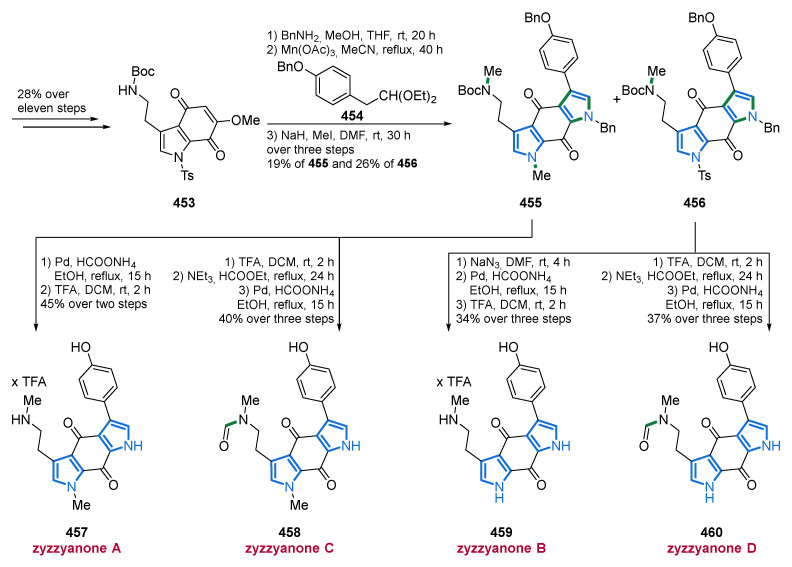
A divergent modular approach providing access to known zyzzyanones A–D (**457**–**460**).

Another subclass of biologically active pyrrole alkaloids is the zyzzyanones, merging the *bis*-pyrrolo functionality together with a pyrroloquinone scaffold. The known zyzzyanones A–D (**457**–**460**), isolated in 1996, were synthesized for the first time by Velu and co-workers in 2013 (Scheme 29) [344]. The authors developed a modular approach that provides access to all four zyzzyanones A–D (**457**–**460**). Starting with the known tosyl-protected indole-4,7-dione (**453**) [345], treatment with benzylamine resulted in amination. The bispyrroloquinone framework was constructed by ring-closing procedure with diethyl acetal **454** and Mn(OAc)_3_. After methylation with MeI, the expected monomethylated amine **455** was obtained alongside the unexpected demethylated amine **456**. Both intermediates **455** and **456** were converted in a series of deprotection and/or formylation reactions to generate the zyzzyanones A–D (**457**–**460**) [344].

**Figure 62:**
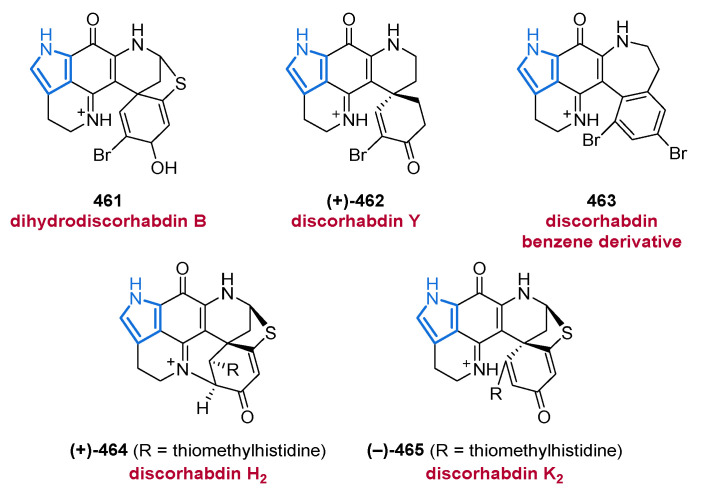
Discorhabdins **461**–**465** resulted from the sponge *Latrunculia* sp. collected in Alaskan and New Zealandian oceans.

**Figure 63:**
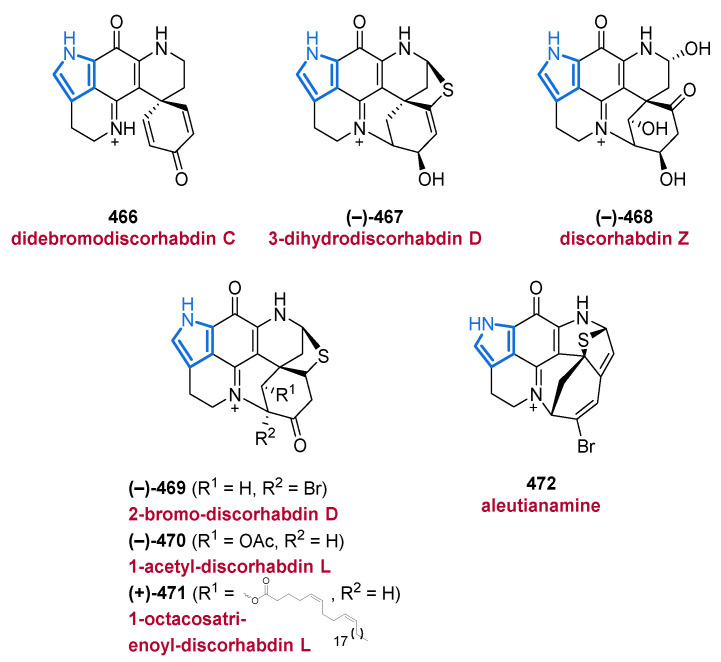
Further discorhabdins **466**–**471**, including a new complex pyrroloiminoquinone **472**.

The discorhabdin journey started with the isolation of the first member of the class, discorhabdin C (its congeners A and B were reported later), in 1986 [346]. In the following years, a dozen more family members were isolated, biologically evaluated, and synthesized. In the decade 2010–2020, 12 further members were identified (Figure 62 and Figure 63). The representatives of this diverse subclass featuring promising bioactivities contain a tetracyclic pyrroloiminoquinone core with a spirocyclic cyclohexadienone moiety. The discorhabdins are thought to be biosynthetically derived from makaluvamines, formed by the coupling of tyramine derivatives with the biosynthetic key precursor of simple pyrroloiminoquinones. In addition, these intermediates also give access to many further subclasses already mentioned [323].

An interesting and at the same time cautionary discovery was made in 2010 when discorhabdin A was isolated for the first time from *Latrunculia oparinae*. In addition to the strong dependence of the color of the solution on the solvent when ethanol (red) and methanol (green) were used, the optical rotation also changed its sign in this solvent switch [347].

Similarly, the Hamann laboratory published the isolation of two new compounds, dihydrodiscorhabdin B (**461**) and discorhabdin Y (**462**) from the Alaskan sponge *Latrunculia* sp. (Figure 62) [348]. Upon structure elucidation using CD and optical rotation, pyrrole **461** showed decomposition, therefore only the absolute stereoinformation of discorhabdin Y (**462**) could be assigned. The azepine derivative **463** was also identified in the same sponge for the first time as a natural product (Figure 62) [348]. Previously, it was only known as a semisynthetic compound, prepared by reduction of natural discorhabdin C and treatment of the resulting dienol with sulfuric acid, initiating an alkenyl (C-20) migration to form discorhabdin benzene derivative (**463**) [349].

Two new diastereomers of discorhabdin H and K, namely discorhabdin H_2_ (**464**) and K_2_ (**465**) were isolated from different sponge populations of *Latrunculia* sp. in 2010 (Figure 62) [350]. Combined structure elucidation was performed by NMR, MS, and extensive ECD-spectroscopy, allowing the assignment of the absolute configuration of the known discorhabdins 2-hydroxy-D, D, H, N, and Q by comparing the recorded with experimental ECD spectra. Furthermore, natural (+)-(6*S*,8*S*)-discorhabdin B was used as a starting point for semi-synthesis to establish the absolute configurations of discorhabdins S, T, and U [350].

The synthetically known didebromodiscorhabdin C (**466**) [351], along with two new discorhabdin derivatives **467** and **468** were isolated for the first time from the sponge *Sceptrella* sp. (Figure 63) [352]. Following previous studies, the absolute configuration was solved by a combination of optical rotation and ECD spectroscopy. In bioactivity studies, average to striking effects were observed against Gram-positive and Gram-negative bacteria (MIC values ranging between 25 µg/mL and >100 µg/mL), as well as against the K562 leukemia cell line and sortase A (IC_50_ values ranging between 2.1 µM and 127.4 µM), with the hemiaminal **468** remarkably showing a more than tenfold higher inhibition than *p*-(hydroxymercury)benzoic acid sodium salt as a positive control [352].

Promising anticancer activity against six cell lines was observed by bioactivity-guided isolation (IC_50_ values of crude extract ranging between 4.0 and 56.2 µg/mL) of three new discorhabdins **469**–**471** from *Latrunculia biformis* (Figure 63) [353]. Discorhabdins **470** and **471** are the first derivatives bearing an ester moiety, containing a simple acetyl group or a C_28_-fatty acid. In the publication, the binding affinity of discorhabdins to anticancer targets (topoisomerase I–II, indoleamine 2,3-dioxygenase) was also determined [353].

Aleutianamine (**472**), the first member of a new class of pyrroloiminoquinone alkaloids, is characterized by a highly fused and multiply bridged heptacyclic ring system and was isolated from the North Pacific sponge *Latrunculia austini* Samaai (Figure 63) [354]. The elucidation of the structure required the combination of preparative spectroscopic methods and advanced computational approaches. It has been supposed that this complex molecular framework is derived from two proteinogenic amino acids, tryptophan, and tyrosine. The authors mentioned that makaluvamine F or discorhabdin A might be the precursors of aleutianamine (**472**), which exhibits promising activity against pancreatic cancer cell lines (IC_50_ values between 25 nM and 1 µM) [354].

### 4.2. Glycosylated Pyrroles

**Figure 64:**
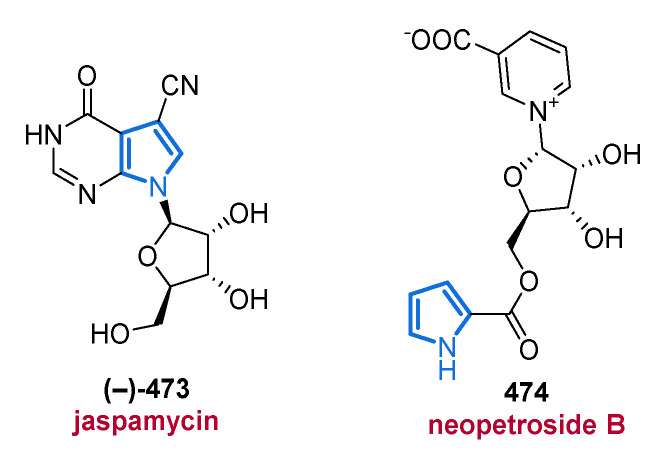
Sugar-substituted marine pyrrole alkaloids **473**–**474**.

In 2016, the known synthetic product jaspamycin (**473**) [355], which is used as a tool compound for the investigation of Parkinson’s disease, was isolated from a marine sponge *Jaspis splendens* and was therefore reported for the first time as a naturally occurring metabolite (Figure 64) [356]. The full stereochemistry of the attached sugar was identical to that of the synthetic product.

Another marine pyrrole alkaloid, neopetroside B (**474**), contains a rare *N*-glycosylpyridinium moiety and was isolated from a *Neopetrosia* sp. sponge in 2015 (Figure 64) [357]. The absolute configuration of compound **474** was determined by comparison with a similar congener from the same work, the sugar unit of which was cleaved off, followed by the synthesis of acetylated (+)-2-octyl glycosides. Comparison of these compounds with authentic samples according to the procedure of Leontein then revealed the d-configuration of the sugar unit [357,358].

**Figure 65:**
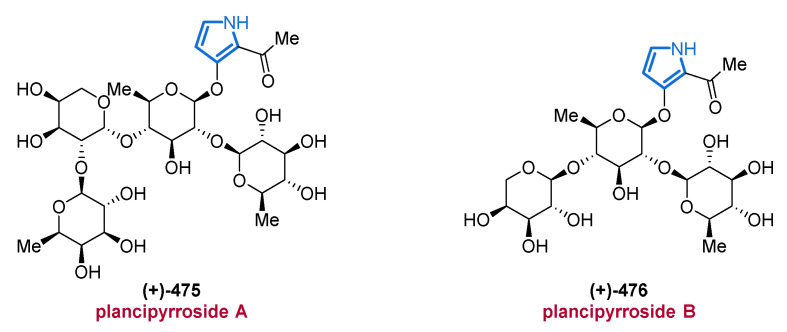
Oligosaccharide-substituted pyrroles **475** and **476** from a marine starfish *Acanthaster planci*.

In 2016, two new pyrrole oligoglycosides, plancipyrrosides A (**475**) and B (**476**) were isolated from the Vietnamese starfish *Acanthaster planci* (Figure 65) [359]. The absolute configuration was determined by comparison with the previously confirmed absolute configurations of the hydrolyzed sugar moieties. Plancipyrroside B (**476**) exhibits a stronger inhibitory effect on lipopolysaccharide-induced nitric oxide production in RAW 264.7 cells (5.94 µM ± 0.34 µM) than plancipyrroside A (16.61 µM ± 1.85 µM) [359].

**Figure 66:**
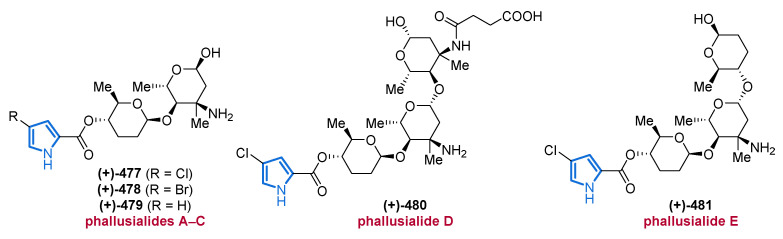
A new group of phallusialides A–D (**477**–**481**) discovered from a marine bacterium.

The sugar-containing pyrrole alkaloids, phallusialides A–E (**477**–**481**) were discovered in a marine bacterium of the genus *Micromonospora* in 2019 (Figure 66) [360]. The relative and absolute configurations of compounds **477**–**481** were determined by ROESY correlations and ECD spectroscopy. While phallusialide A (**477**) and B (**478**) displayed moderate to weak antibacterial activity (MIC values between 32 µg/mL and 64 µg/mL), phallusialides C–E (**479**–**481**) failed to show any detectable activity in the same assay (MIC values > 256 µg/mL). The authors speculated that the lack of halogenation at the pyrrole core of compound **479** and the additional sugar moieties in compounds **480** and **481** were responsible for the inactivity [360].

### 4.3. Peptides

**Figure 67:**
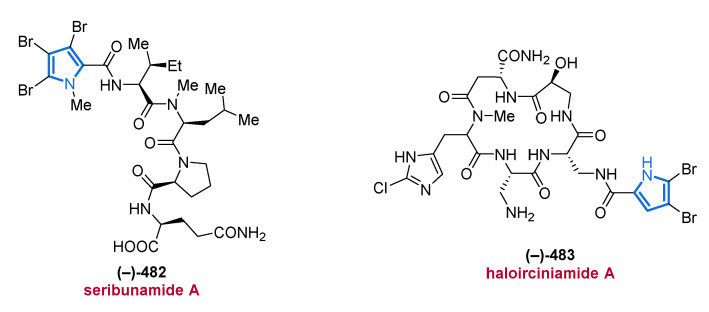
Bromopyrrole peptides **482**–**483** isolated from marine sponges.

Recently, two new unique bromopyrrole peptides, seribunamide A (**482**) and haloirciniamide A (**483**), have been extracted from an Indonesian marine sponge of the genus *Ircinia* (Figure 67) [361]. Their relative and absolute stereochemistries were determined by ROESY correlations and on the basis of derivatization [362] with Marfey’s reagent, 1-fluoro-2,4-dinitrophenyl-5-l-alanine amide. Compounds **482** and **483** did not show any cytotoxicity against several human tumor cell lines [361].

**Figure 68:**
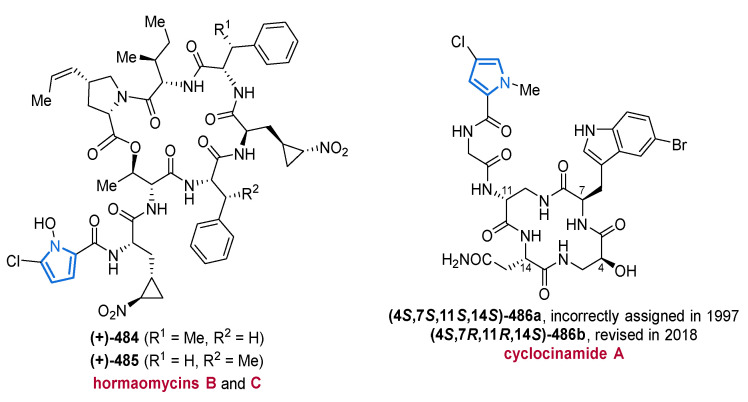
Macrocyclic peptides **484**–**486** containing a pyrrole motif on their N-termini.

In 2015, the structurally unique cyclopeptides hormaomycins B (**484**) and C (**485**) were discovered from a mudflat-derived *Streptomyces* sp. [363] (Figure 68). Both compounds **484** and **485** possess very rare 3-(2-nitrocyclopropyl)alanine units and their absolute configurations were determined by comparing their CD spectra with that of a related hormaomycin. Hormaomycins B (**484**) and C (**485**) showed significant inhibitory effects against various Gram-positive bacteria (MIC values ranging between 0.23 µM and 56 µM), whereas, for Gram-negative bacteria, MIC values between 0.9 µM and 115 µM were determined [363].

At this point, the cyclopeptidic and highly antitumor active pyrrole alkaloid cyclocinamide A (**486b**) should also be mentioned, which was isolated from a sponge of the genus *Psammocinia* by Crews and co-workers back in 1997 [364]. Roughly twenty years later, a total synthesis of **486b** by the Konopelsky group led to the revision of its absolute stereoconfiguration from **486a** to **486b** (Figure 68) [365].

## 5. Conclusions

Pyrrole alkaloids, a very rich family of secondary metabolites widespread among marine organisms, have fascinated the chemical community for many decades. Their large structural variety not only endows them with unique biological activities but also prompts questions concerning the biochemistry of marine life which still require a thorough examination. On the other hand, the seemingly endless number of architectural complex pyrrole alkaloids discovered so far has also led to a considerable number of structural revisions, and the literature is riddled with unknown stereochemistries and inconsistencies in their naming. Synthetic chemists are animated to find new solutions concerning the total syntheses of marine pyrrole alkaloids, thereby providing a larger availability of these compounds which is crucial for the development of derivatives with improved biological activities. New and improved analytical techniques are needed to allow the unambiguous elucidation of relative and absolute configurations of the often-minute quantities of marine natural products available from their producers.

## Figures and Tables

**Table 1:** Summary of published synthesis of lamellarins and related analogs in the decade of 2010–2020.

Year	Author	Lamellarin and Related Congeners	Linear Steps ^i^	Overall Yield
2010	*Iwao* [176]	Lamellarin α 20-sulfate	15	6%
		Lamellarin α 13-sulfate	15	4%
		Lamellarin α 13,20-disulfate	14	9%
2011	*Banwell* [177]	G trimethyl ether	10	3%
		Lamellarin S	11	6%
	*Jia* [178]	Lamellarin D	10	13%
		Lamellarin H	10	13%
		Lamellarin R	5	53%
		Ningalin B	8	14%
2012	*Vazquez* [179]	Lamellarin Q	6	28%
		Lamellarin O	7	25%
	*Banwell* [180]	Lamellarin K	9	57%
		Lamellarin T	9	43%
		Lamellarin U	8	44%
		Lamellarin W	9	45%
2013	*Opatz* [181]	(Dihydro-)/lamellarin η	8/9	62%/57%
		Lamellarin G trimethyl ether	7	69%
	*Iwao* [182]	Lukianol A/B	6/11	36%/11%
2014	*Yamaguchi* [183]	Lamellarin C	9	3%
		Lamellarin I	9	3%
	*Iwao* [184]	Lamellarin N	11;13	42%;34%
		Lamellarin L	13	29%
2015	*Iwao* [185]	Lamellarin L	10	14%
		Lamellarin N	10	12%
	*Opatz* [186]	Lamellarin D trimethyl ether	9	43%
		Lamellarin H	10	41%
	*Ruchirawat* [187]	Aza/lamellarin D	13/13	12%/9%
		Aza/lamellarin N	13/13	28%/15%
	*Tan and Yoshikai* [188]	Lamellarin G trimethyl ether	5	20%
2016	*Iwao* [189]	Lamellarin U	12	5%
	*Yang* [190]	Lamellarin D trimethyl ether	3	8%
		Lamellarin H	4	7%
2017	*Iwao* [191]	Lamellarin N analogues	–	–
		Azalamellarin N analogues	–	–
	*Iwao* [192]	Lamellarin α	12	22%
		Lamellarin η	10	19%
	*Chandrasekhar* [193]	Lamellarin D trimethyl ether	6	44%
		Lamellarin D	7	29%
		Lamellarin H	7	37%
	*Wu* [194]	Lamellarin G trimethyl ether	3	51%
	*Yang* [195]	Lamellarin D trimethyl ether	2	37%
		Lamellarin H	3	31%
		Lamellarin D	6;8	12–14%
		Lamellarin χ	6;8	12–14%
	*Ackermann* [196]	Lamellarin D	10	30%
		Lamellarin H	10	29%
2018	*Opatz* [197]	Lamellarin G trimethyl ether	7;8	19–42%
	*Chiu and Tonks* [198]	Lamellarin R	5	18%
2019	*Donohoe* [199]	Lamellarin D	7	22%
		Lamellarin Q	7	20%
	*Opatz and Michael* [200]	Lamellarin G trimethyl ether	6;7	56–73%
	*Khan* [201]	Lamellarin G trimethyl ether	5	18%
		Lamellarin D trimethyl ether	6	16%
		Lamellarins H, U	7/6	11%/11%
		Dihydro/lamellarin η	7/6	9%/10%
2020	*Saito* [202]	Lamellarin G trimethyl ether	6	26%
		Lamellarin H	8	17%
	*Tsay* [203]	Lamellarin R	3	50%
	*Liou* [204]	Lamellarin R	5	26%
		Lamellarin O	5	10%
		Lukianol A	6	38%
	*Khan* [205]	Lamellarins	6/6/6	21%/21%/21%
		S,Z,G,L,N,D	6/7/7	21%/19%/16%

^i^ The longest linear sequence in the synthesis was counted.
